# Dual-Functional Antibiotic Adjuvant Displays Potency against Complicated Gram-Negative Bacterial Infections and Exhibits Immunomodulatory Properties

**DOI:** 10.1021/acscentsci.4c02060

**Published:** 2025-01-17

**Authors:** Geetika Dhanda, Himani Singh, Abhinav Gupta, Sk Abdul Mohid, Karishma Biswas, Riya Mukherjee, Smriti Mukherjee, Anirban Bhunia, Nisanth N. Nair, Jayanta Haldar

**Affiliations:** † Antimicrobial Research Laboratory, New Chemistry Unit, Jawaharlal Nehru Centre for Advanced Scientific Research, Jakkur, Bengaluru 560064, Karnataka, India; ‡ Department of Chemistry, Indian Institute of Technology Kanpur, Kanpur 208016, India; § Department of Chemical Sciences, Bose Institute, Kolkata 700091, India; ∥ School of Advanced Materials, Jawaharlal Nehru Centre for Advanced Scientific Research, Jakkur, Bengaluru 560064, Karnataka, India

## Abstract

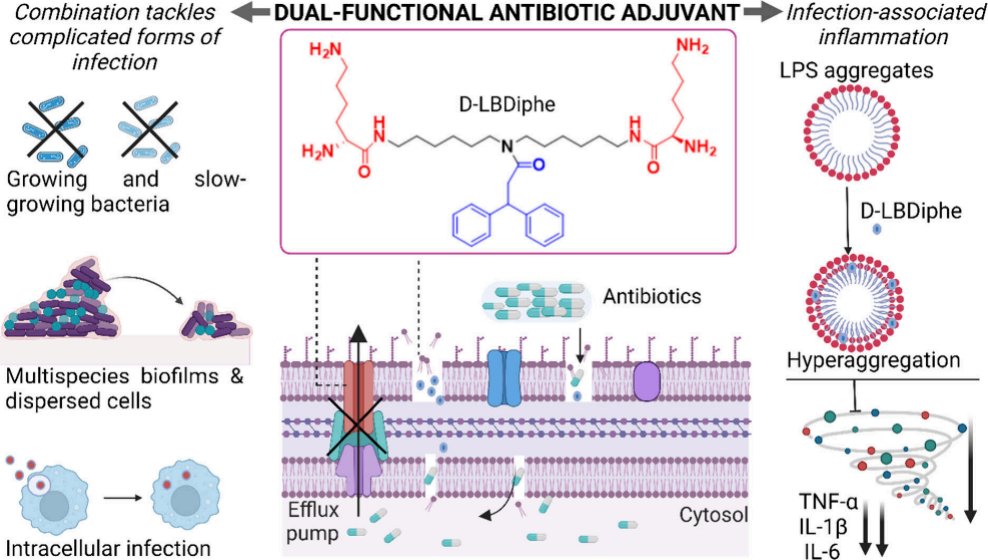

The treatment of Gram-negative bacterial infections is challenged by antibiotic resistance and complicated forms of infection like persistence, multispecies biofilms, intracellular infection, as well as infection-associated hyperinflammation and sepsis. To overcome these challenges, a dual-functional antibiotic adjuvant has been developed as a novel strategy to target complicated forms of bacterial infection and exhibit immunomodulatory properties. The lead adjuvant, D-LBDiphe showed multimodal mechanisms of action like weak outer membrane permeabilization, weak membrane depolarization, and inhibition of efflux machinery, guided primarily by hydrogen bonding and electrostatic interactions, along with weak van der Waals forces. D-LBDiphe potentiated antibiotics up to ∼4100-fold, targeted phenotypic forms of antibiotic tolerance, and revitalized antibiotics against topical and systemic infections of *P. aeruginosa* in mice. The aromatic moiety in D-LBDiphe was instrumental for interaction with lipopolysaccharide (LPS) micelles, and this interaction was the driving factor in reducing pro-inflammatory cytokines by 61.8–79% in mice challenged with LPS. Such multifarious properties of a weak-membrane perturbing, nonactive and nontoxic adjuvant have been discussed for the first time, supported by detailed mechanistic understanding and elucidation of structure-guided properties. This work expands the scope of antibiotic adjuvants and validates them as a promising approach for treatment of complicated bacterial infections and inflammation.

## Abstract 2

D-LBDiphe is a dual-functional antibiotic adjuvant which potentiates antibiotics against complicated Gram-negative bacterial infections and reduces bacterial lipopolysaccharide induced inflammation.

## Introduction

Gram-negative superbugs have been characterized as the most critical infection-causing bacteria by the World Health Organization, with *Acinetobacter baumannii* and *Pseudomonas aeruginosa* as the topmost priority pathogens.^1^ Impermeability posed by the outer membrane, expression of multidrug-resistant efflux pumps, genetic modification of the target and presence of antibiotic-degrading enzymes are all factors which contribute to the multidrug-resistant phenotype of Gram-negative bacteria.^2^ Exacerbating the problem of antimicrobial resistance, phenotypic forms of antibiotic tolerance escape conventional antibiotic therapy and host immune response, often leading to recurrent and persistent infections.^3^ Stationary phase cells, persister cells, biofilms, and intracellular infection constitute phenotypic forms of antibiotic tolerance. Seeing the emergence of drug-resistant pathogens, antibiotic tolerance, and sluggish development of new antibiotics, it is pertinent to look at orthogonal and complementary strategies to conventional drug discovery.

Antibiotic adjuvants are compounds with little or no antibiotic activity which enhance antibiotic action by targeting resistance elements.^4−6^ The success of β-lactamase inhibitors in combination with β-lactam antibiotics over the past decades validates the efficacy, robustness and value of this concept.^4^ The use of an antibiotic with adjuvant decreases the odds of resistance development.^6^ Efforts toward development of other classes of adjuvants targeting various resistance mechanisms in bacteria have surfaced immensely; however, β-lactamase adjuvants are the only approved adjuvants to date.^4^

Membrane-targeting compounds hold immense potential to be developed as adjuvants to revitalize existing antibiotics^2,7−14^ by overcoming membrane-associated resistance elements like permeability barrier and antibiotic efflux, and mildly active membrane-targeting compounds have been used at subinhibitory concentrations in combination with antibiotics to combat Gram-negative bacteria.^9^ However, they suffer from challenges of toxicity, instability in physiological fluids, and further clinical development. Despite the challenges associated with membrane-targeting adjuvants, SPR741 is one example which has made it to clinical trials;^15^ however, the holistic success of this class of antibiotic adjuvants depends on detailed structure–activity relationship studies and streamlined biological optimizations. There is immense scope for the development and mechanistic understanding of membrane-targeting adjuvants.

Additionally, inflammation accompanied in the aftermath of infection often leads to hyperinflammation and sepsis, which results in multiorgan failure and death.^16^ Various pathogen associated molecular patterns (PAMPs) like lipopolysaccharide (LPS) can interact with receptors of host immune cells, eliciting a strong immune response.^17^ While inflammatory agonists are required in the beginning of infection, there is a pertinent need to invest in immunomodulators and anti-inflammatory compounds to protect from sepsis. There are examples of antibiotics, synthetic compounds and antimicrobial peptides possessing immunomodulatory properties in the literature;^18−21^ however, dual-functional antibiotic adjuvants without any antibacterial efficacy but possessing both antibiotic potentiating and immunomodulatory properties are less known.^2^

Among very few structural optimizations carried out for antibiotic adjuvants, cyclic hydrophobic moieties and cationic charges have been shown to be important structural parameters in the design of nontoxic and nonactive small molecular membrane targeting adjuvants.^22,23^ The effect of linker length and flexibility has been explored for the preclinical development of pentamidine analogues and structure–activity studies of bis-amidines.^14,22^ However, the combined effect of backbone lipophilicity, number of amine groups, and presence of amide bonds has not been explored for the structure–activity relationship of membrane-perturbing adjuvants. Moreover, a single membrane-perturbing adjuvant with multifaceted properties *vis-à-vis* antibiotic potentiation, activity against phenotypic forms of antibiotic tolerance and host-modulating effects has not been reported. Detailed structure–activity and toxicity studies, identification of the role of optimum structural parameters, and complementary mechanistic studies can assist in the discovery of a multifaceted antibiotic adjuvant that tackles all the above-mentioned aspects of bacterial infection, while also acting as an immunomodulatory agent.

Toward this, triamine analogues with different skeletal backbone and appendage modifications were synthesized to understand the role of the hydrophobic backbone, amine groups, amide bonds, and cyclic hydrophobic pendants in the design. The triamine scaffold was chosen due to the presence of amine groups, which will be positively charged at physiological pH, which will help in interaction with negatively charged components of the bacterial membrane. Also, the amine groups can be synthetically modified for appendage modifications. A bis(hexamethylene) triamine analogue, D-LBDiphe, depicted minimum toxicity and superior potentiation with different classes of antibiotics. Its detailed mechanistic properties were investigated through experiments as well as molecular dynamics simulation studies to gain an understanding of the interaction of the molecule with the constituents of the bacterial envelope. The metabolism-independent therapeutic window of the lead compound and its combinations has been explored against stationary phase bacteria, persisters and multispecies biofilms, and activity was also evaluated against intracellular *P. aeruginosa* infection. Interaction of D-LBDiphe with LPS and its subsequent effect on the host immune response were also evaluated. The role of structural features in obtaining immunomodulatory properties has been identified through STD NMR and other biophysical studies. Further, the biocompatibility of D-LBDiphe and combination therapy has been evaluated *in vivo*, and its potential as a preclinical candidate was validated in murine acute skin infection and pulmonary infection models of *P. aeruginosa*.

## Results and Discussion

### Rational Design of Small Molecular Adjuvants

Adjuvants with cyclic hydrophobic pendants and two-three amines (NNaph, NDiphe and NAda) with calculated logP values in the range of 0.9–2.7 (Table S1) had been synthesized and assessed for potentiation properties against Gram-negative bacteria.^23^ However, the high concentrations required for potentiation and the inability of NDiphe to potentiate fusidic acid against New-Delhi metallo-β-lactamase-producing bacteria prompted us to synthesize new sets of compounds with variations in the number of amines (2–5), number of amide bonds, backbone lipophilicity, and cyclic hydrophobic pendants. The presence of amines, which are protonated at physiological pH, and amide bonds enables electrostatic and hydrogen bonding interactions with the negatively charged phospholipids and LPS of bacterial membrane.^24^ Moreover, the lipophilic part from the backbone as well as the cyclic pendant groups enables interactions with lipophilic domains of membrane lipids.

**Figure 1:**
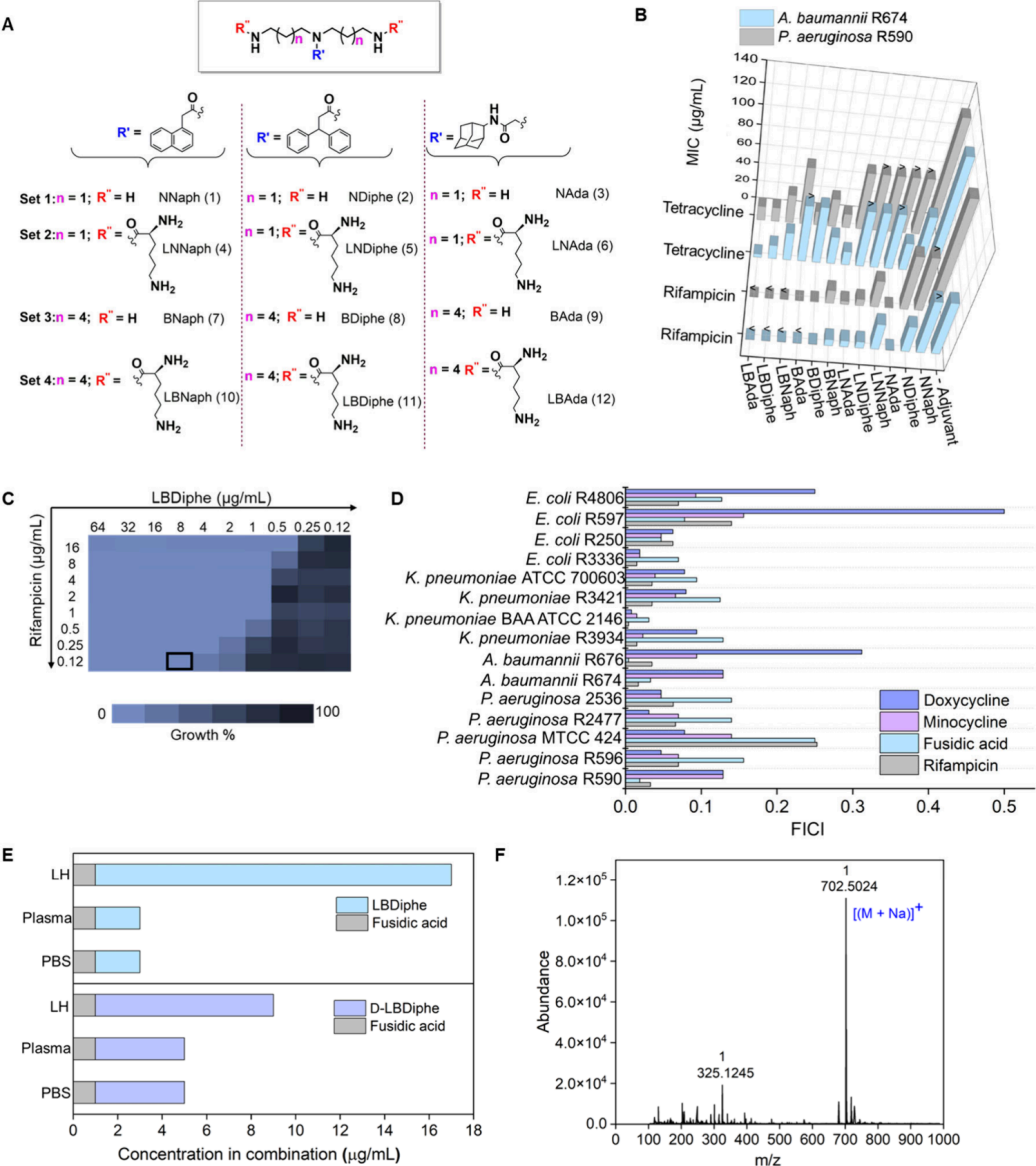
Design rationale and initial optimization studies. (A) Design rationale and summary of compounds used in the study. Twelve different compounds on a norspermidine or bis(hexamethylene) triamine backbone by varying the number of amine groups and the pendant cyclic hydrophobic moiety were used. (B) MIC of antibiotics in the presence and absence of adjuvants against *A. baumannii* R674 (AB R674) and *P. aeruginosa* R590 (PA R590). For rifampicin, data is presented at an adjuvant concentration of 16 μg/mL. For tetracycline, data is presented at an adjuvant concentration of 64 μg/mL except for BAda and LBAda, whose concentrations are 16 μg/mL. (C) Representative checker board of LBDiphe and rifampicin against *A. baumannii* R674. The marked rectangle indicates the optimum concentration of the adjuvant and antibiotic to inhibit bacterial growth. (D) Fractional inhibitory indices for the combinations in Table S3. (E) Concentration of components in combination against AB R674 after incubation of LBDiphe and D-LBDiphe with plasma and liver homogenate (LH), (F) HRMS spectrum of the supernatant after incubation of D-LBDiphe and LH.

The variation in the cyclic hydrophobic pendant was carried out by appending naphthyl, 3,3-diphenyl propanoyl and adamantane groups to the secondary amine of the triamine backbone. NNaph, NDiphe and NAda constituted the first set of compounds with a norspermidine backbone (Figure 1A). To achieve a second set with an increase in the number of cationic charges and amide bonds, l-lysine conjugated norspermidine derivatives (Set 2: LNNaph, LNDiphe and LNAda) with lower logP values (0.15–1.96) were prepared (Scheme S1). In order to achieve skeletal backbone diversity, bis(hexamethylene) triamine derivatives (Set 3: BNaph, BDiphe and BAda) (Scheme S2) with higher logP values in the range of 3.3–5.1 were synthesized. A final set of compounds (Set 4: LBNaph, LBDiphe and LBAda) with increased hydrophobic backbone, amine groups, and amide bonds, and logP in the range of 2.5–4.3 was synthesized (Scheme S3).

### LBDiphe as a Generic Nontoxic Antibiotic Adjuvant with High Potentiation Ability toward Gram-Negative Superbugs

#### Structure–Activity and Toxicity Relationship Studies

The four sets of compounds were tested for antibacterial activity against *A. baumannii* and *P. aeruginosa* (Figure S1A). Set 1 (NNaph, NDiphe and NAda) exhibited MIC > 512 μg/mL against both the bacterial clinical isolates, indicating no bacterial activity.^17^ Set 2 compounds (LNNaph, LNDiphe and LNAda) did not exhibit antibacterial activity (MICs > 512 μg/mL). Set 3 compounds (BNaph, BDiphe and BAda) with increased logP values in the range of 3.3–5.1 showed reduced MICs of 128–256 μg/mL in the case of BDiphe and 64 μg/mL in the case of BAda, indicating more interaction with the bacterial membrane, possibly due to increased lipophilicity. Only BNaph with the least logP value in Set 3 showed MICs > 512 μg/mL. In moving from Set 3 to Set 4, the number of amines increased to 4–5 and the MICs started shooting up again (64–512 μg/mL), indicating the balance obtained by increasing backbone lipophilicity, number of cationic charges, and number of amide bonds simultaneously (Figure S1A). Toxicity against human RBCs revealed no hemolysis by any of the compounds, as the viability of RBCs was 100% even at the highest tested concentration of 1024 μg/mL, for all the 12 compounds (Figure S1B). From these studies, we infer that the choice of pendant cyclic hydrophobic moieties in the design retained the nontoxic nature of the compounds, even after skeletal and side-appendage modifications. However, it was observed that a perfect balance of lipophilicity and cationic charges was required to maintain the nonactive to minimally active nature of the compounds, which is preferred to ensure optimum and selective interaction with bacterial membrane lipids.

#### Potentiation Ability of the Synthesized Adjuvants

The compounds were evaluated for their potential to repurpose rifampicin and tetracycline against *A. baumannii* and *P. aeruginosa* by performing checkerboard assays. Gram-negative superbugs are resistant to these antibiotics due to membrane-associated resistance elements like permeability barrier and efflux pumps.^25,26^ The MICs of the antibiotics in the presence and absence of the adjuvants were plotted (Figure 1B, Table S2) by analyzing checkerboard assays (Figure 1C). The extent of potentiation, at the same concentration of the adjuvant increased upon systematic modifications in the design, leading to an increase in number of amines, backbone lipophilicity and number of amide bonds. In the first set of norspermidine derivatives bearing 2–3 amines, NAda was the best adjuvant, showing 128-fold potentiation of rifampicin; however, only 2-fold potentiation of tetracycline was observed. NNaph and NDiphe showed only 2–4-fold potentiation of both the antibiotics. Lysine-conjugated norspermidine derivatives (Set 2) showed slightly better potentiation where all the compounds showed 2–32-fold potentiation of both antibiotics. The increase in number of charges and amide bonds improved the potentiation by compounds bearing aromatic pendants while the potentiation effect of the adamantane-containing pendant decreased slightly. This might be due to a difference in the interaction between the adjuvants and bacterial membrane lipids, resulting from different structures and logP values of the compounds (Table S1). LNAda had a logP of 0.15, which might reduce lipophilic interactions with membrane lipids. The bis(hexamethylene) analogues showed 2–1024-fold potentiation; however, BDiphe and BAda were slightly superior adjuvants. Tetracycline potentiation was still only 2–4-fold in this case. Set 4 compounds were the best potentiators, showing 128- to >1024-fold potentiation of rifampicin and 4–32-fold potentiation of tetracycline (Figure 1B). Thus, it was observed that a perfect balance of backbone lipophilicity, pendant cyclic hydrophobic pendants, amine groups, and amide bonds was needed to show the best potentiation factors. LBNaph, LBDiphe and LBAda were further evaluated for combination with fusidic acid, minocycline and rifampicin against four multidrug-resistant clinical isolates including NDM-producing strains (Figure S2). LBDiphe proved to be the most superior compound, exhibiting 8–1024-fold potentiation of the three antibiotics at optimal concentrations between 2 and 64 μg/mL.

Broad-spectrum potentiation of LBDiphe was tested against 15 strains of Gram-negative superbugs in combination with 5 antibiotics: rifampicin, minocycline, doxycycline, fusidic acid, and linezolid (Table S3). Briefly, good potentiation of rifampicin, fusidic acid, doxycycline and minocycline was observed against all clinical isolates with up to 4096-fold potentiation effect. Fractional inhibitory concentration indices (FICIs) were calculated for each combination (Figure 1D). FICI values less than or equal to 0.5 indicate synergy.^23^ LBDiphe potentiated rifampicin against all the resistant strains of Gram-negative bacteria with FICIs in the range of <0.004 to 0.253. With fusidic acid, the FICI values ranged between 0.004–0.25 across all the 15 strains. The FICIs were between <0.019–0.156 for minocycline and <0.008–0.5 for doxycycline. LBDiphe did not potentiate linezolid against all tested strains, especially *K. pneumoniae* isolates. The best FICIs observed for linezolid were 0.156–0.56 (Table S3).

The best combinations led to complete bacterial killing in 7–12 h against the tested strains of *P. aeruginosa* and *K. pneumoniae* in combination with fusidic acid, rifampicin and minocycline (Figure S3A–C). The slow kinetics of the combination further reinforces the nonactive nature of the adjuvant at the concentrations used in combination. It was hypothesized that the adjuvants only potentiate antibiotics, potentially by overcoming mechanisms of resistance, and the antibiotic shows the primary antibacterial effect. This observation also hints at the weak membrane-perturbing nature of the adjuvants rather than a defined membrane-disrupting mechanism, which is validated in later sections.

#### Stability of LBDiphe in Physiological Fluids and Final Optimization Studies

In order to achieve efficacy *in vivo*, it is important that the bioactive molecule or adjuvant is stable in physiological fluids. As l-amino acids can be cleaved by peptidases in the body, a d-analogue of LBDiphe (D-LBDiphe) was synthesized (Figure S3D, Scheme S4). The potentiation ability of both the compounds was tested against *A. baumannii* R674 after incubation with 50% human plasma and 50% mouse liver homogenate. Plasma and liver contain various enzymes like amidases and esterases which can cleave the amide or ester bonds present in drugs.^27^ Preincubation of both the analogues with plasma revealed no difference in the potentiating activity (Figure 1E). However, upon incubation with liver homogenate, an 8-fold increased concentration of LBDiphe was required to cause the same level of potentiation; however, only a 2-fold increase was observed for D-LBDiphe (Figure 1E).

Analysis by mass spectrometry revealed that the l-derivative showed peaks for fragments corresponding to cleavage of one or both lysine residues (Figure S3E), whereas the compound peak was intact for D-LBDiphe (Figure 1F). There was no considerable difference in the levels of potentiation by D-LBDiphe (Table 1). Potentiation (16–128-fold) was also observed with minocycline and fusidic acid against the virulent strain *P. aeruginosa* PAO1 (Table 1). As the adjuvant is expected to be a nonspecific membrane-interacting compound, the change in stereochemistry of lysine is not expected to affect its interaction with the membrane. D-LBDiphe, alone and in combination with antibiotics, did not exhibit any added toxicity toward human peripheral blood mononuclear cells and HEK cells, making it the optimized lead for further investigations (Figures S5 and S6).

**Table 1:** Potentiation Ability of D-LBDiphe against *A. baumannii* and *P. aeruginosa*

Bacterial strain and antibiotics	MIC_antibiotic_ (μg/mL)	MIC of antibiotic (μg/mL) in presence of D-LBDiphe	Concentration of D-LBDiphe (μg/mL) required	FICI^a^
***A. baumannii*R674**				
Doxycycline	64	4	2	0.066
Minocycline	8	1	4	0.133
Fusidic acid	64	0.125	16	0.033
Rifampicin	64	0.125	8	0.017
				
***P. aeruginosa*R590**				
Doxycycline	64	4	2	0.066
Minocycline	8	1	4	0.133
Fusidic acid	64	2	4	0.039
Rifampicin	64–128	0.125	16	0.032–0.033
				
***P. aeruginosa*PAO1**				
Minocycline	8	0.5	8	<0.078
Fusidic acid	>512	4–8	64	<0.13–0.14

a FICI stands for Fractional inhibitory concentration index.

### D-LBDiphe Shows Weak Membrane-Perturbing Properties and Inhibits Efflux Primarily Guided by Hydrogen Bonding and Electrostatic Interactions with Membrane Lipids

D-LBDiphe has been designed to incorporate soft positive charges and optimum hydrophobic pendant to enable noncovalent interactions with bacterial envelope lipids and components like phosphatidyl glycerol, phosphatidyl ethanolamine, and LPS, resulting in optimum membrane perturbing properties. Since the adjuvant was seen to potentiate antibiotics which are rendered ineffective because of efflux or permeability barrier of the outer membrane in Gram-negative bacteria, the next step was to delve into the membrane-perturbing properties of D-LBDiphe and elucidate the exact molecular interactions responsible for this perturbation.

#### Weak Membrane-Perturbing Properties of D-LBDiphe

**Figure 2:**
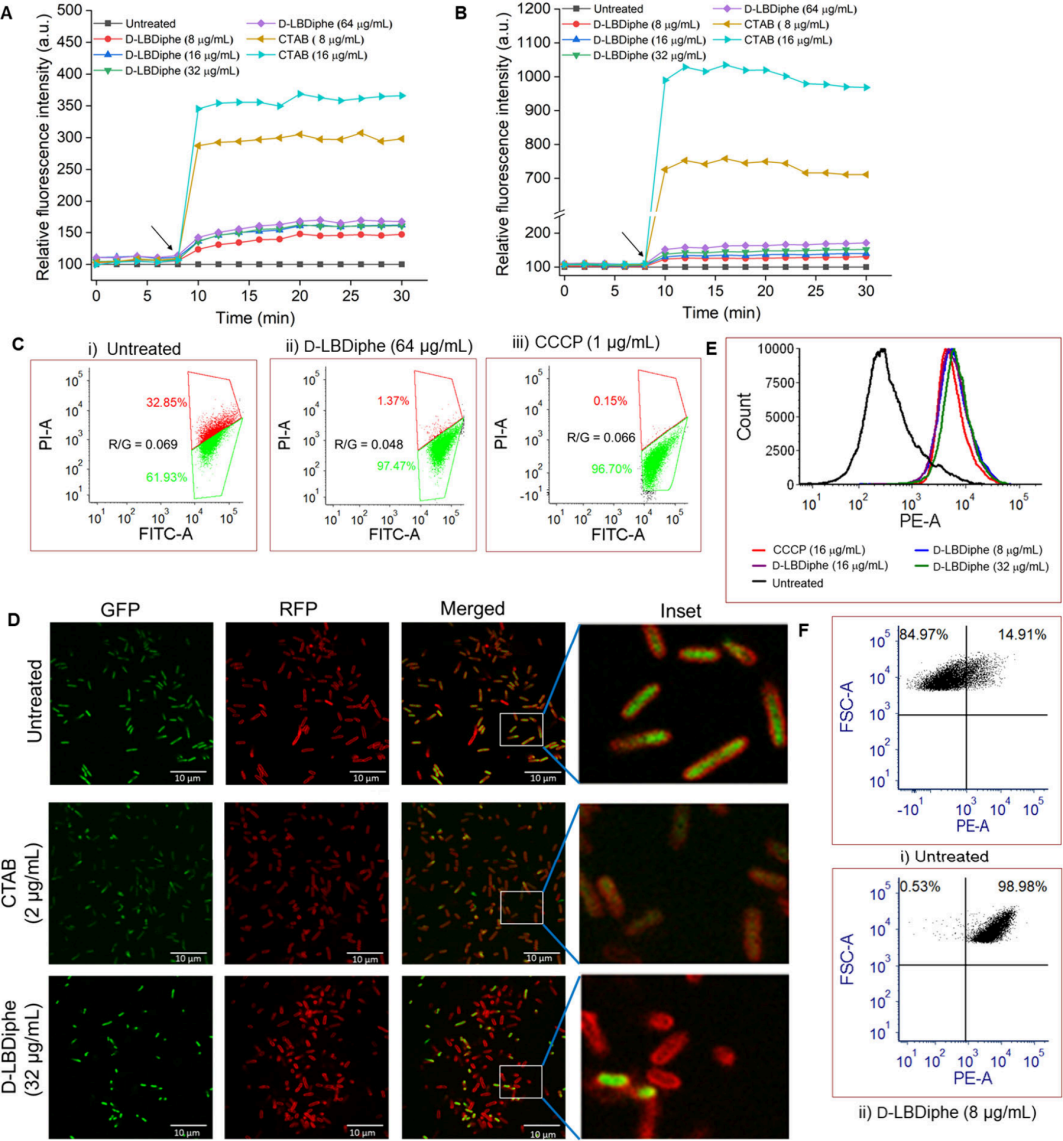
Mechanism of action of D-LBDiphe. (A) Outer membrane-permeabilization of D-LBDiphe and CTAB using the NPN assay. (B) Membrane depolarization of D-LBDiphe and CTAB using the DiSC_3_(5) assay. The arrow indicates the time of compound addition. (C) Assessment of membrane potential in PA R590. (i) Untreated control, (ii) upon treatment with D-LBDiphe (64 μg/mL) and (iii) upon treatment with CCCP (1 μg/mL). R/G represents the ratio of the mean intensities of the red aggregates to the green monomer. PI-A stands for propidium iodide-area to detect red fluorescence, and FITC-A stands for fluorescein isothiocyanate-area for detecting green fluorescence. (D) Microscopy studies using FM4-64 dye for membrane staining in GFP-tagged PAO1. GFP stands for green fluorescent protein, and RFP stands for red fluorescent protein. Scale bar = 10 μm. The inset images are magnified post image processing to visualize the staining in cells. (E) Inhibition of efflux machinery in PA R590 by D-LBDiphe using flow cytometric analysis represented using a histogram plot showing EtBr-positive cells in the presence or absence of various treatments. (F) Dot plots to show EtBr-positive and EtBr-negative cells in (i) untreated control and (ii) upon treatment with D-LBDiphe (8 μg/mL). PE-A stands for the R-phycoerythrin-area channel for EtBr. FSC-A stands for forward scatter-area.

D-LBDiphe was tested for membrane perturbation by assessing outer membrane permeabilization and membrane depolarization through fluorescence assays against *P. aeruginosa* R590 using known probes.^28^ DiSC_3_ (5) dye is known to release into solution and show increased fluorescence intensity upon cytoplasmic membrane depolarization.^29^ An intact outer membrane excludes substances such as *N*-phenyl-1-naphthylamine (NPN), but a perturbed outer membrane causes the entry of NPN molecules into the phospholipid layer, thereby showing an increase in fluorescence. D-LBDiphe (8–64 μg/mL) showed a slight concentration-dependent increase in the fluorescence intensity for both the assays as compared to the surfactant CTAB (8–16 μg/mL), indicating a weak perturbation of the membrane (Figure 2A–B). The reduction in the membrane potential (Δψ) was also investigated through flow cytometry by using the 3,3′-diethyloxacarbocyanine iodide (DiOC_2_ (**3**)) dye (Figures 2C and S7A). DiOC_2_ (**3**) is known to form red self-associated J-aggregates upon accumulating in the bacterial cell in a membrane-potential dependent manner.^30^ The percentage of J-aggregates reduced to ∼1.4–2.2% upon cotreatment with D-LBDiphe (16–64 μg/mL) from ∼33% in the untreated control, in a concentration-dependent manner (Figures 2C and S7A). The R/G ratio also reduced in the samples treated with D-LBDiphe to 0.048–0.054 from 0.069 in the untreated control (Figures 2C and S7A), signifying a reduction in the membrane potential of bacterial cells upon treatment with the adjuvant D-LBDiphe.

To visualize the condition of the bacterial membrane after treatment with D-LBDiphe, FM 4-64 dye was employed. D-LBDiphe (32 μg/mL) had well-defined membrane stained by FM 4-64, similar to the untreated control and in contrast to the dispersed fluorescence observed after treatment with surfactant CTAB at 2 μg/mL (Figure 2D).

The weak membrane perturbation was also proven in a model Gram-negative bacterial membrane mimic of Gram-negative bacteria by evaluating calcein release upon D-LBDiphe treatment, which was only 11% compared to the surfactant control Triton-X used in this study (Figure S7C). Overall, D-LBDiphe did not exhibit membrane-disruptive properties like known surfactants, making it nontoxic, and showed weak membrane-perturbing properties which can be the primary mechanism for potentiation of antibiotics which are rendered ineffective due to permeability barrier and/or efflux.

#### Inhibition of Efflux Machinery by D-LBDiphe

Gram-negative bacteria express different types of efflux pumps, which have been classified into various families.^31^ Except for the ATP-binding cassette (ABC) family, the functioning of all other families of efflux pumps, like resistance-nodulation-cell division (RND) and major facilitator superfamily (MFS), is dependent on the proton gradient or the membrane potential across the bacterial cell. Since the optimized adjuvant D-LBDiphe depolarized the membrane in bacteria, its effect on the working of efflux pumps was determined. Ethidium bromide (EtBr), a substrate of various MFS and RND efflux pumps in multidrug-resistant bacteria,^32^ was used for the readout. The difference in the number of cells that stained positive for EtBr was ascertained through flow cytometry, where the histogram plot showed more EtBr-positive cells for D-LBDiphe treated *P. aeruginosa* as compared to the untreated control (Figure 2E). This signifies reduced efflux of EtBr in the adjuvant treated cells. The geometric mean intensity of the EtBr positive cells in the untreated control was less than half of that observed in the D-LBDiphe treated samples (Figure S7B).

The untreated control and D-LBDiphe treated samples at 8–16 μg/mL showed 14.9% cells and ∼99% cells as EtBr-positive, respectively (Figures 2F and S7B). This signifies that D-LBDiphe inhibited the efflux machinery in bacteria, which can be a result of a depolarized membrane potential (Figures 2B–C). Inhibition of efflux is expected to be an important mechanism of potentiation observed for antibiotics which are extruded out by efflux, like minocycline, doxycycline and linezolid.

#### Interaction of D-LBDiphe with Membrane Lipids

**Figure 3:**
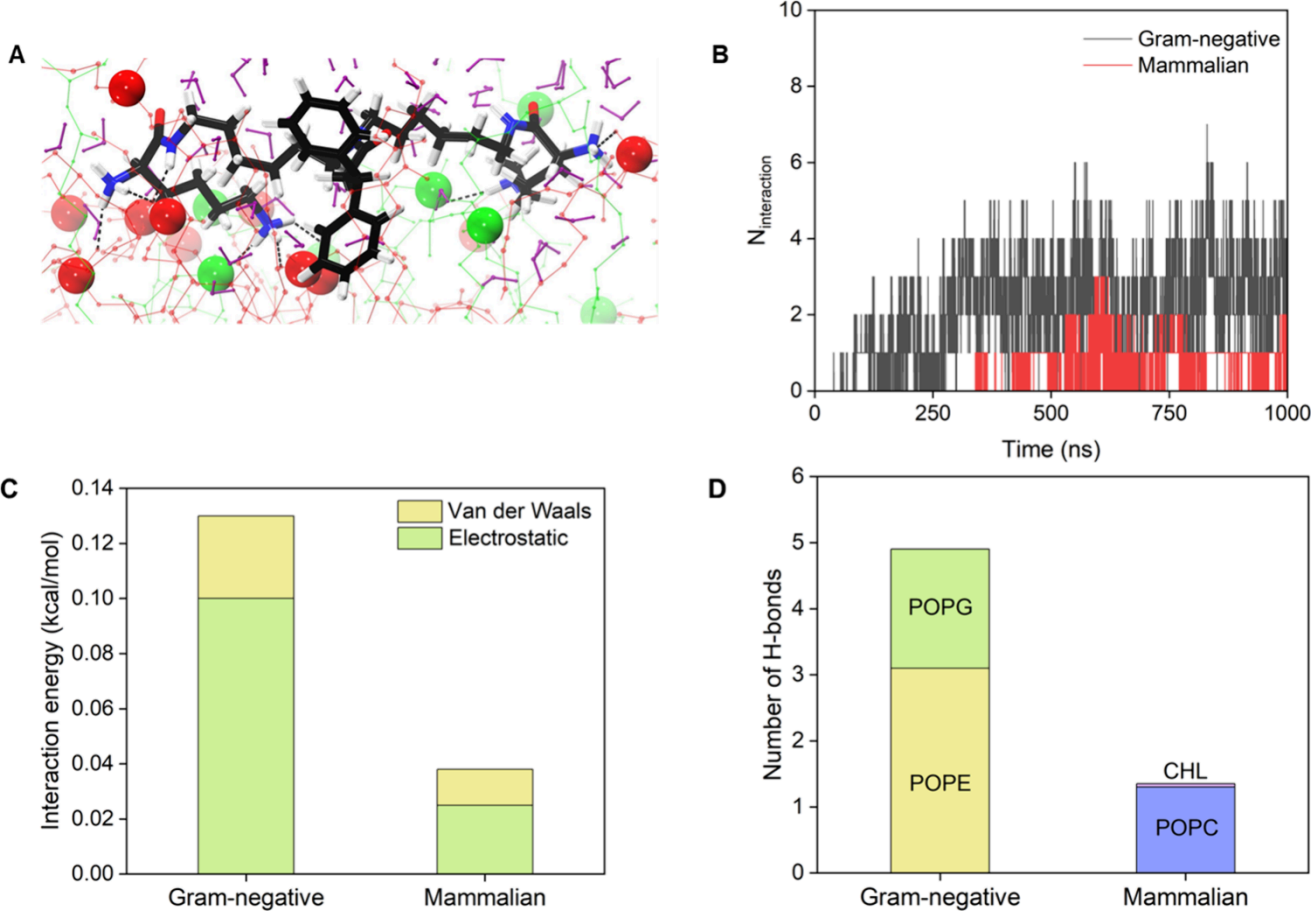
(A) A closeup view of the D-LBDiphe molecule interacting with the phospholipids at the water–membrane interface of the Gram-negative membrane. Hydrogen bonding interactions (dotted black lines), D-LBDiphe (stick; O: red, N: blue, C: black, H: white), lipids (lines; red for POPE, green for POPG), lipid-phosphate head groups (spheres; red for POPE, green for POPG) and water molecules (ball–stick in violet) are shown. (B) The number of D-LBDiphe molecules interacting with the membranes (N_interaction_) during the MD simulation with Gram-negative and mammalian membranes. (C) Electrostatic and van der Waals energy contributions in the average interaction energy of D-LBDiphe and the lipids. (D) The average number of hydrogen bonds between D-LBDiphe and the lipid head-groups computed along the 1000 ns trajectory.

To get a molecular level understanding of the interaction between D-LBDiphe and the lipids of the bacterial and mammalian membranes, we carried out molecular dynamics (MD) simulations. We started the simulations with D-LBDiphe molecules randomly placed in an aqueous solution near the Gram-negative membrane surface. The Gram-negative membrane constituted the lipids POPE:POPG = 3:1 (POPE = 1-palmitoyl-2-oleoylphosphatidylethanolamine; POPG = 1-palmitoyl-2-oleoylphosphatidylglycerol);^33^Figure S8A). We observed from the trajectory of the simulation that the amine and amide groups of D-LBDiphe molecules form hydrogen bonding interactions with the head groups of POPE and POPG at the water–membrane interface (Figures 3A and S8B). Subsequently, the 3,3′-diphenyl propanoyl group interacts with lipid acyl chains, leading the phenyl groups of D-LBDiphe molecules to insert into the bilayer (Figures 3A and S8B). D-LBDiphe molecules were largely localized at the membrane interface and did not fully insert into the membrane (Figure S8C), which corroborates with its weak membrane-perturbing nature (Figure 2). The molecules kept associating and dissociating from the surface (Figure S9A). On an average, four out of the ten molecules were always interacting on the surface of the membrane leaflet (Figure 3B). A similar simulation was also carried out with D-LBDiphe molecules and a mammalian membrane. The mammalian membrane was modeled using the lipid composition POPC:CHL = 2.3:1 (POPC = 1-palmitoyl-2-oleoyl-phosphatildycholine; CHL = Cholesterol);^34^Figure S8D). Only 1 out of 10 molecules was seen to interact with the membrane surface (Figure S8E–F).

From the average interaction energy computed between the lipids and D-LBDiphe molecules, it was seen that the average electrostatic energy has a higher contribution compared with the average van der Waals energy (Figure 3C). This can be understood by the interactions involved, especially between lipid head groups and the amine and amide groups of the D-LBDiphe molecule (Figure 3A). The number of hydrogen bonds between D-LBDiphe and POPE was higher than that with POPG (Figure 3D). Attractive electrostatic interactions could be a major driving force for the D-LBDiphe molecules to move toward the interface of the Gram-negative membrane (Figures S9A, S9C). For the mammalian membrane, the attractive electrostatic interactions between the membrane and the D-LBDiphe molecules were much smaller (Figures 3C and S9C). This is expected considering the neutral nature of the mammalian membrane. The number of H-bonds between D-LBDiphe and the mammalian membrane is very few and is mainly due to the interaction of D-LBDiphe with POPC rather than CHL (Figures 3D and S8E).

On the contrary, CTAB molecules aggregated in solution and, subsequently, the aggregate interacted with the membrane–water interface; after which the aggregate inserted and dissociated, and dissociated molecules were dispersed within the Gram-negative and mammalian membranes (Figures S10 and S11). For both the membranes, the interaction energy had a significant contribution from van der Waals forces. The mode of insertion and dispersion of CTAB molecules within the membranes, as observed in our simulations, could thus be correlated to its strong membrane-perturbing nature, activity against bacteria and toxicity against mammalian cells.^23^ In conclusion, the correct balance of electrostatic and van der Waals interaction energy with higher contribution from electrostatic interactions could be the key to D-LBDiphe’s selectivity and distinct weak membrane-perturbing nature. This was modulated by lipophilic and hydrophilic structural moieties in the molecular design, making it different from known lipophilic cationic molecules like CTAB.

### Efficacy against Growth-Restricted Phases of Bacteria and Disruption of Multispecies Biofilms by Combinations of D-LBDiphe with Antibiotics

Mixed species biofilms of Gram-positive and Gram-negative bacteria are prevalent in pulmonary infections and diseases like cystic fibrosis, often making treatment challenging.^35,36^ Conventional therapies are not effective in penetrating the physical barrier presented by the biofilm matrix, let alone combating different phenotypes of bacteria within the biofilm or targeting the complex communication network in a mixed species biofilm.^37,38^ As growth-restricted phases of bacteria like the stationary phase and persister cells are present in biofilms, the activity of optimized combinations was tested against slow-growing phases of Gram-positive and Gram-negative bacteria. Complete bacterial eradication of the stationary phase of *P. aeruginosa* and MRSA was observed in 4 and 24 h, respectively, by combination of fusidic acid and D-LBDiphe (Figure S12A–B). Ampicillin-generated *S. aureus*persisters were also eradicated completely in 24 h by the same combination (Figure S12C). Considerable reduction of bacterial burden was also observed upon individual treatment with D-LBDiphe for the MRSA stationary phase and *S. aureus* persister cells (Figures S12B–C). D-LBDiphe exhibited weak membrane-perturbing properties against stationary phase *P. aeruginosa* and MRSA (Figure S12D–H), which might be the reason for its metabolism-independent therapeutic window against antibiotic-tolerant cells. The bactericidal activity of fusidic acid in combination with D-LBDiphe might be due to the combined weak membrane-perturbing effect of the adjuvant along with protein synthesis inhibition by the antibiotic. Seeing the activity of the adjuvant against metabolically repressed cells and its probable interactions with the negatively charged constituents of the extracellular polymeric matrix of biofilms, the activity of combinations against mature biofilms of Gram-positive and Gram-negative bacteria was tested. Higher concentrations than the FICs against planktonic bacteria were used due to the high intrinsic tolerance profiles of the biofilms. Against mature biofilms of *A. baumannii*, two doses of treatment of combination of D-LBDiphe (64 μg/mL) and fusidic acid (16 μg/mL) led to 3.2 log CFU/mL reduction, which was much more significant than the individual treatments at the same concentrations (less than 1 log CFU/mL), single dose combination treatment (2.4 log CFU/mL) and treatment with the last resort antibiotic, colistin, at 16 μg/mL (∼2 log CFU/mL) (Figure S13A). A 1.7 log CFU/mL reduction was observed with the combination treatment of minocycline (4 μg/mL) and D-LBDiphe (32 μg/mL) (Figure S13A). Moreover, ∼4 log CFU/mL and ∼1.7 log CFU/mL reduction was observed in the dispersed cell count after two doses of treatment with fusidic acid + D-LBDiphe, and minocycline + D-LBDiphe treatments, respectively (Figure S13B), which was superior to a single dose of combinations and individual treatments. The combination of fusidic acid and D-LBDiphe was superior to colistin in reduction of biofilm as well as dispersed cell burden. The efficacy of combinations to disrupt *A. baumannii* biofilms was also confirmed by confocal microscopy, where a reduction of 10.5–11 μm in thickness of biofilms was observed after treatment with combinations of adjuvant with both antibiotics (Figure S13C). The antibiofilm activity was maintained across species, as indicated by a reduction of 4.6 log CFU/mL in the bacterial burden of *P. aeruginosa* biofilms (Figure S14A) and a 1.6 log CFU/mL reduction in MRSA biofilm burden (Figure S14C) upon treatment with fusidic acid + D-LBDiphe (16 + 16 μg/mL). A 6.5 log CFU/mL reduction in the dispersed cell count for *P. aeruginosa* and a 4.8 log CFU/mL reduction for MRSA were observed upon treatment with a combination of fusidic acid and D-LBDiphe (Figure S14B, D).

**Figure 4:**
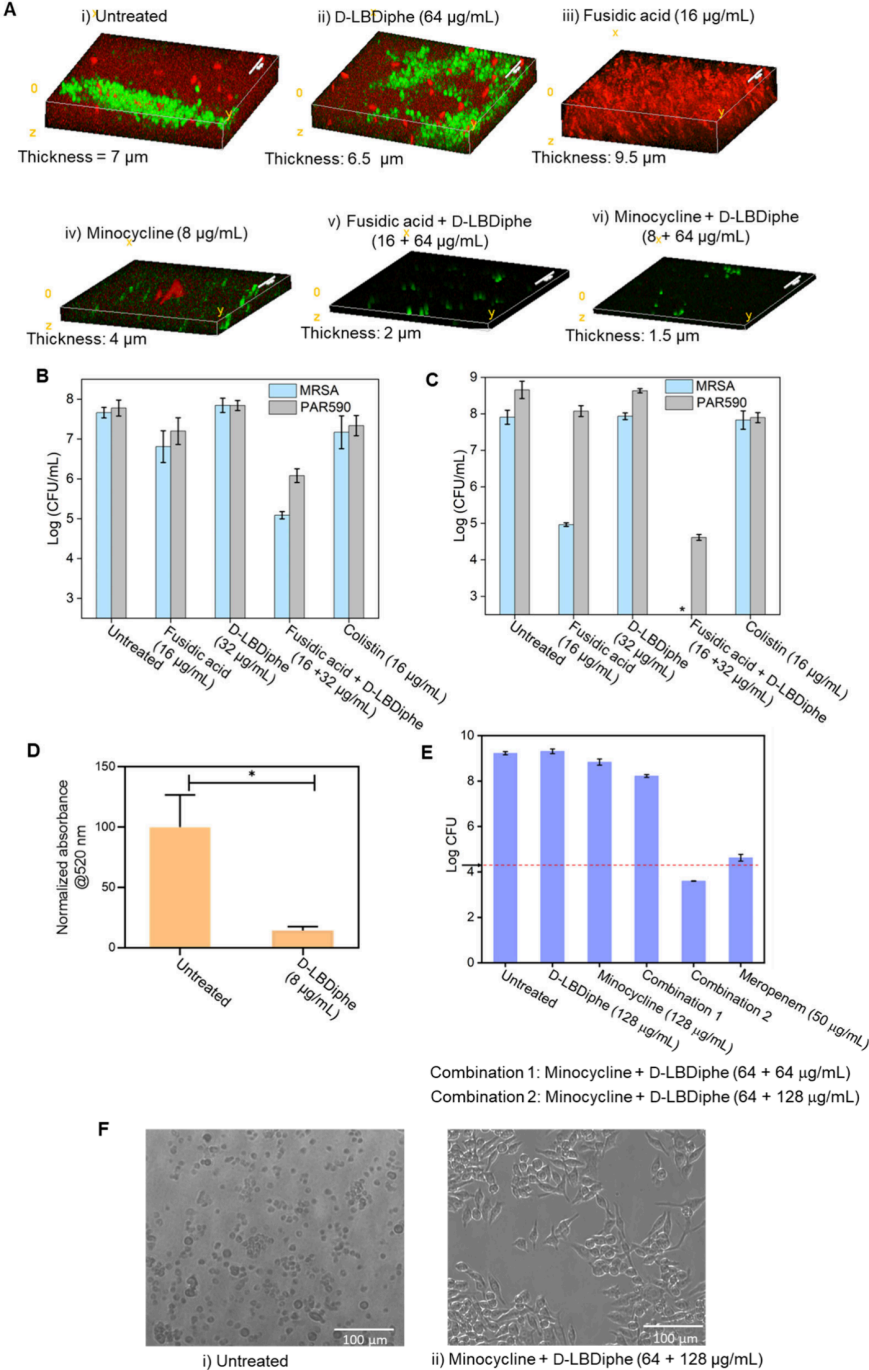
Activity of D-LBDiphe and antibiotic combinations against mixed-species biofilms, intracellular infection, and investigation of antivirulence properties. (A) Confocal laser scanning microscopy images of mixed biofilms of dsred-tagged PAO1 and GFP-tagged SH1000 across different treatment arms. (i) Untreated, (ii) D-LBDiphe (64 μg/mL), (iii) fusidic acid (16 μg/mL), (iv) minocycline (8 μg/mL), (v) fusidic acid + D-LBDiphe (16 + 64 μg/mL), (vi) minocycline + D-LBDiphe (8 + 64 μg/mL). Scale bar in CLSM images is 10 μm. (B) Viability of biofilm-embedded bacteria in mixed biofilm of *P. aeruginosa* R590 and MRSA ATCC 33591 (*N* = 2). (C) Viability of dispersed cells in mixed biofilm of *P. aeruginosa* R590 and MRSA ATCC 33591 (*N* = 2). Asterisk indicates <50 CFU/mL. (D) Assessment of pyocyanin levels in PAO1 after 24 h treatment with D-LBDiphe (*n* = 2). Asterisk indicates *P* = 0.0457 (using Student’s *t* test). (E) Bacterial titer after 24 h of PAO1 infection in RAW macrophages across different treatment arms (*N* = 2). Combination 1 stands for minocycline (64 μg/mL) + D-LBDiphe (64 μg/mL). Combination 2 stands for minocycline (64 μg/mL) + D-LBDiphe (128 μg/mL). (F) Microscopy images of (i) untreated and (ii) minocycline + D-LBDiphe (64 + 128 μg/mL) treated RAW macrophages 24 h after PAO1 infection. Arrow and red dotted line in (E) indicate pretreatment count of bacteria taken 1 h after intracellular infection (MOI = 4). Scale bar in F is 100 μm.

Seeing the potency of combinations against single species biofilms of Gram-positive and Gram-negative bacteria, their efficacy was tested next against mixed species biofilms of MRSA and *P. aeruginosa* (Figure 4A–C). GFP tagged *S. aureus* and dsred tagged *P. aeruginosa* were utilized to form mixed species biofilms. *P. aeruginosa* (PAO1) prevented the colonization of *S. aureus* in the multispecies biofilm, which was evident from confocal microscopy and bacterial count enumeration in the biofilm (Figure 4A-i and Figure S14E). However, D-LBDiphe, which is a nonactive compound, led to an increase in *S. aureus* colonization, as evidenced from confocal microscopy (Figure 4A-ii and a 2.1 log CFU/mL increase in the *S. aureus* biofilm burden (Figure S14E). The combination treatments with both antibiotics led to a 5–5.5 μm reduction in the thickness of the biofilm (Figure 4A).

Against mixed species biofilms of clinical isolates, a 1.5 log CFU/mL reduction was observed in the biofilm viability of *P. aeruginosa*, and a 2.5 log CFU/mL reduction was observed in the viability of MRSA by a combination of fusidic acid (16 μg/mL) and D-LBDiphe (32 μg/mL) (Figure 4B). A 4 log CFU/mL reduction was observed in the viability of dispersed *P. aeruginosa* and an 8 log CFU/mL reduction was observed for dispersed MRSA (Figure 4C). These results validated the efficacy of the combination of D-LBDiphe with antibiotics in eradicating mixed species biofilms of *P. aeruginosa* and MRSA.

The combinations were better than the last-resort antibiotic, colistin, which at 16 μg/mL showed less than 1 log CFU/mL reduction of both bacteria in biofilm burden and dispersed cell count (Figure 4B–C). To gauge the difference in the mixed biofilm composition upon treatment with D-LBDiphe, virulence factors were assessed in *P. aeruginosa* and MRSA. After treatment with D-LBDiphe at 8 μg/mL, an ∼85% reduction in the amount of pyocyanin, an important virulence factor^39,40^ for biofilm formation, was observed (Figure 4D). The reduced virulence of PAO1 might be the reason for increased colonization of *S. aureus* in the presence of D-LBDiphe (Figures 4A-ii and S14E). Only a 1.5% reduction, although significant, was observed in the amount of the *S. aureus* virulence factor staphyloxanthin upon treatment with 16 μg/mL D-LBDiphe (Figure S14G). Together, these studies indicate antivirulence properties of D-LBDiphe against Gram-negative and Gram-positive bacteria. This is a new finding for membrane-perturbing adjuvants, opening the avenue for investigation of their antivirulence and quorum sensing inhibition properties.

### Combination of D-LBDiphe and Minocycline Shows Excellent Efficacy against Intracellular *P. aeruginosa* Infection

Intracellular infections are a cause of concern, as various obligate and facultative pathogens can invade phagocytic and nonphagocytic cells through multiple mechanisms, resulting in bacterial survival.^41^ Intracellular bacteria can release multiple virulence factors, hijack the pathways responsible for pathogen clearance and evade host immune response.^42^ Moreover, bacteria hiding in vacuoles are inaccessible to most antibiotics. Intracellular infection is prevalent in urinary tract, gastric and pulmonary infections,^43^ which needs to be overcome with novel therapies in the pipeline. To understand the effect of D-LBDiphe on the activity of antibiotics to combat intracellular pathogens, we tested the intracellular activity of the combination of minocycline and D-LBDiphe against PAO1 intracellular infection in RAW macrophages. The pretreatment intracellular bacterial count was 4.2 log CFU. In the untreated, minocycline and D-LBDiphe treated cases, the total bacterial count rose to ∼9 log CFU (Figure 4E). In the D-LBDiphe and minocycline combination treated cells at 64 μg/mL of both components (Combination 1), a 1 log CFU reduction in bacterial burden was observed as compared to the untreated control. At the higher concentration of 64 μg/mL minocycline and 128 μg/mL D-LBDiphe (Combination 2), there was a ∼0.6 log CFU reduction with respect to the pretreatment load, which signifies a 75% reduction in the intracellular burden and a 5.6 log CFU reduction as compared to the bacterial burden in the untreated control at 24 h (Figure 4E). Meropenem, which is a cell permeable antibiotic, led to a 0.4 log CFU increase in the bacterial burden with respect to the pretreatment count. The viability of the cells 24 h after infection was also confirmed through microscopy, where the untreated cells showed debris due to lysis, whereas the morphology of cells were intact in the combination 2 treated case, indicating rescue from intracellular pathogenesis (Figure 4F). . D-LBDiphe might be able to make the antibiotic more accessible to the intracellular bacteria inside cells, making the combination effective against intracellular *P. aeruginosa* infection and rescuing the cells from lysis.

### D-LBDiphe Interacts with Bacterial Lipopolysaccharide and Exhibits Endotoxin-Responsive Immunomodulatory Properties in Mice

During severe infections, antigens like lipopolysaccharide (LPS), lipoteichoic acid (LTA) and peptidoglycan can trigger an unwanted immune response by binding to toll-like receptors (TLR) on immune cells, often leading to hyperinflammation and septic shock.^44,45^ This immune response can be further exacerbated due to bacterial lysis upon antibiotic therapy and thus needs to be managed alongside bacterial infection. Targeting these antigens can be an innovative strategy to block or decline the immune cascade that happens downstream of antigen-binding to immune cells. D-LBDiphe, because of its structural features, has the capability to interact with the endotoxin LPS through noncovalent interactions. These interactions can alter the self-assembly of LPS, changing the nature of the LPS aggregates.

**Figure 5:**
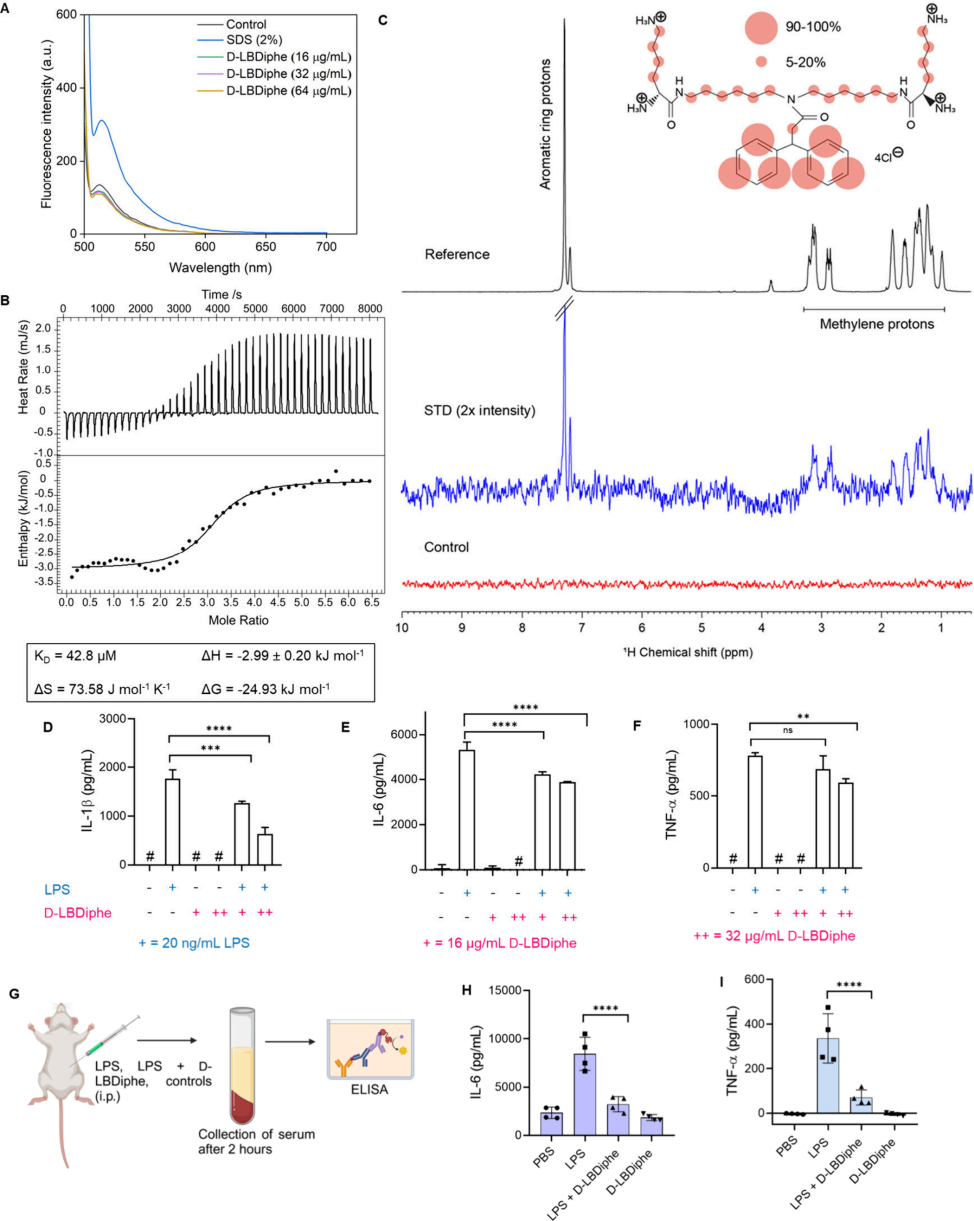
Investigation of interaction of D-LBDiphe with aggregates of lipopolysaccharide and assessment of immunomodulatory potential. (A) Fluorescence intensity of BODIPY-LPS in the presence of varying concentrations of D-LBDiphe and SDS. (B) Isothermal titration calorimetry of D-LBDiphe in the presence of LPS and calculated thermodynamic parameters. Upper panels depict exothermic heat of reaction vs time (seconds) upon interaction of LPS with D-LBDiphe. Lower panels depict enthalpy change per mole of LPS injection vs molar ratio (LPS: D-LBDiphe). (C) 1D STD NMR analysis of free (control, lower panel) and bound (STD, middle) D-LBDiphe compound in the presence of *E. coli* 0111: B4 LPS micelle. The reference ^1^H spectrum in the top panel shows the aromatic and methylene protons at pH 4.5 in 100% D_2_O at 298 K. Production of IL-1β (D), IL-6 (E) and TNF-α (F) after stimulation of hPBMCs with LPS (20 ng/mL) either in the presence or absence of D-LBDiphe at 16 and 32 μg/mL. D-LBDiphe alone was used as control. 1X-PBS was used as negative control (*n* = 3 for D-F). (G) Schematic for the assessment of *in vivo* immunomodulation. The *in vivo* levels of cytokines IL-6 (H) and TNF-α (I) in the sera of BALB/c mice were analyzed 2 h after the i.p. injection of LPS (0.1 mg/kg), D-LBDiphe (30 mg/kg) or LPS + D-LBDiphe (0.1 mg/kg + 30 mg/kg; LPS and D-LBDiphe were preincubated for ∼20 min before i.p. injection). 1X-PBS was injected as vehicle control (*n* = 4 for H and I). Statistical analysis was carried out using One-way ANOVA and Student’s *t* test. “****” indicates *P* < 0.0001, “***” indicates *P* < 0.001 ‘**’ indicates *P* < 0.01, “ns” indicates not significant. “#” indicates values below the quantification limit.

It is known that the initial binding of LPS to LPS-binding protein (LBP) before its transfer to the cluster of differentiation factor 14 (CD14) happens with aggregated LPS.^24^ In view of this, to understand the interaction of D-LBDiphe with LPS, various biophysical and microbiological investigations were carried out and finally the effect of D-LBDiphe on reducing the levels of pro-inflammatory cytokines like interleukins (IL-1β and IL-6) and tumor necrosis factor (TNF-α) upon stimulation of human peripheral blood mononuclear cells (PBMCs) with LPS was determined. Dynamic light scattering studies were performed to understand the effect of adjuvant treatment on LPS aggregates with time. The order of distribution of LPS aggregates changed from bimodal to almost unimodal, indicating a shift of lower order aggregates to higher order aggregates after adjuvant treatment at 25 μg/mL (Figure S15C–D). The mean count rate (kcps) increased after treatment of LPS with adjuvant in a concentration-dependent manner, indicating formation of more stable aggregates (Figure S15E). This was further understood by fluorescence studies with BODIPY-conjugated-LPS. The fluorescence of BODIPY in BODIPY-LPS conjugate is self-quenched due to the aggregating nature of LPS. A surfactant like sodium dodecyl sulfate (SDS) disrupts the LPS aggregates, leading to an increase in the fluorescence intensity of BODIPY. Upon treatment with D-LBDiphe, a decrease in the fluorescence intensity was observed in a concentration-dependent manner (Figure 5A). This was similar to the effect of colistin on LPS aggregates. This indicates that D-LBDiphe caused the conversion of lower order LPS aggregates to higher order aggregates by promoting aggregation, different from the surfactant SDS. This can be a result of the amine groups of D-LBDiphe interacting with LPS through hydrogen bonding and electrostatic interactions as well as the phenyl rings interacting with the acyl chains of LPS.

Thermodynamical parameters guiding the binding interaction of D-LBDiphe and LPS were determined through isothermal calorimetry experiments, as shown in Figure 5B. The negative value of Δ*G* (−24.93 kJ mol^–1^) showed that the interaction was spontaneous and thermodynamically driven with positive Δ*S* (73.58 J mol^–1^ K^–1^) and negative Δ*H* (−2.99 kJ mol^–1^) values. The interaction was driven more by the entropic component; however, it was also enthalpically favored. The thermodynamically driven process can be explained by hydrogen bonding and electrostatic interactions of D-LBDiphe with the phosphate groups of LPS, and the high entropic component might be due to solvated water released after increased aggregation due to hydrophobic interactions. This corroborates with the increased aggregation observed in the DLS and fluorescence experiments as well. The dissociation constant (K_D_) was 42.8 μM, indicating good binding interaction with the LPS aggregates.

To gain a molecular understanding of this interaction at an atomic resolution, STD NMR was performed to define the epitope of D-LBDiphe, binding to LPS micelle. Careful analysis of the data suggested that D-LBDiphe strongly interacts with LPS micelle as evidenced by strong STD peaks of the aromatic ring proton groups along with comparatively low signals from methylene (-CH_2_) protons (Figure 5C, middle panel). The group epitope mapping of the individual protons indicated that only the two aromatic rings present in D-LBDiphe are in close vicinity of the acyl chains of the LPS micelle, as shown by the highest peak intensity in the STD spectrum (90–100%). On the contrary, methylene protons of the backbone and lysine-methylene protons showed very weak STD signals (5–20%), which indicates the remoteness of the methylene protons from LPS aggregates. This study revealed the role of the aromatic moiety in the interaction with the LPS aggregates. We also suspect the interaction of the amine groups with the phosphate groups of LPS via electrostatic and hydrogen bonding interactions. Since these protons are readily exchanged in D_2_O, these interactions could not be mapped through 1D-STD NMR studies.

After obtaining a thermodynamic and molecular understanding of the interactions between LPS and D-LBDiphe, we investigated the immunomodulatory effect of D-LBDiphe. We presume that the interaction of LPS with D-LBDiphe leading to a change in the native nature of LPS aggregates would hinder LPS recognition by LBP and dampen subsequent transfer of LPS molecule to CD14 and TLR-4. This would lead to reduction in activation of various pro-inflammatory cascades^46^ and subsequent production of pro-inflammatory cytokines. To investigate the immunomodulatory potential of D-LBDiphe, the levels of pro-inflammatory cytokines like IL-6, IL-1β and TNF-α in human PBMCs were assessed by ELISA. There was a significant increase in the levels of all cytokines upon induction with LPS (Figure 5D–F). D-LBDiphe did not induce any cytokine expression, as the levels were similar to that of the untreated control. When D-LBDiphe was coadministered with LPS, there was a significant reduction in the amount of all cytokines. IL-1β amount was reduced by 28% upon treatment with D-LBDiphe at 16 μg/mL and by 64% upon treatment with D-LBDiphe at 32 μg/mL, showing a concentration-dependent effect (Figure 5D). The amount of IL-6 was reduced by 21% at 16 μg/mL D-LBDiphe and by 28% at the higher concentration (Figure 5E). Similarly, D-LBDiphe reduced the level of TNF-α by 13% at the lower concentration and by 25% at the higher concentration (Figure 5F). The immunomodulatory effects of D-LBDiphe were also observed *in vivo*. Briefly, mice were intraperitoneally injected with PBS, LPS, LPS which was preincubated with D-LBDiphe for 20 min, and D-LBDiphe alone (Figure 5G). After 2 h of injection, blood was collected via retroorbital sinus, serum was isolated, and ELISA was performed to assess levels of pro-inflammatory cytokines. When BALB/c mice were injected with LPS (0.1 mg/kg) and D-LBDiphe (30 mg/kg), preincubated for about 20 min before intraperitoneal injection, there was a 61.8% reduction in the level of IL-6 and a 79% reduction in the level of TNF-α as compared to only LPS injection at 0.1 mg/kg (Figures 5H–I). The levels of cytokines were comparable to those of the mice injected with vehicle control. These results suggest that D-LBDiphe has the potential to act as an LPS-responsive immunomodulatory agent by reducing the levels of pro-inflammatory cytokines. Consistent immune activation by LPS can be detrimental to patients even after the infection has considerably subsided. An adjuvant possessing dual-functional nature of “antibiotic potentiation” as well as “immunomodulation” can be used as an effective and wholesome therapeutic strategy to combat infection and infection-associated inflammation.

### D-LBDiphe Shows *In Vivo* Biocompatibility and Repurposes Antibiotics against *P. aeruginosa* Infections

**Figure 6:**
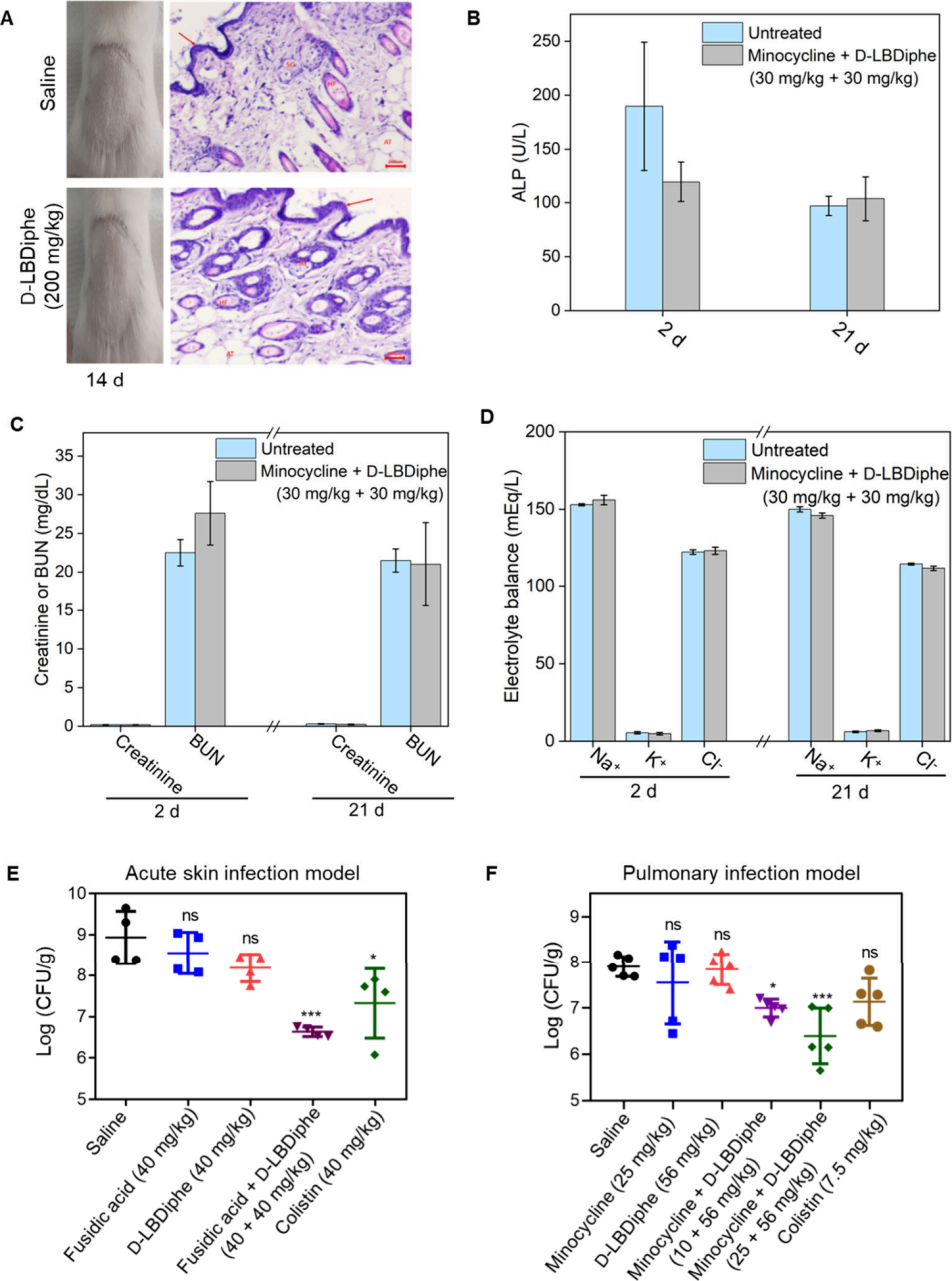
*In-vivo* toxicity and efficacy of D-LBDiphe as an adjuvant. (A) Dermal toxicity of D-LBDiphe through visual observation and hematoxylin and eosin staining (scale bar is 200 μm). (B) Liver function as a measure of alkaline phosphatase (ALP) after combination dose of minocycline and D-LBDiphe via intraperitoneal administration (*n* = 5). (C) Kidney function as a measure of blood urea nitrogen (BUN) and creatinine after combination dose of minocycline and D-LBDiphe via intraperitoneal administration (*n* = 5). (D) Electrolyte balance after combination dose of minocycline and D-LBDiphe via intraperitoneal administration (*n* = 5). (E) *In-vivo* efficacy against skin infection of *P. aeruginosa* MTCC 424 (*n* = 4/treatment arm). (F) *In-vivo* efficacy against pulmonary infection of *P. aeruginosa* PAO1 (*n* = 5/treatment arm). All treatments in (F) except colistin were administered intratracheally. Colistin was administered through an intravenous injection. Statistical analysis was performed using One-way ANOVA. “***” indicates *P* < 0.001, “*” indicates *P* < 0.05, “ns” indicates not significant.

After streamlined investigation of multifaceted properties of D-LBDiphe, the *in vivo* biocompatibility and efficacy were determined to deliver a lead combination as a preclinical therapeutic candidate. D-LBDiphe was biocompatible dermally at 200 mg/kg, which was also confirmed through hematoxylin-eosin staining (Figure 6A). The lethal dose of D-LBDiphe to kill 50% of mice (LD_50_) was >175 mg/kg via the subcutaneous route.

The maximum tolerated dose of the combination therapy of minocycline and D-LBDiphe was 30 mg/kg minocycline + 30 mg/kg D-LBDiphe via the intraperitoneal route.

The subchronic toxicity of the combination at this dose was determined by doing blood biochemistry (Figure 6B–D). After 2 and 21 days, the liver and kidney function (measured by levels of corresponding biomarkers such as alkaline phosphatase (ALP), blood urea nitrogen (BUN), and creatinine) and electrolyte balance were similar to those of the untreated mice. Furthermore, the histopathological changes in the liver and kidney of the combination-treated mice were evaluated 2 days post-treatment using hematoxylin and eosin staining. The analysis showed no noticeable changes in the organs of the treated mice as compared to the untreated control (Figure S17).

Kidney sections in both the cases showed normal appearance with glomerulus containing tuft of capillaries surrounded by Bowman’s capsule and the proximal convoluted tubules lined by columnar epithelial cells. Mild infiltration of interstitial spaces by red blood cells was observed in both the untreated and treated cases. The liver sections showed normal architecture of the hepatocytes around the central vein, and sinusoidal spaces showed the presence of Kupffer cells. These investigations suggested that treatment with a combination of D-LBDiphe and minocycline did not cause any significant hepatotoxicity or renal toxicity. The biocompatible nature of the adjuvant prompted us to investigate it further in an *in vivo* infection model.

As fusidic acid is a prescribed topical treatment for infections caused by Gram-positive bacteria, we wanted to see whether fusidic acid can be repurposed to treat topical infections caused by Gram-negative bacteria. The *in vivo* antibacterial efficacy of the combination was tested against murine acute skin infection of *P. aeruginosa* (Figure 6E). The individual topical treatment of fusidic acid and D-LBDiphe at 40 mg/kg showed bacterial burden similar to that of the vehicle control. The combined treatment of fusidic acid and D-LBDiphe (40 + 40 mg/kg) showed a ∼2.5 log CFU/g (99.5%) reduction in bacterial burden, which was even better than the last resort antibiotic, colistin, at 40 mg/kg (1.6 log CFU/g reduction) (Figure 6E).

Moreover, to validate the potential of D-LBDiphe in the treatment of systemic infections, its efficacy was tested against pulmonary infection of *P. aeruginosa* established in mice. A combination of D-LBDiphe and minocycline was tolerated by mice at a dose of 56 mg/kg each, administered intratracheally. The intratracheal route of administration was chosen for treatment against pulmonary infection. Individual intratracheal treatments of D-LBDiphe (56 mg/kg) and minocycline (25 mg/kg) displayed bacterial burden similar to the vehicle control (Figure 6F). A combined treatment of minocycline and D-LBDiphe (10 + 56 mg/kg) displayed a significant 0.9 log CFU/g reduction in the lung bacterial burden, whereas a higher dose treatment of minocycline and D-LBDiphe (25 + 56 mg/kg) exhibited a 1.5 log CFU/g reduction in lung bacterial burden (Figure 6F). As a positive control, intravenous treatment of colistin (7.5 mg/kg) was given which, resulted in a 0.77 log CFU/g reduction in bacterial burden; however, it was not significant. This validates the superior efficacy of the adjuvant and antibiotic treatment against systemic pulmonary infection of *P. aeruginosa*.

These experiments validate D-LBDiphe as a potent candidate to repurpose antibiotics against topical and systemic infections caused by Gram-negative superbugs. Here, intratracheal administration of an antibiotic and adjuvant proved to be promising against pulmonary infection. The pharmacokinetics and pharmacodynamics of the combination therapy need to be investigated in the future to explore other routes of administration. These studies pave the way for assessment of the therapeutic potential of D-LBDiphe as an antibiotic adjuvant against other systemic infections.

## Conclusions

Antibiotic resistance and phenotypic forms of antibiotic tolerance like intracellular infection, mixed species biofilms, persisters, and stationary phase bacteria, along with the threat of hyperinflammation, make the treatment of Gram-negative bacterial infections challenging. Thorough investigations in this work have yielded a dual-functional antibiotic adjuvant, D-LBDiphe, which not only targets antibiotic resistance and antibiotic tolerance in Gram-negative bacteria but also shows immunomodulatory properties to combat infection-associated inflammation. The mechanistic properties of D-LBDiphe were investigated in detail with insights into the role of structural motifs in governing a majorly Coulombic attraction driven interaction with bacterial membrane lipids. Aromatic rings in the design were instrumental in its interaction with LPS, which led to reduction of pro-inflammatory cytokines in a mice model mimicking infection-associated inflammation. D-LBDiphe repurposed two different antibiotics against topical and pulmonary infection of *P. aeruginosa* in mice. This work presents D-LBDiphe as the first multifaceted antibiotic adjuvant to treat complicated Gram-negative bacterial infections and infection associated inflammation and paves the way for its preclinical development. Insights into the mechanistic properties of D-LBDiphe through microbiological, biophysical and computational studies provide a better understanding into the role of chemical design to assist in the discovery of such multifaceted compounds, and *in vivo* studies validate its potential as a preclinical candidate, while also paving the way for detailed investigations in the future.

## Supporting Information Available

Supplementary tables and figures, materials and methods for *in vitro* biological evaluation; and *in vivo* toxicity and activity, detailed synthesis procedures, and characterization of intermediates and final compounds (^1^H NMR, ^13^C NMR, HR-MS). (PDF)
