# Supplementary material for: Dual-Functional Antibiotic Adjuvant Displays Potency against Complicated Gram-Negative Bacterial Infections and Exhibits Immunomodulatory Properties
Source: ACS Cent Sci. 2025 Jan 17;11(2):279–93. doi: 10.1021/acscentsci.4c02060 (PMC11868958; doi:10.1021/acscentsci.4c02060)
Supplement: Supplementary file 1 — oc4c02060_si_001.pdf [file oc4c02060_si_001.pdf]

Supporting information for:

Dual-functional antibiotic adjuvant displays potency against complicated Gram-negative bacterial infections and exhibits immunomodulatory properties

Geetika Dhanda[1], Himani Singh[1], Abhinav Gupta[2], Sk Abdul Mohid[3], Karishma Biswas[3], Riya Mukherjee[1], Smriti Mukherjee[1], Anirban Bhunia[3], Nisanth N. Nair[2], Jayanta Haldar[1,4*]

[1]Antimicrobial Research Laboratory, New Chemistry Unit, Jawaharlal Nehru Centre for Advanced Scientific Research, Jakkur, Bengaluru 560064, Karnataka, India

[2]Department of Chemistry, Indian Institute of Technology Kanpur, Kanpur 208016, India

[3]Department of Chemical Sciences, Bose Institute, Kolkata 700091, India

[4]School of Advanced Materials, Jawaharlal Nehru Centre for Advanced Scientific Research, Jakkur, Bengaluru 560064, Karnataka, India

*Corresponding author: Jayanta Haldar, Email ID: jayanta@jncasr.ac.in, Ph. No.: +91 802208 2565

Table of Contents

Supporting information figures, tables and schemes S2-S24
Experimental section (materials, synthesis and characterization) S24-S39
Experimental section (biological assays and simulations) S39-S58
References S58-S61

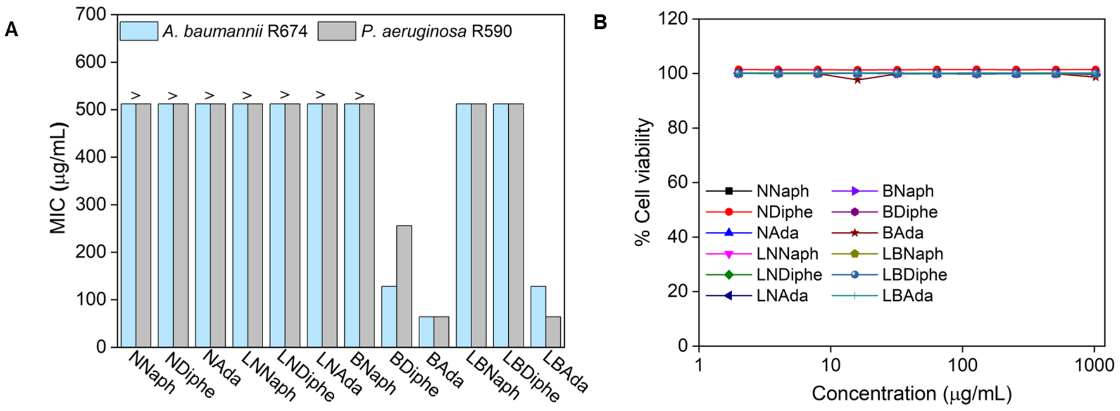

Figure S1. Structure-activity and toxicity studies (A) Activity of compounds against *A. baumannii* and *P. aeruginosa*. (B) Toxicity profile of compounds against human erythrocytes (n =3).

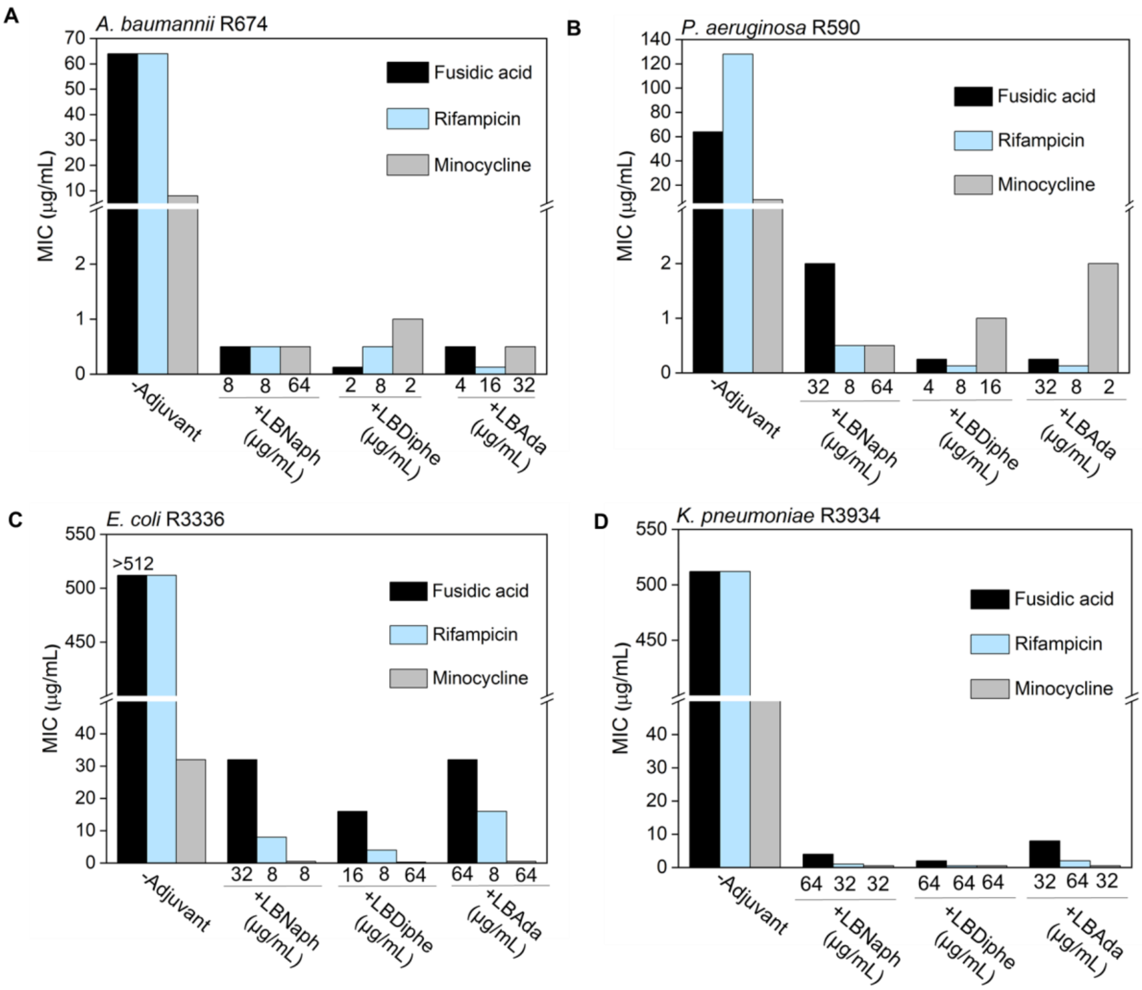

Figure S2. Potentiation activity of optimized adjuvants against clinical isolates. MIC of fusidic acid, rifampicin and minocycline in presence and absence of optimized adjuvants against (A) *A. baumannii* R674 (B) *P. aeruginosa* R590 (C) *E. coli* R3336 (D) *K. pneumoniae* R3934.

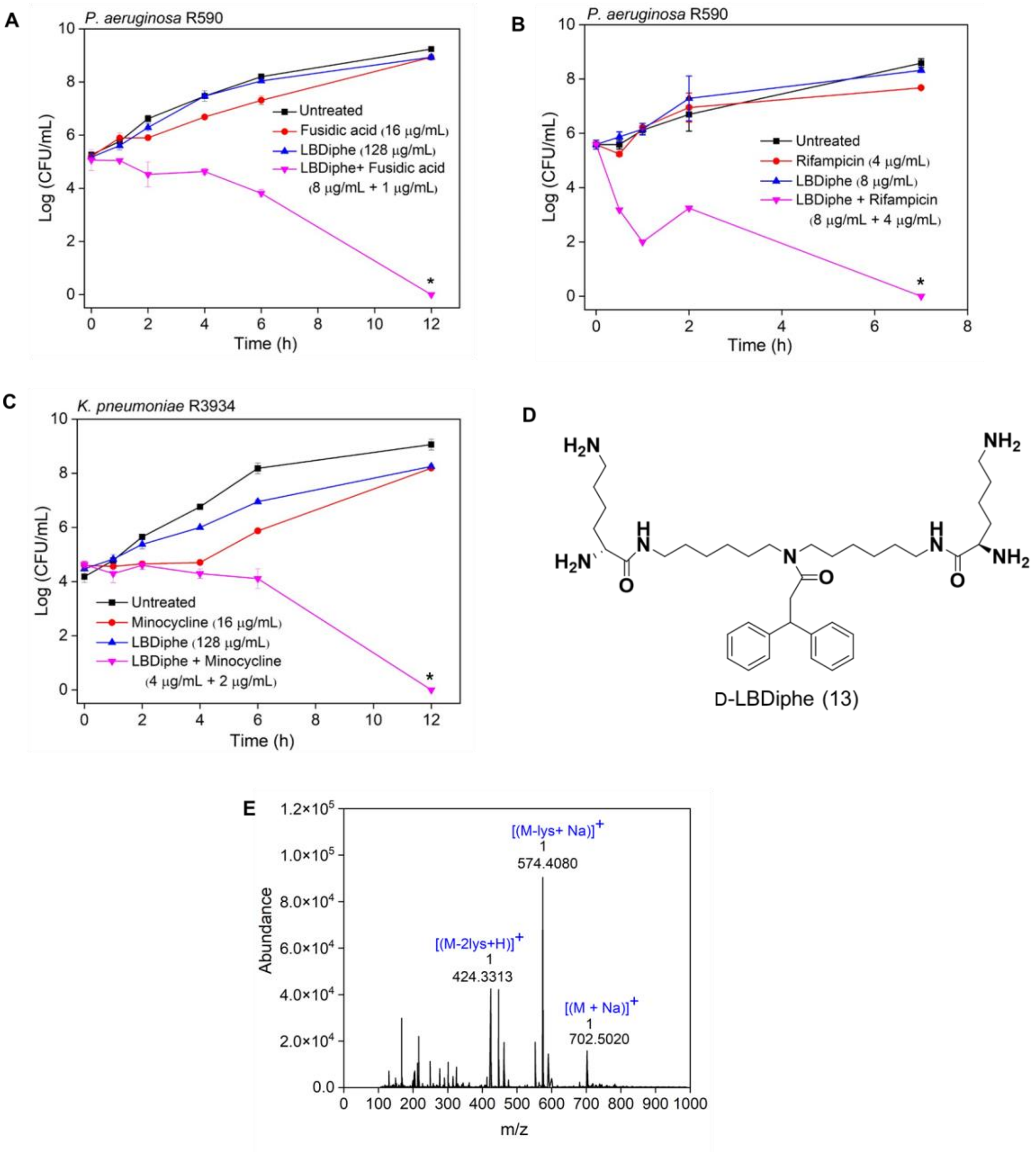

Figure S3. Bactericidal kinetics of LBDiphe and stability in physiological fluids. Bactericidal kinetics of combination of (A) LBDiphe and fusidic acid against PA R590 (N = 2) (B) LBDiphe and rifampicin against PA R590 (N = 2) (C) LBDiphe and minocycline against NDM-positive KP R3934 (N = 2). NDM stands for New-Delhi metallo-β-lactamase. '*' indicates < 50 CFU/mL. (D) Structure of D-LBDiphe (E) HRMS spectrum of the supernatant after incubation of LBDiphe with liver homogenate (LH) of mice.

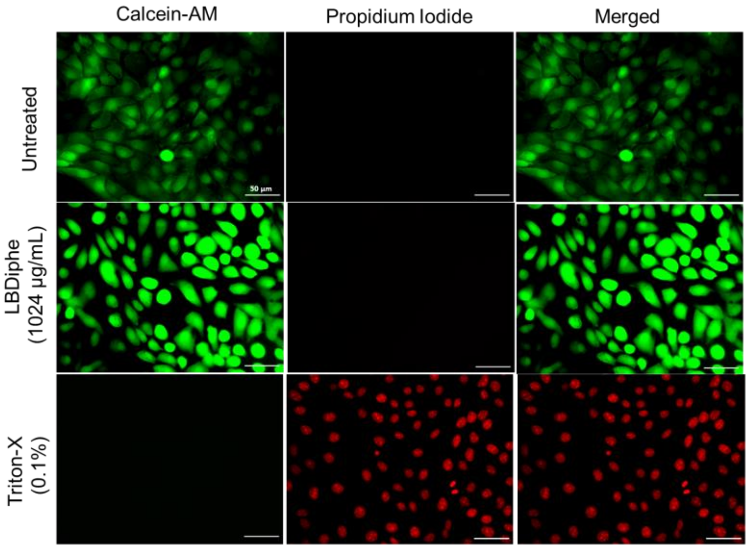

Figure S4. Biocompatibility of LBDiphe towards MDCK cell lines through fluorescence microscopy. Scale bar is 50 µm in all images.

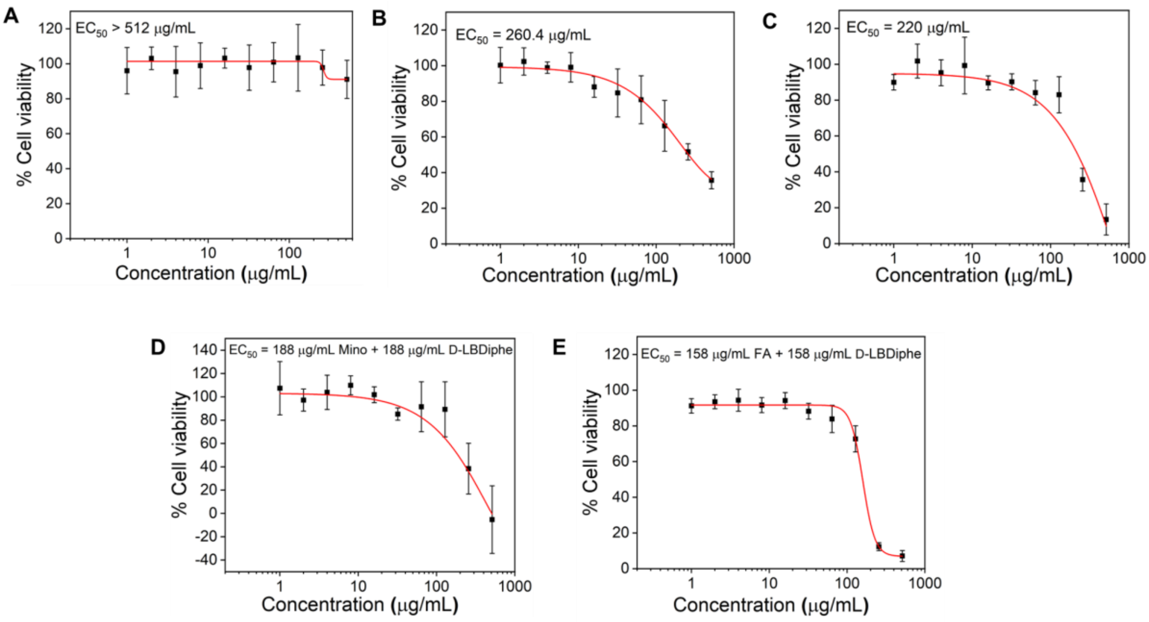

Figure S5. Cytotoxicity of D-LBDiphe and combinations towards HEK 293 cell lines (A) % Cell viability of HEK cells at varying concentrations of D-LBDiphe (B) % Cell viability of HEK cells at varying concentrations of minocycline (C) % Cell viability of HEK cells at varying concentrations of fusidic acid (D) % Cell viability of HEK cells at varying concentrations of minocycline and D-LBDiphe combination (E) % Cell viability of HEK cells at varying concentrations of fusidic acid and D-LBDiphe combination. FA stands for fusidic acid and Mino stands for minocycline. (n = 3 for A-E)

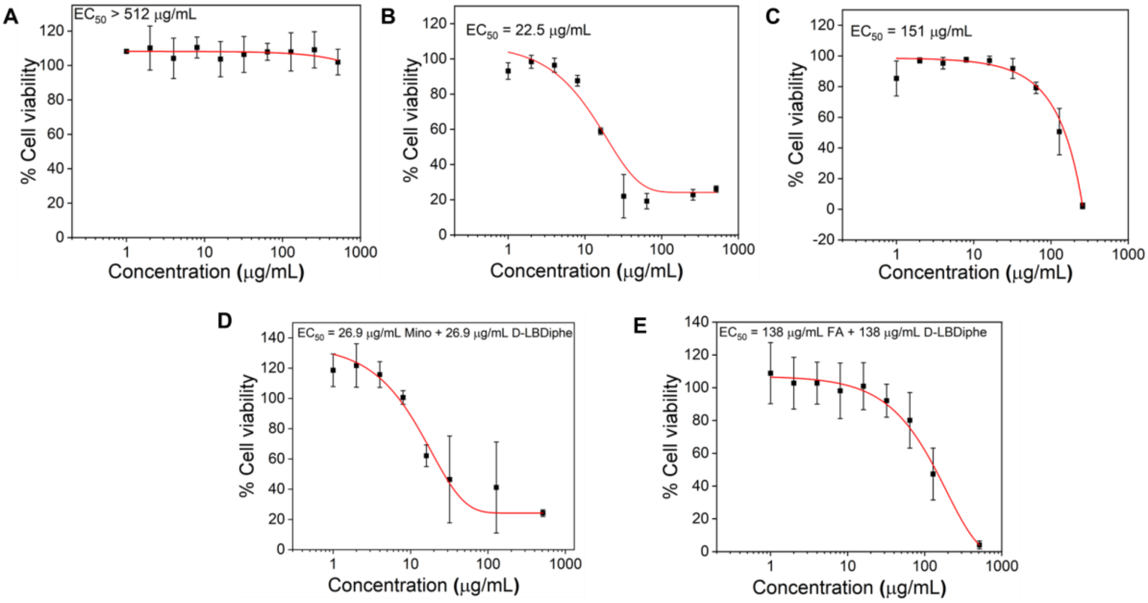

Figure S6. Cytotoxicity of D-LBDiphe and combinations towards human PBMCs (hPBMCs) (A) % Cell viability of hPBMCs at varying concentrations of D-LBDiphe (B) % Cell viability of hPBMCs at varying concentrations of minocycline (C) % Cell viability of hPBMCs at varying concentrations of fusidic acid (D) % Cell viability of hPBMCs at varying concentrations of minocycline and D-LBDiphe combination (E) % Cell viability of hPBMCs at varying concentrations of fusidic acid and D-LBDiphe combination. FA stands for fusidic acid and Mino stands for minocycline. (n = 3 for A-E).

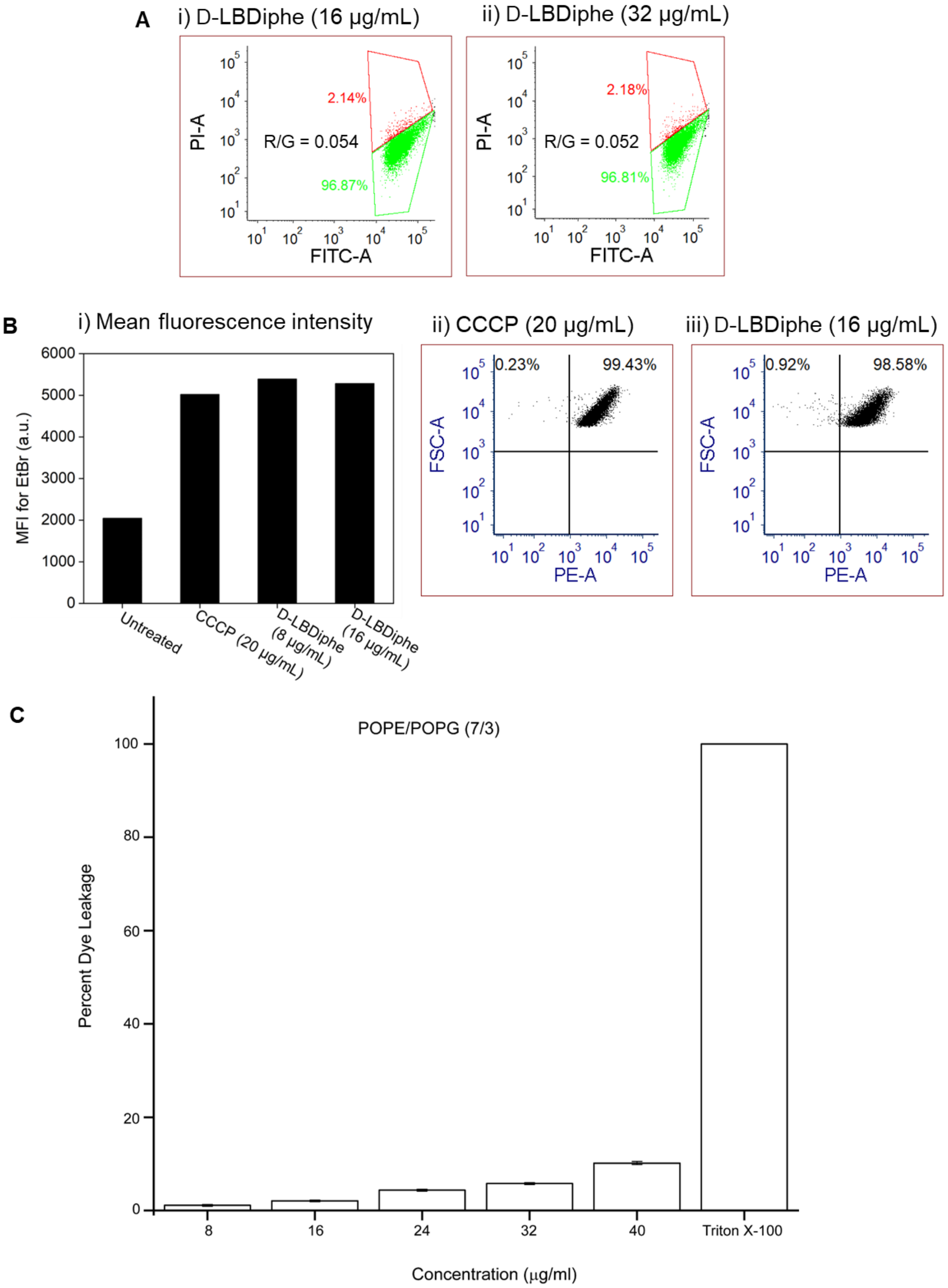

Figure S7. Mechanism of action D-LBDiphe (A) Assessment of membrane potential in PA R590 i) Upon treatment with D-LBDiphe (16 µg/mL) ii) Upon treatment with D-LBDiphe (32 µg/mL). R/G represents the ratio of the mean intensities of the red aggregates versus the green monomer. PI-A stands for propidium iodide-area to detect red fluorescence and FITC-A stands for fluorescein isothiocyanate-area for detecting green fluorescence. (B) Inhibition of efflux machinery in *P. aeruginosa* R590 by D-LBDiphe using flow cytometric analysis i) Mean fluorescence intensity (MFI) for EtBr in cells with different treatments. Dot plot to show EtBr-positive and EtBr-negative cells ii) upon CCCP treatment iii) upon D-LBDiphe treatment at 16 µg/mL. PE-A stands for R-phycoerythrin-area channel for EtBr. FSC-A stands for forward scatter-area. (C) Calcein release in the presence of varying concentrations of D-LBDiphe upon interaction with bacterial model membrane liposome mimic.

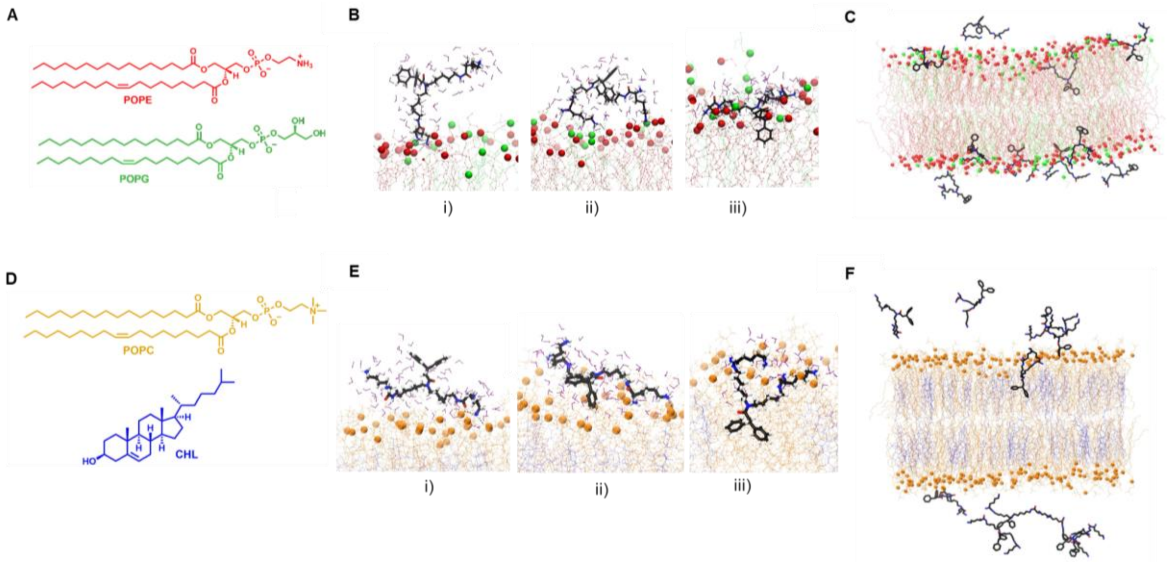

Figure S8. (A) The structure of POPE and POPG lipids used in constructing gram-negative membrane; (B) Three representative snapshots for one interacting D-LBDiphe and gram-negative membrane (Representations/colours: D-LBDiphe (stick; O: red, N: blue, C: black, H: white), lipids (lines; red for POPE and green for POPG), lipid-phosphate head groups (spheres; red for POPE and green for POPG) and water molecules (ball-stick in violet), and (C) Snapshot of the whole system at t = 1000 ns (water molecules are not shown for clarity) from the simulation of gram-negative membrane with ten D-LBDiphe molecules; (D) The structure of constituent lipids, POPC and CHL, used in constructing mammalian membrane; (E) Three representative snapshots for one interacting D-LBDiphe and mammalian membrane (Representations/colours: lipids (lines; orange for POPC and blue for CHL), lipid-phosphate head groups (spheres; orange for POPC), and (F) Snapshot of the whole system at t = 1000 ns (water molecules are not shown for clarity) from the simulation of mammalian membrane with ten D-LBDiphe molecules.

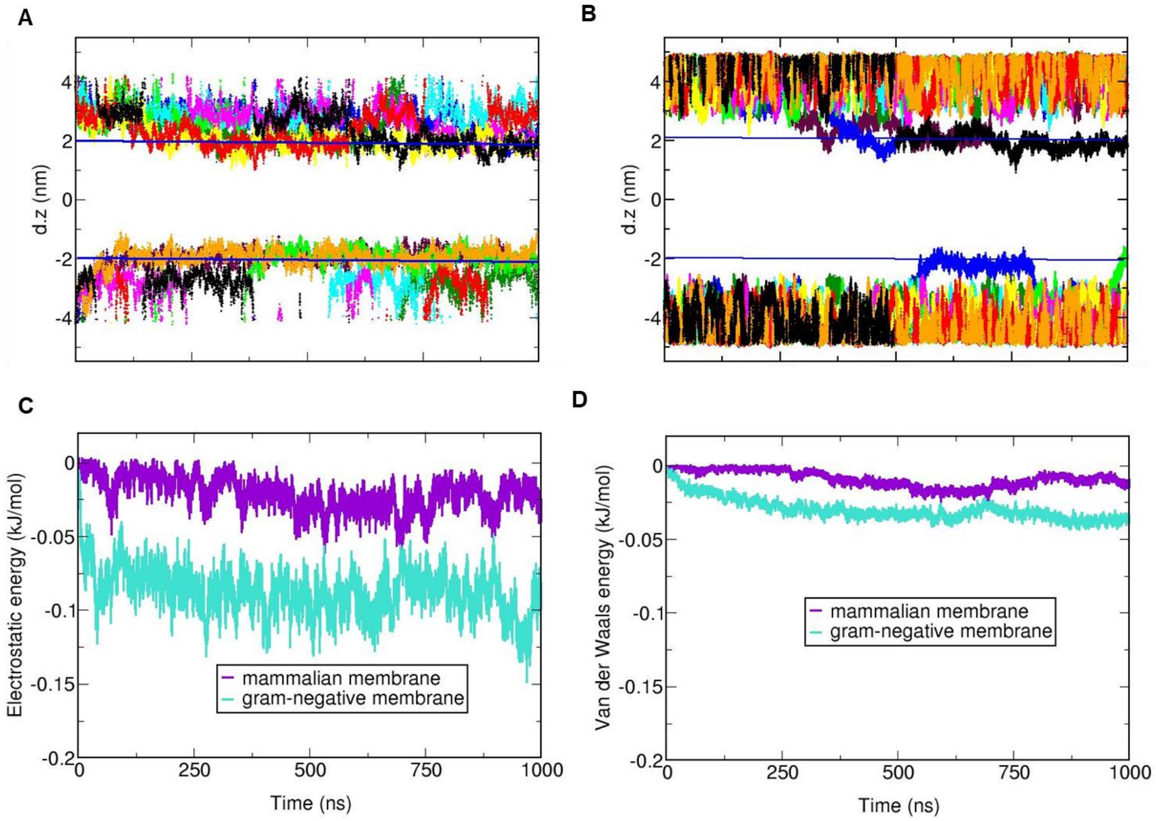

Figure S9. The z-component of distance-vector (d.z) between the centre of mass (COM) of each D-LBDiphe molecule and COM of all the C_{18} of the lipid molecules is plotted for (A) gram-negative, and (B) mammalian membrane during the MD simulations. Ten colours are used for ten D-LBDiphe molecules. The horizontal blue lines on the plot represent the top and bottom leaflet boundaries of the lipid bilayer. The (C) electrostatic and (D) van der Waals energy contribution towards the interaction energy of D-LBDiphe molecules and membrane lipids is plotted for gram-negative (cyan) and mammalian (violet) membrane.

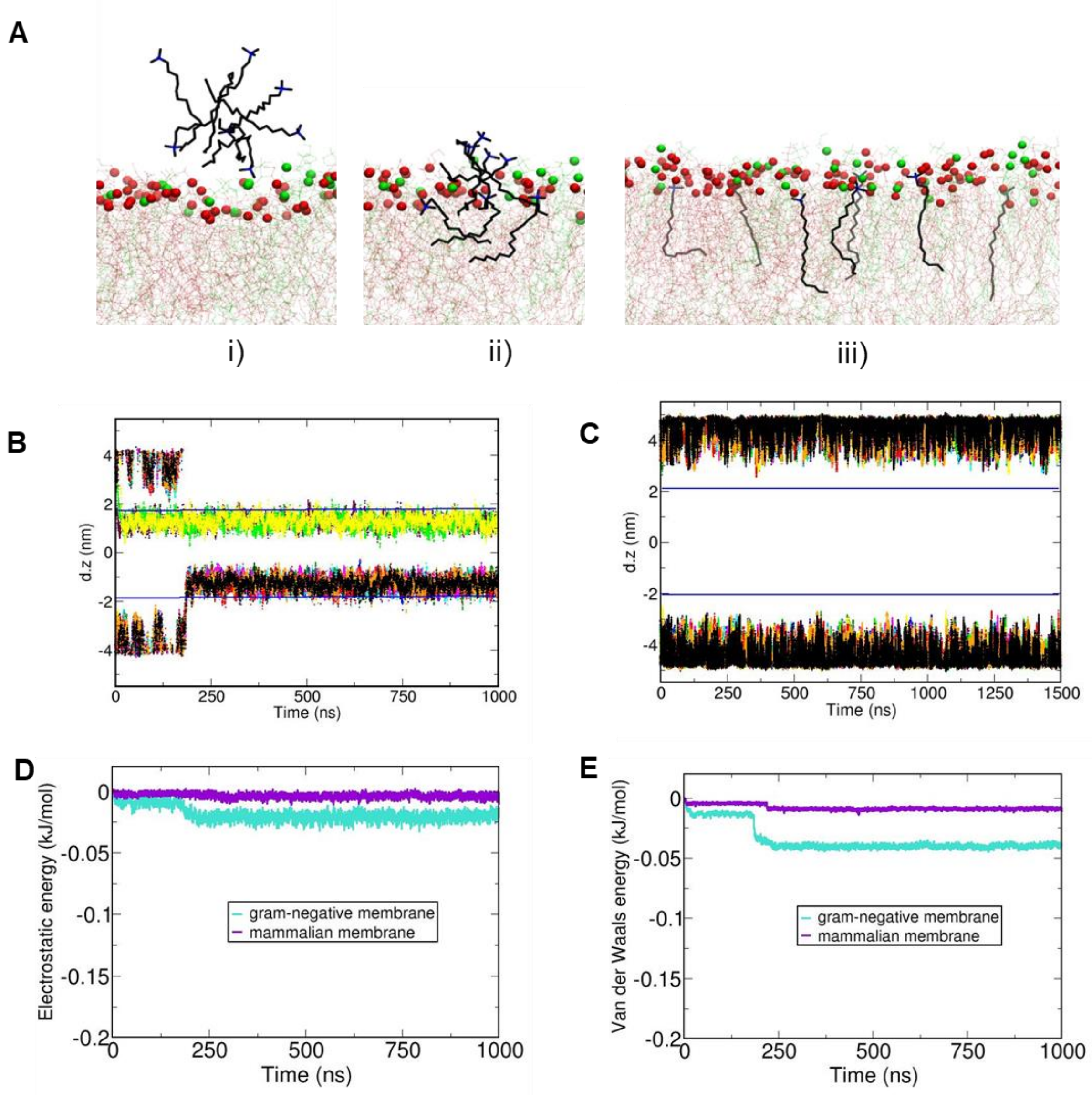

Figure S10. (A) Three representative snapshots from the simulation of gram-negative membrane and ten CTAB molecules. The z-component of distance-vector (d.z) between the COM of each CTAB molecule and COM of all the C_{18} of the lipid molecules is plotted for (B) gram-negative, and (C) mammalian membrane. Ten colours are used for ten CTAB molecules. The horizontal blue lines on the plot represent the top and bottom leaflet boundaries of lipid bilayer. The (D) electrostatic and (E) van der Waals energy contribution towards the interaction energy of CTAB molecules and membrane lipids is plotted for gram-negative (cyan) and mammalian (violet) membrane.

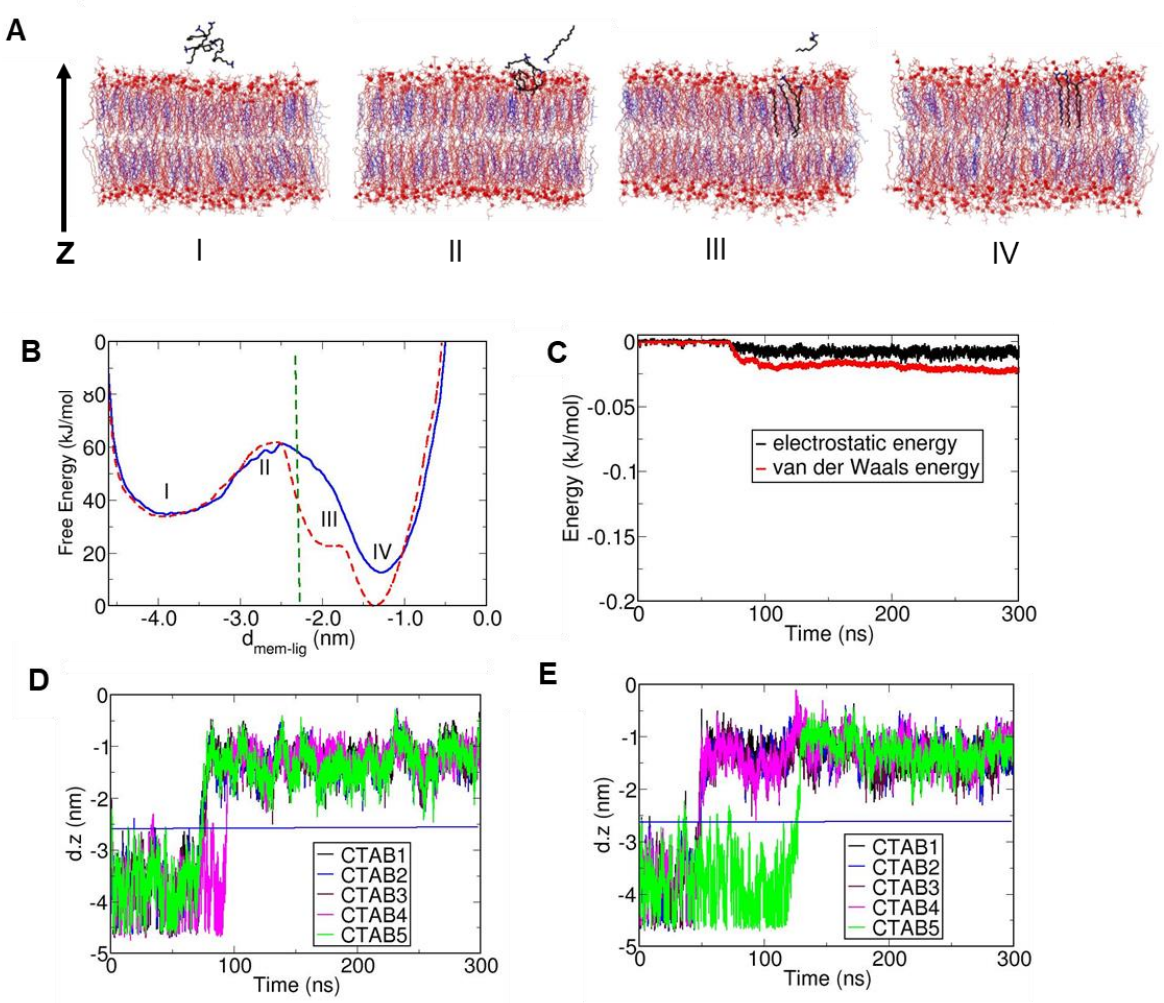

Figure S11. Well-tempered metadynamics simulations for the system containing five CTAB molecules and mammalian membrane; (A) Snapshots of four conformational states; I, II, III and IV of the system. POPC (red lines), CHL (blue lines). (B) The free energy as a function of $d_{mem\text{-}lig}$ is shown for two independent well-tempered metadynamics simulations (solid and dotted lines). Here $d_{mem\text{-}lig}$ is the z-component distance vector between the COM of all the C_{18} atoms (terminal atoms) of lipids to the COM of CTAB molecules (heavy atoms only). (C) The electrostatic and van der Waals energy contribution towards interaction between CTAB and lipids as a function of simulation time is plotted. The z-component of distance vector between COM of every CTAB molecule and COM of the membrane (d.z) is plotted for the two well-tempered metadynamics simulations (D) and (E). Five colours are used for five CTAB molecules. The horizontal blue lines on the plots represent the lipid leaflet boundary of the lower leaflet.

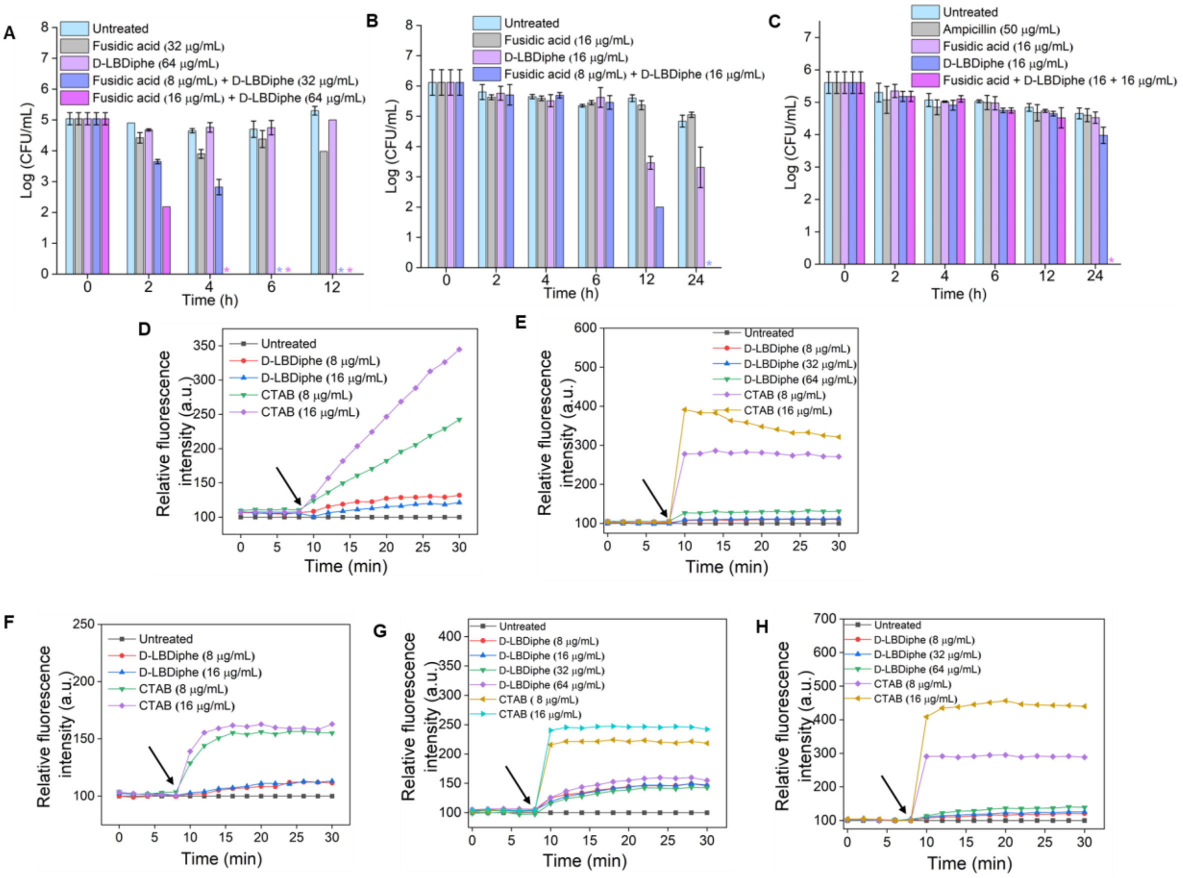

Figure S12. Activity of combination therapy against growth-restricted phases of bacteria and mechanism of action (A) Activity of combination of fusidic acid and D-LBDiphe against stationary phase *P. aeruginosa* R590 (N = 2) (B) Activity of combination of fusidic acid and D-LBDiphe against stationary phase MRSA (N = 2) (C) Activity of combination of fusidic acid and D-LBDiphe against ampicillin-generated *S. aureus* 737 persisters (N = 2). Asterisks indicate < 50 CFU/mL. (D) Membrane permeabilization of D-LBDiphe and CTAB against stationary phase MRSA (E) Membrane depolarization of D-LBDiphe and CTAB against stationary phase MRSA (F) Inner membrane permeabilization of D-LBDiphe and CTAB against stationary phase *P. aeruginosa* R590 (G) Outer membrane permeabilization of D-LBDiphe and CTAB against stationary phase *P. aeruginosa* R590 (H) Membrane depolarization of D-LBDiphe and CTAB against stationary phase *P. aeruginosa* R590. Arrows in (D)-(H) indicate the time of compound addition.

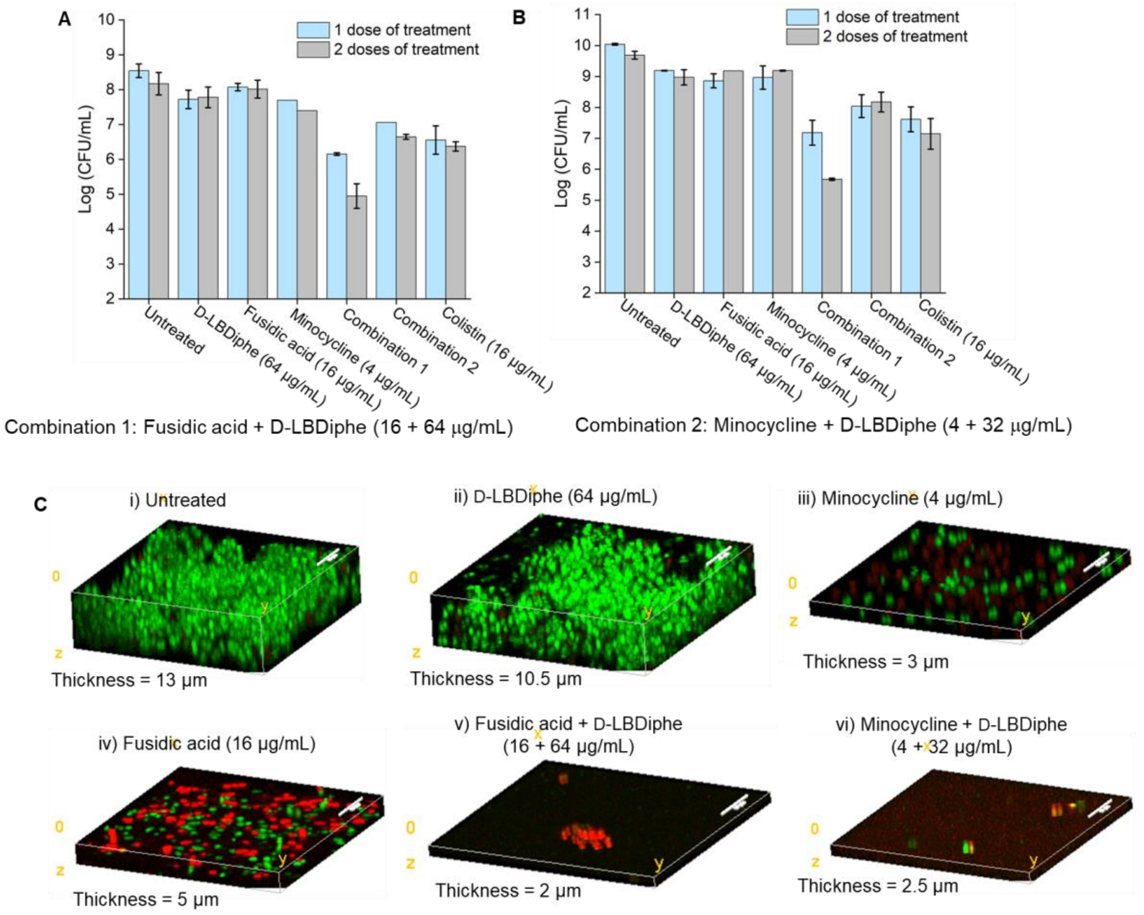

Figure S13. Activity of combination therapy against *A. baumannii* R674 biofilms and dispersed cells (N = 2). (A) Viability of bacteria embedded in biofilm (B) Viability of dispersed phase cells. C) Confocal laser scanning microscopy of biofilms left i) Untreated or when treated with ii) D-LBDiphe (64 µg/mL) iii) Minocycline (4 µg/mL) iv) Fusidic acid (16 µg/mL) v) Fusidic acid + D-LBDiphe (16 + 64 µg/mL) vi) Minocycline + D-LBDiphe (4 + 32 µg/mL). Scale bar in CLSM images is 10 µm.

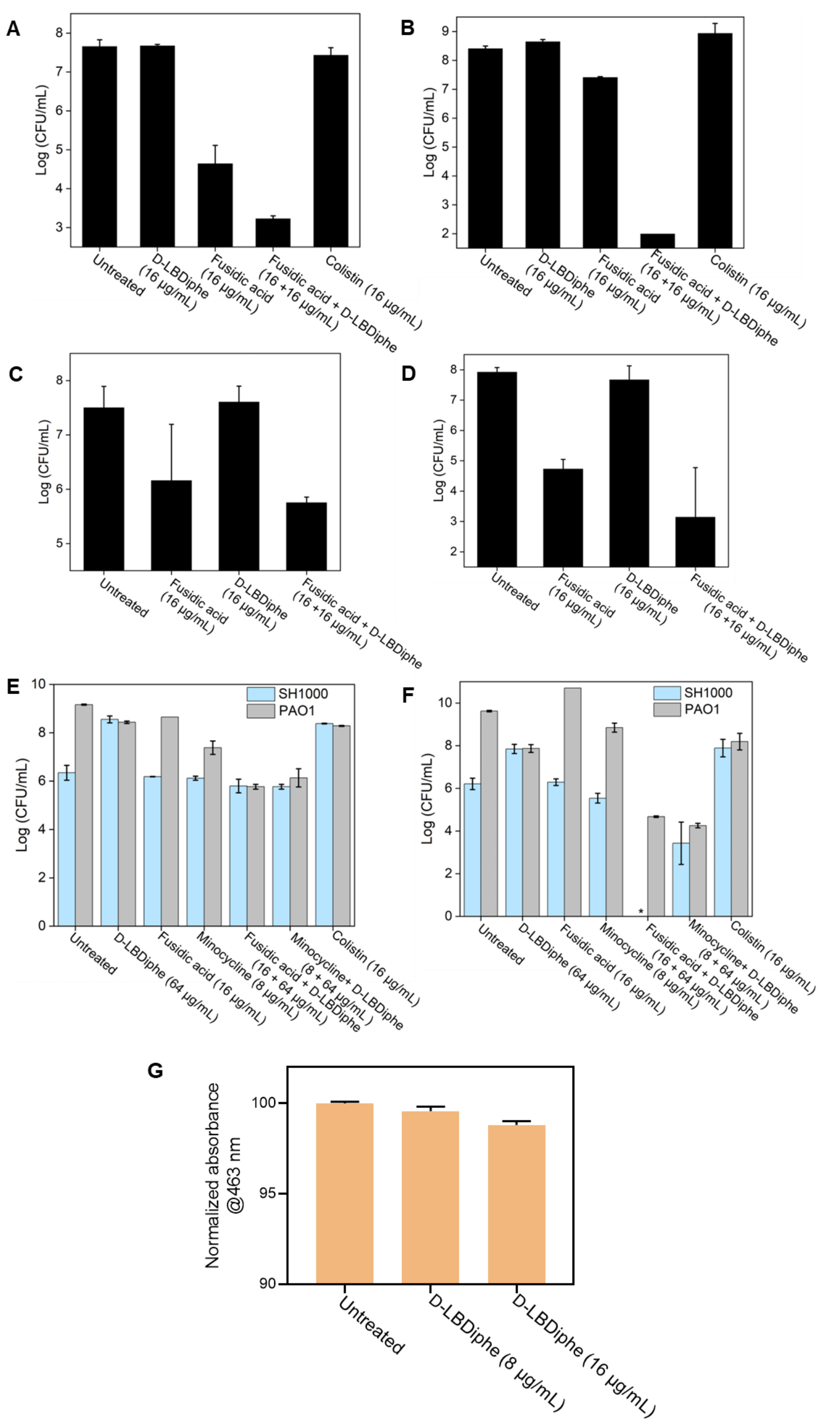

Figure S14. Activity against *P. aeruginosa* R590 and MRSA biofilms and investigation of staphyloxanthin levels in MRSA (A) Viability of bacteria in biofilm of *P. aeruginosa* R590 after two doses of different treatments (N = 2) (B) Viability of dispersed cells of *P. aeruginosa* R590 biofilm after different treatments (N = 2) (C) Viability of bacteria in MRSA biofilm (N = 2) (D) Viability of dispersed cells of MRSA biofilm after different treatments (N = 2) (E) Viability of bacteria in mixed species biofilm of SH1000 and PAO1 (N = 2) (F) Viability of dispersed cells of biofilm of SH1000 and PAO1 (N = 2) (G) Assessment of staphyloxanthin levels in MRSA after 24 h treatment with D-LBDiphe (n = 3) In (G), results between untreated control and D-LBDiphe (8 µg/mL) were ns (One-way ANOVA) and results between untreated control and D-LBDiphe (16 µg/mL) were significant with P = 0.0136 (One-way ANOVA).

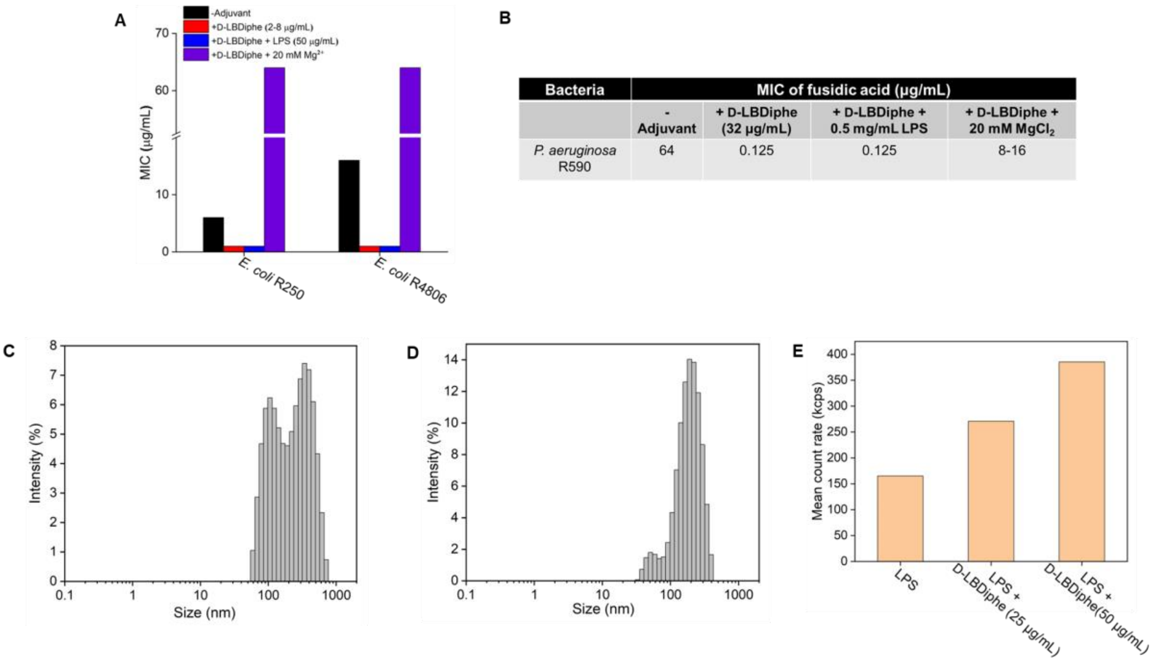

Bacteria	MIC of fusidic acid (μg/mL)			
	- Adjuvant	+ D-LBDiphe (32 μg/mL)	+ D-LBDiphe + 0.5 mg/mL LPS	+ D-LBDiphe + 20 mM $MgCl_2$
P. aeruginosa R590	64	0.125	0.125	8-16

Figure S15. Antagonization of potentiation of D-LBDiphe in presence of exogenous LPS and $MgCl_2$ (A) Effect on MIC of minocycline in combination with D-LBDiphe upon addition of exogenous LPS or $MgCl_2$ against strains of *E. coli* (B) Effect on MIC of fusidic acid in combination with D-LBDiphe upon addition of exogenous LPS or $MgCl_2$ against *P. aeruginosa* R590. Interaction of LPS aggregates with D-LBDiphe through Dynamic light scattering studies. (C) Intensity-size distribution of LPS aggregates at 25 μg/mL (D) Intensity-size distribution of LPS aggregates at 25 μg/mL in presence of 25 μg/mL of D-LBDiphe (E) Comparison of mean count rate (kcps).

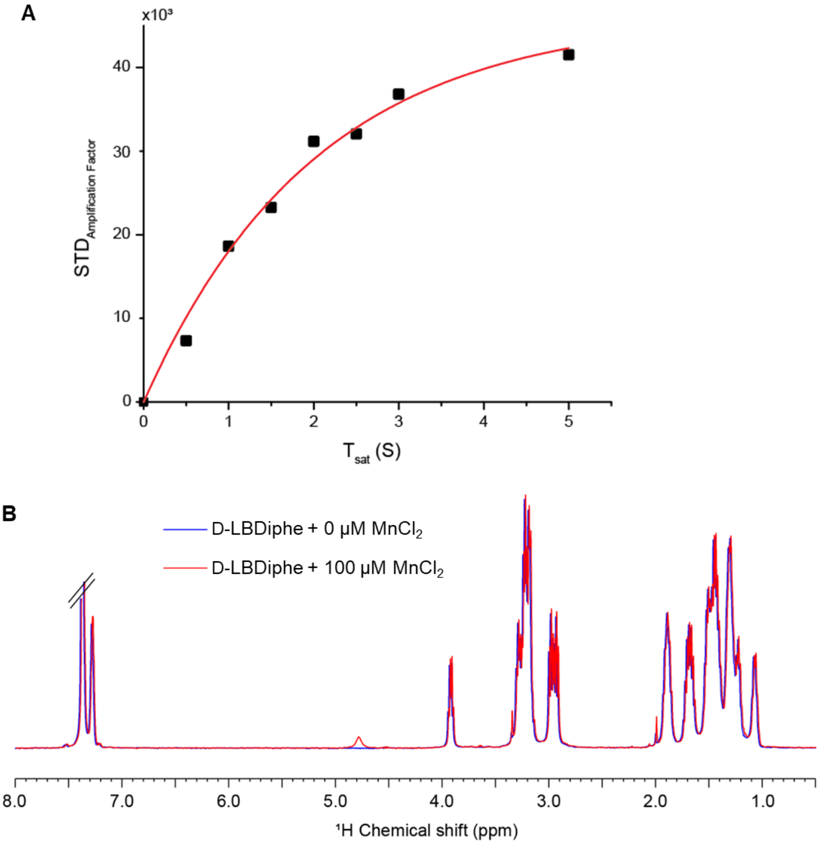

Figure S16. (A) STD build-up curve of the aromatic ring proton of D-LBDiphe in the presence of *E. coli* 0111: B4 LPS micelle at various saturation times. (B) Comparison of the 1D -^{1}H profile of the D-LBDiphe spectrum in the absence and presence of $MnCl_2$ which shows no characteristic changes in the spectrum.

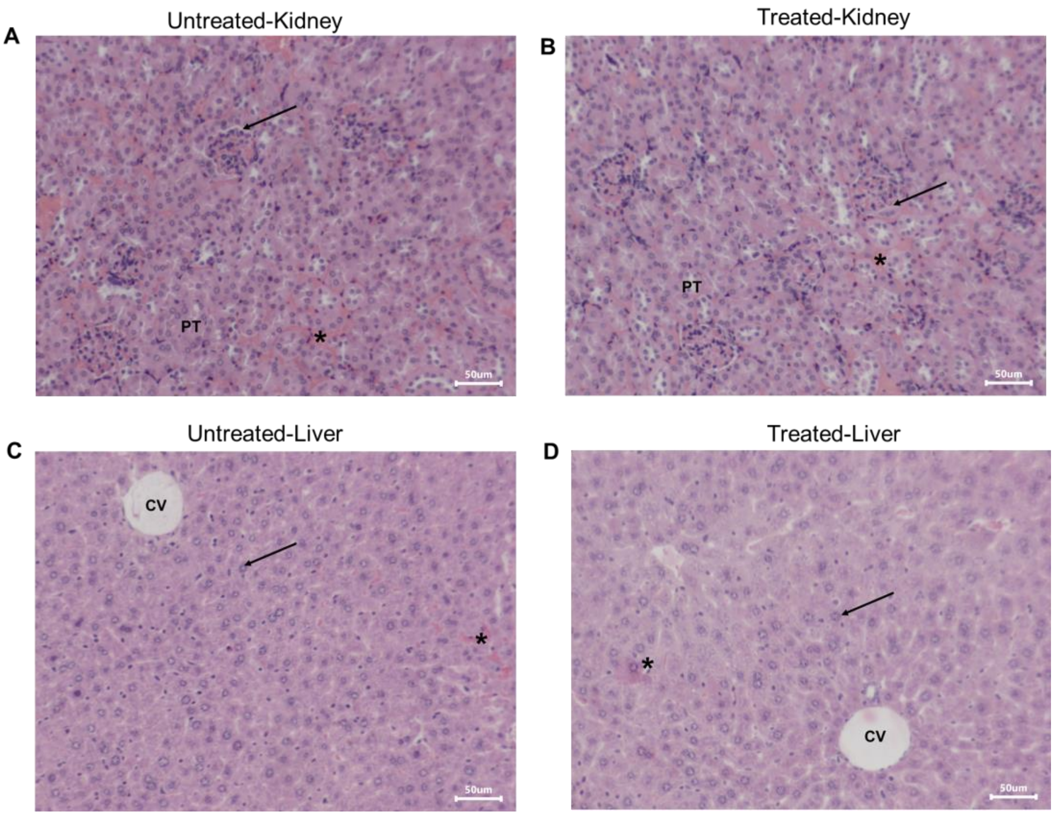

Figure S17. Representative histology (haematoxylin and eosin staining) of kidney sections of BALB/c mice untreated (A) or treated (B) with intraperitoneal injection of minocycline + D-LBDiphe (30 mg/kg + 30 mg/kg). Both the untreated and treated kidney sections looked similar. Kidney showing the presence of glomerulus containing the tuft of capillaries and surrounded by Bowman's capsule (arrow). The proximal convoluted tubules (PT) lined by columnar epithelial cells with basally placed nucleus was observed. The interstitial space contains the infiltration of red blood cells (asterisk). Representative histology (haematoxylin and eosin staining) of liver sections of BALB/c mice untreated (C) or treated (D) with intraperitoneal injection of minocycline + D-LBDiphe (30 mg/kg + 30 mg/kg). Liver showing the hepatocytes mainly containing homogenous cytoplasm staining pink and vesicular nucleus staining blue in colour (arrow). The hepatocytes are arranged in hexagonal fashion surrounding the central vein (CV). The sinusoidal spaces containing Kupffer cells and red blood cells (asterisk) were observed. Haematoxylin and Eosin stain, Scale bar = 50µm. Isolation of kidney and liver was done 2-days post-treatment.

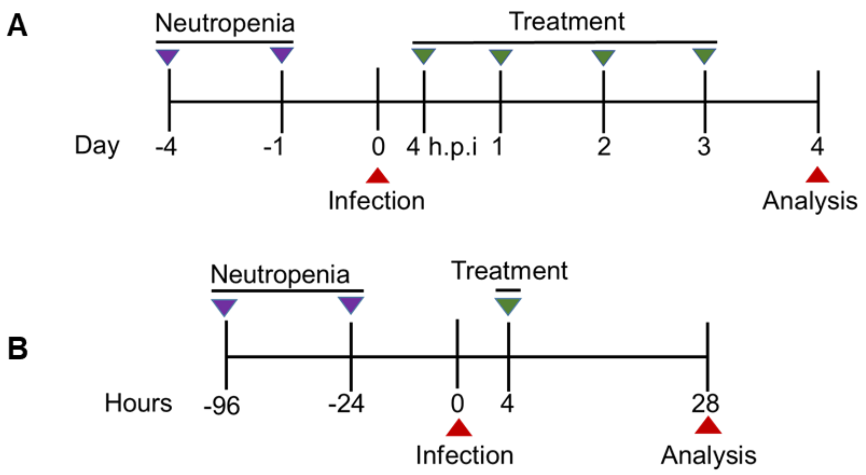

Figure S18. Schematics for *in-vivo* infection studies in mice (A) Schematic for testing *in-vivo* efficacy against acute skin infection of *P. aeruginosa* MTCC 424. 'h.p.i' stands for hours post infection. (B) Schematic for testing *in-vivo* efficacy against pulmonary infection of *P. aeruginosa* PAO1.

Table S1. Calculated logP values of compounds (1-12) using RDKit

**Compound**	**Calculated logP**
NNaph (1)	1.9
NDiphe (2)	2.73
NAda (3)	0.93
LNNaph (4)	1.14
LNDiphe (5)	1.96
LNAda (6)	0.15
BNaph (7)	4.25
BDiphe (8)	5.07
BAda (9)	3.27
LBNaph (10)	3.48
LBDiphe (11)	4.3
LBAda (12)	2.49

Table S2. Potentiation ability of compounds 1-12.

Treatment	MIC (µg/mL)	MIC of antibiotics (µg/mL)			
		Rifampicin	Tetracycline	Rifampicin	Tetracycline
		A. baumannii R674		*P. aeruginosa* R590	
No adjuvant	-	64	128	64-128	128
NNaph (1)	>512	>64	64	>64	>64
NDiphe (2)	>512	32	32	64	>64
NAda (3)	>512	0.5	>64	1	>64
LNNaph (4)	>512	32	64	32	>64
LNDiphe (5)	>512	8	>64	8	64
LNAda (6)	>512	8	16	4	16
BNaph (7)	>512	8	32	16	32
BDiphe (8)	128	0.5	64	1	16
Bada (9)	64	<0.125	>64	0.125	64
LBNaph (10)	512	<0.5	32	<0.5	32
LBDiphe (11)	512	<0.125	16	<0.125	16
LBAda (12)	128	<0.125	4	<0.125	16

For rifampicin, data is presented at adjuvant concentration of 16 µg/mL. For tetracycline, data is presented at adjuvant concentration of 64 µg/mL except for BAda and LBAda whose concentrations are 16 µg/mL.

Table S3. Potentiation activity of antibiotics in presence of LBDiphe against multidrug-resistant Gram-negative isolates.

Antibiotics and bacterial strain	$MIC_{LBDiphe}$ (µg/mL)	$MIC_{antibiotic}$ (µg/mL)	MIC of antibiotic (µg/mL) in presence of LBDiphe (conc. of LBDiphe in µg/mL used in combination)	FICI
**Rifampicin**				
P. aeruginosa R590	512	64-128	0.125 (16)	0.033
P. aeruginosa R596	512	16	0.125 (32)	0.07
P. aeruginosa MTCC 424	64	16	0.06 (16)	0.253
P. aeruginosa R2477	> 512	16	0.06(32)	< 0.066
P. aeruginosa 2536	512	512	0.125 (32)	0.063
K. pneumoniae R3934	> 512	512	4 (4)	< 0.015

K. pneumoniae BAA ATCC 2146	> 512	256	0.06 (2)	< 0.004
K. pneumoniae R3421	> 512	> 512	2 (16)	< 0.035
K. pneumoniae ATCC 700603	> 512	16	0.06 (16)	< 0.035
A. baumannii R674	512	64	0.125 (8)	0.017
A. baumannii R676	256	4	< 0.125 (1)	< 0.035
E. coli R3336	> 512	512	4 (4)	< 0.015
E. coli R250	128	16	0.5 (4)	0.062
E. coli R597	128	8	0.125 (16)	0.14
E. coli R4806	256	8	0.06 (16)	0.07
**Fusidic acid**				
P. aeruginosa R590	512	64	1 (2)	0.019
P. aeruginosa R596	512	> 512	16 (64)	< 0.156
P. aeruginosa MTCC 424	64	256	0.5 (16)	0.25
P. aeruginosa R2477	> 512	> 512	8 (64)	< 0.14
P. aeruginosa 2536	512	> 512	8 (64)	< 0.14
K. pneumoniae R3934	> 512	512	2-4 (64)	< 0.129
K. pneumoniae BAA ATCC 2146	> 512	> 512	8 (8)	< 0.031
K. pneumoniae R3421	> 512	> 512	32 (32)	< 0.125
K. pneumoniae ATCC 700603	> 512	> 512	16 932)	< 0.094
A. baumannii R674	512	64	0.125 (16)	0.033
A. baumannii R676	256	512	0.5 (64)	0.004
E. coli R3336	> 512	> 512	32 (4)	< 0.07
E. coli R250	128	> 512	8 (4)	< 0.047
E. coli R597	128	> 512	8 (8)	< 0.078
E. coli R4806	256	> 512	1 (32)	0.127
**Minocycline**				
P. aeruginosa R590	512	8	1 (2)	0.129
P. aeruginosa R596	512	32	0.25 (32)	0.07
P. aeruginosa MTCC 424	64	4	0.06 (8)	0.14
P. aeruginosa R2477	> 512	32	0.25 (32)	< 0.07

P. aeruginosa 2536	512	32	0.5 (16)	0.047
K. pneumoniae R3934	> 512	64	1 (4)	< 0.023
K. pneumoniae BAA ATCC 2146	> 512	32	0.25 (4)	< 0.015
K. pneumoniae R3421	> 512	64	0.25 (32)	< 0.066
K. pneumoniae ATCC 700603	> 512	16	0.5 (4)	< 0.039
A. baumannii R674	512	8	1 (2)	0.129
A. baumannii R676	256	16	1 (8)	0.094
E. coli R3336	> 512	32	0.5 (2)	< 0.019
E. coli R250	128	4-8	0.25 (2)	0.047
E. coli R597	128	16	2 (4)	0.156
E. coli R4806	256	16	1 (8)	0.093
**Doxycycline**				
P. aeruginosa R590	512	64	8 (2)	0.129
P. aeruginosa R596	512	128	2 (16)	0.047
P. aeruginosa MTCC 424	64	16	0.25 (4)	0.078
P. aeruginosa R2477	> 512	128	2 (8)	< 0.031
P. aeruginosa 2536	512	128	2 (16)	0.047
K. pneumoniae R3934	> 512	128	8 (16)	< 0.094
K. pneumoniae BAA ATCC 2146	> 512	128	0.5 (2)	< 0.008
K. pneumoniae R3421	> 512	64	1 (32)	< 0.08
K. pneumoniae ATCC 700603	> 512	64	1 (32)	< 0.078
A. baumannii R674	512	64	8 (2)	0.129
A. baumannii R676	256	64	8 (64)	0.312
E. coli R3336	> 512	128	1 (2)	< 0.019
E. coli R250	128	8	0.25 (4)	0.062
E. coli R597	128	64	16 (32)	0.5
E. coli R4806	256	64	8 (32)	0.25
**Linezolid**				
P. aeruginosa R590	512	256	32 (16)	0.156
P. aeruginosa R596	512	> 256	> 32 (64)	N/A[b]

P. aeruginosa MTCC 424	64	> 256	8 (16)	< 0.28
P. aeruginosa R2477	> 512	> 256	32 (64)	< 0.25
P. aeruginosa 2536	512	> 256	32 (64)	< 0.25
K. pneumoniae R3934	> 512	> 256	> 32 (64)	N/A
K. pneumoniae BAA ATCC 2146	> 512	> 256	> 32 (64)	N/A
K. pneumoniae R3421	> 512	> 256	> 32 (64)	N/A
K. pneumoniae ATCC 700603	> 512	> 256	> 32 (64)	N/A
A. baumannii R674	512	256	32 (16)	0.156
A. baumannii R676	256	128	16 (64)	0.375
E. coli R3336	> 512	128	> 32 (64)	N/A
E. coli R250	128	> 256	> 32 (64)	N/A
E. coli R597	128	256	16 (64)	0.56
E. coli R4806	256	256	16 (64)	0.312

[b]N/A stands for not applicable

Table S4. Susceptibility of bacterial strains to various antibiotics.

**Bacterial strain**	**MIC of antibiotics (μg/mL)**					
	Doxycycline	Minocycline	Ciprofloxacin	Mem	Gentamicin	Colistin
P. aeruginosa R590	64	8	256	64	>512	1
P. aeruginosa R596	128	32	-	-	-	2
P. aeruginosa MTCC 424	16	4	0.5	0.12	1	1
P. aeruginosa R2477	128	32	32	>64	>512	1
P. aeruginosa 2536	128	32	128	>64	>512	1
K. pneumoniae R3934	128	64	>512	64	>512	2
K. pneumoniae BAA ATCC 2146	128	32	>512	32	>512	1
K. pneumoniae R3421	64	64	512	>64	>512	0.5
K. pneumoniae ATCC 700603	64	16	<0.5	0.06	16	0.5
A. baumannii R674	64	8	128-256	64	>512	0.5-1

A. baumannii R676	64	16	256	-	-	1
E. coli R3336	128	32	256	>64	>512	2
E. coli R250	8	4-8	256	64	>512	1-2
E. coli R597	64	16	256	32	>512	-
E. coli R4806	64	16	128	32	>512	-

Mem stands for meropenem.

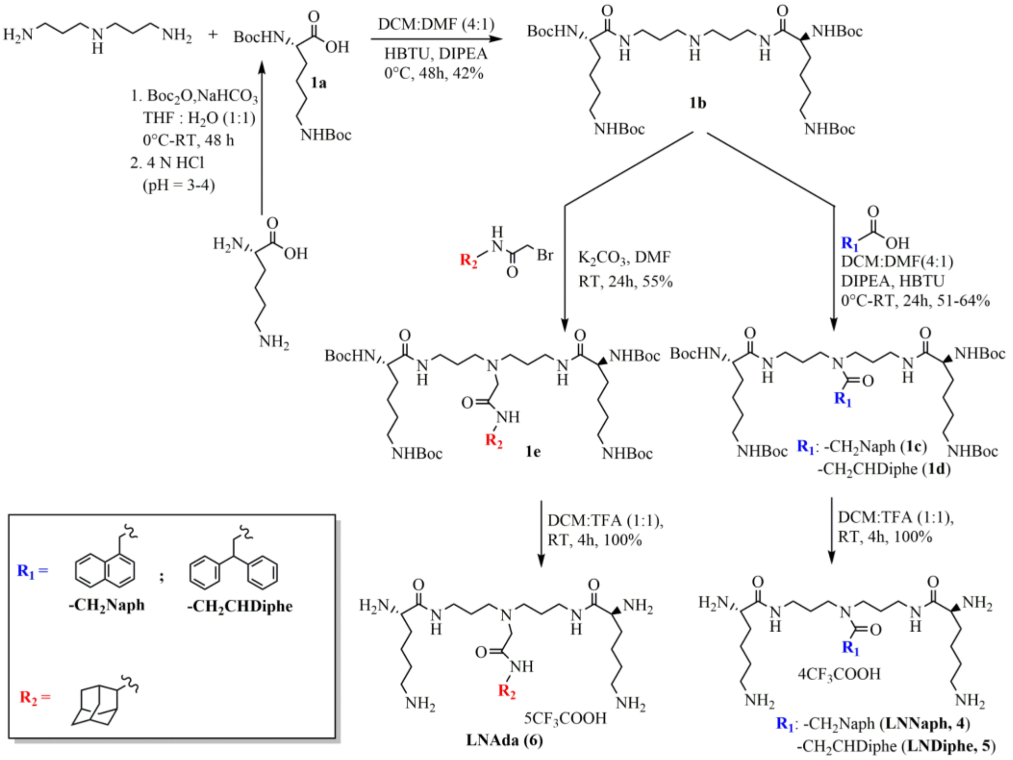

Scheme S1. Synthesis of lysine conjugated norspermidine derivatives (Set 2).

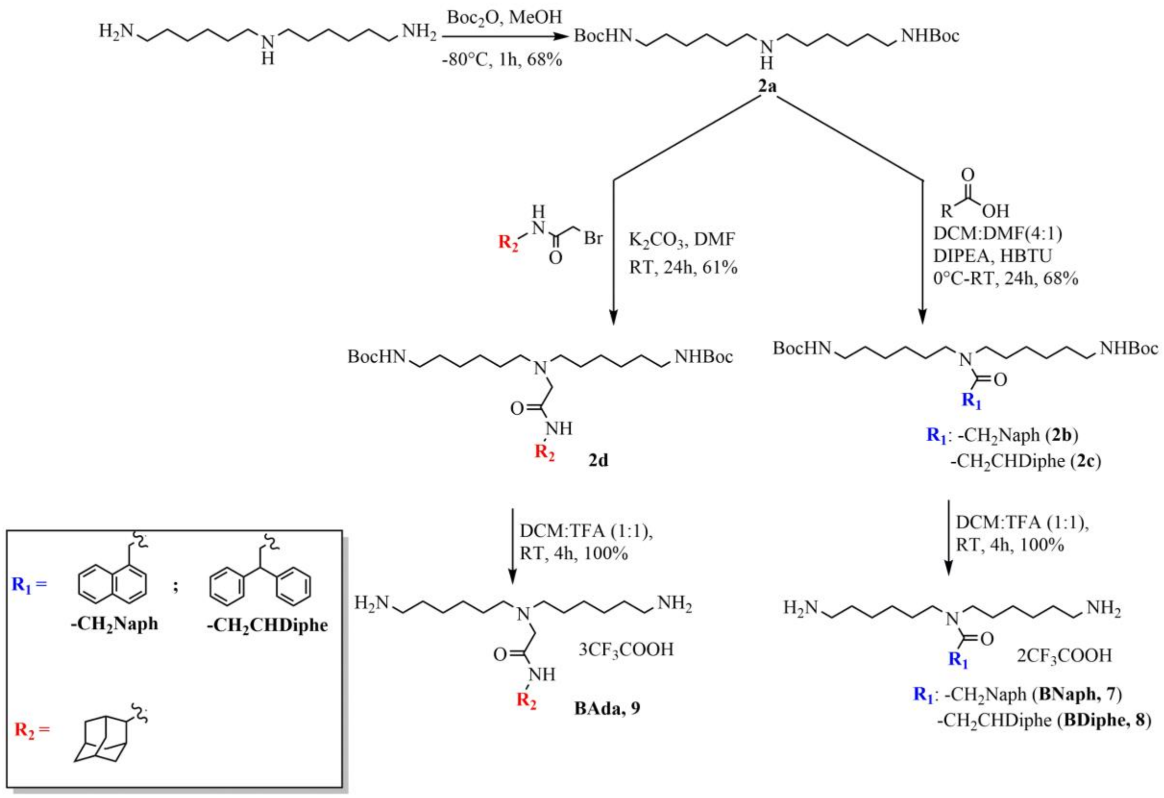

Scheme S2. Synthesis of bis(hexamethylene) triamine derivatives (Set 3).

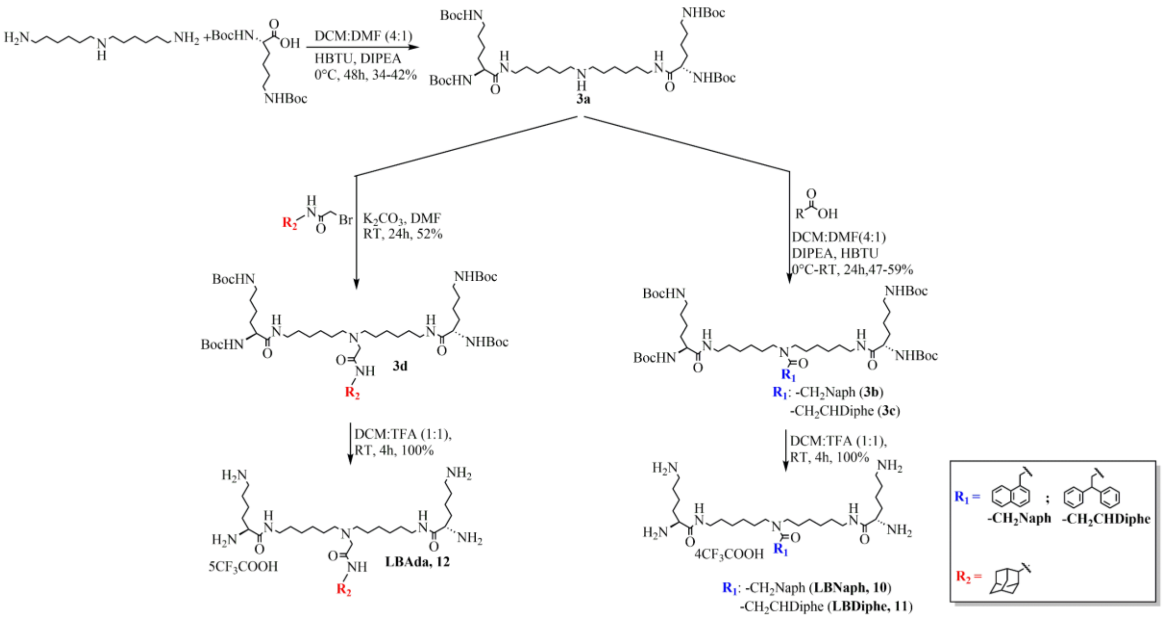

Scheme S3. Synthesis of lysine-conjugated bis(hexamethylene) triamine derivatives (Set 4).

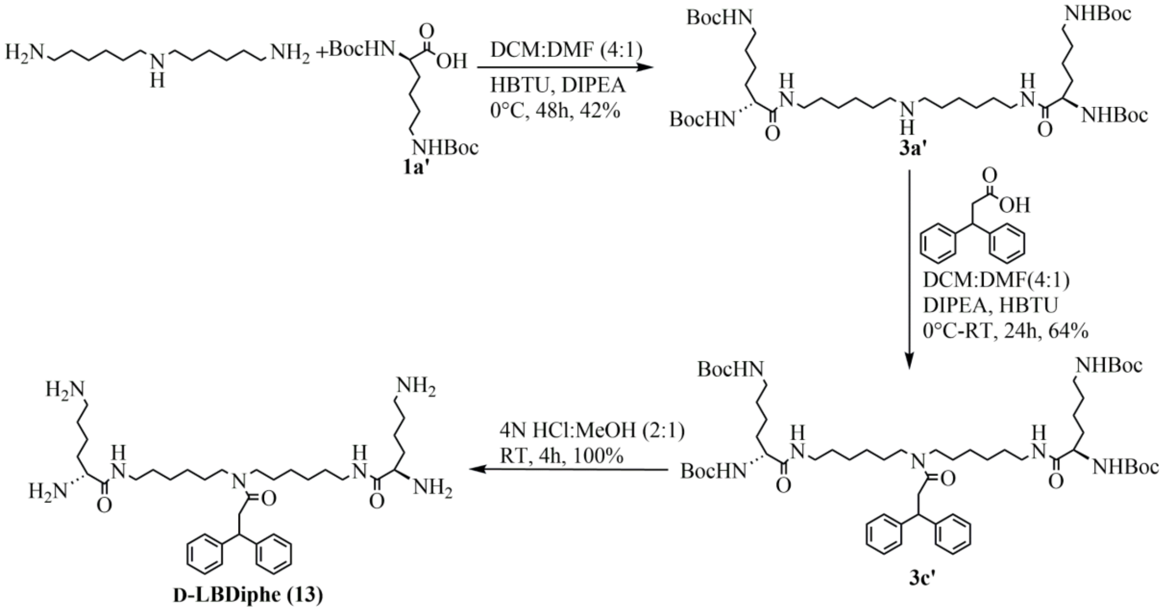

Scheme S4. Synthesis of D-LBDiphe

Experimental section

Materials and instrumentation

All the solvents were of reagent grade, which were distilled and dried before their use. All the reagents were purchased from Sigma-Aldrich, Alfa-Aesar, S.D. Fine, Avra and Spectrochem Pvt. Ltd. They were used without further purification. Analytical thin layer chromatography (TLC) was performed on E. Merck TLC plates pre-coated with silica gel 60 F254 and visualization was carried out using UV light, iodine and ninhydrin solution. Column chromatography was performed on silica gel (60-120 mesh) using different ratios of chloroform and methanol solvent system. Nuclear magnetic resonance spectra were recorded on Bruker AV-400 (400 MHz for ^{1}H and 100 MHz for ^{13}C) spectrometer in deuterated solvents. ^{13}C spectra were also recorded in JEOL-600 MHz (150 MHz for ^{13}C) spectrometer in some cases. The chemical shifts (δ) are reported in parts per million downfield from the peak for the internal standard TMS. High resolution mass spectra (HR-MS) mass spectra were obtained using 6538-UHD Accurate mass Q-TOF LC-MS instrument. Zeiss 510 Meta confocal laser-scanning microscope was used for confocal imaging. All NMR spectra for Saturation transfer difference NMR experiments were recorded on a Bruker Avance III 500 MHz spectrometer, equipped with 5 mm RT probe at 298 K. Data acquisition and processing were performed with Topspin 3.1 software (Bruker). Human RBCs and human PBMCs were isolated from fresh

blood and used for hemolytic assay. HEK 293 cell line was purchased from ATCC. ELISA kits for quantifying cytokine levels were obtained from ABclonal Technologies. Dynamic Light Scattering (DLS) studies were performed using Zetasizer Nano Z (Malvern Instruments) at room temperature in JNCASR. Isothermal calorimetry studies were performed using TA-affinity ITC (TA Instruments, New Castle, USA). Eppendorf 5810R centrifuge was used. TECAN (Infinite series, M200 pro) Plate Reader was used to measure absorbance and fluorescence of 96-well plate. Zeiss 510 Meta confocal laser scanning microscope was used for confocal imaging. Flow cytometry studies were performed in BD Biosciences flow cytometer at JNCASR. Bacterial strains were obtained from American Type Culture Collection (ATCC) or Microbial Type Culture Collection (MTCC). Multidrug-resistant bacterial strains; were obtained from National Institute of Mental Health and NeuroSciences (NIMHANS), Bengaluru, India. GFP-tagged *S. aureus* SH1000 and dsred-tagged *P. aeruginosa* PAO1 were obtained by the courtesy of Prof. Jessica Willis, University of Sheffield, UK. Lipopolysaccharide was obtained from Sigma Aldrich. Eight-ten weeks old pathogen free Balb/c female mice weighing 20 to 24 g were used for *in-vivo* studies. The *in-vivo* animal experiments were done by following appropriate protocols, approved, and permitted by the Institutional Animal Ethics Committee (IAEC) of Jawaharlal Nehru Centre for Advanced Scientific Research (JNCASR) (201/Go/ReBi/S/2000/CPCSEA).

Synthesis and characterization

For synthesis and characterization of Set 1 compounds i.e. NNaph (1), NDiphe (2) and NAda (3), refer to previously published work.[1]

Synthesis of adjuvants in Scheme S1

Synthesis of *N,N′*-Diboc ^{L}Lysine (1a): Briefly, about 10 g (1 equivalent) of ^{L}Lysine hydrochloride was dissolved in 100 mL water, 13.8 g of $NaHCO_3$ (3 equivalents) added and the reaction mixture was kept for stirring at 0 °C. Then, 14.4 g (1.2 equivalents) of $(Boc)_2O$ dissolved in 50-75 mL of THF was added slowly using a dropper funnel. The reaction mixture was allowed to stir for some time at 0 □ (till the addition of Boc_2O was complete) and then kept at RT. After 16 hours, 14.4 g (1.2 equivalents) of Boc_2O dissolved in 50 mL THF was added using a dropper funnel. The reaction was carried on for 32 more hours. THF was evaporated so that the product dissolves in water. The reaction mixture was acidified with 4N HCl till turbidity appeared. The product was extracted using $CHCl_3$. The pure product (acid form) is obtained after evaporating the solvent. ^{1}H-NMR (400 MHz, $CDCl_3$) δ/ppm: 5.225 (br, N*H*Boc, 1H), 4.661-4.133 (br, COOH-CH(NHBoc)-$(CH_2)_3$-CH_2-NHBoc, 2H), 3.768-3.736 (t,

COOH-C*H*(NHBoc), 1H), 3.125-3.115 (br, COOH-CH(NHBoc)-C*H₂*-(CH$_2$)$_3$-NHBoc, 2H), 1.871-1.445 (br, COOH-CH(NH-COO-C(C*H*$_3$)$_3$)-CH$_2$-(C*H*$_2$)$_2$-CH$_2$-NH-COO-C(C*H*$_3$)$_3$, 22H). HRMS (m/z): 369.1930 [(M+Na)$^+$] (Observed), 369.2002 [(M+Na)$^+$] (Calculated).

For *N,N′*-Diboc ^{D}Lysine (1a′), ^{D}Lysine was used and the same protocol was followed. ^{1}H-NMR (400 MHz, CDCl$_3$) δ/ppm: 9.499 (br, N*H*Boc, 1H), 6.181 (br, N*H*Boc, 1H), 5.337-5.317 (m, COOH-C*H*(NHBoc), 1H), 4.458-4.073 (m, COOH-CH(NHBoc)-(CH$_2$)$_3$-C*H*$_2$-NHBoc, 2H), 3.079 (bs, COOH-CH(NHBoc)-C*H*$_2$-(CH$_2$)$_3$-NHBoc, 2H), 1.837-1.821 (m, COOH-CH(NH-COO-C(CH$_3$)$_3$)-CH$_2$-(C*H*$_2$)$_2$-CH$_2$-NH-COO-C(CH$_3$)$_3$, 2H), 1.703-1.415 (br, COOH-CH(NH-COO-C(C*H*$_3$)$_3$)-CH$_2$-(C*H*$_2$)$_2$-CH$_2$-NH-COO-C(C*H*$_3$)$_3$, 20H).

Synthesis of *N*^{1}-(Boc-^{L}Lys)-*N*^{3}-[{3-(Boc-^{L}Lys) amido} propyl] propane-1,3-diamine (1b): About 3.17 g (2.4 equivalents, 9.14 mmol) of *N, N′*-DiBoc-^{L}Lysine was dissolved in about 25 mL of dry DCM at 0 °C. In the reaction mixture about 3.32 mL (5 equivalents, 19.05 mmol) of DIPEA was added followed by about 3.47 g (2.4 equivalents, 9.14 mmol) of HBTU. Now about 8 mL of DMF was added to the reaction mixture. After 10 minutes, about 500 mg (1 equivalent, 3.81 mmol) of norspermidine, dissolved in 10 mL of DCM was added drop wise to the reaction mixture. The reaction mixture was allowed to stir for 48 h at 0 °C. Then reaction solvent was evaporated, and residue was diluted in ethyl acetate. Thereafter work-up was done with 1N HCl (50 mL, 3 times) followed by saturated Na_2CO_3 solution (50 mL, 3 times). The crude product was collected in ethyl acetate layer. Finally, column was done using different ratios of chloroform and methanol solution, to isolate the product with 42% yield. ^{1}H-NMR (400 MHz, CDCl$_3$) δ/ppm: 7.337 (br, NH(-(CH$_2$)$_3$-N*H*-CO-CH(NHBoc)-(CH$_2$)$_4$-NHBoc)$_2$, 2H), 5.566 (br, NH(-(CH$_2$)$_3$-NH-CO-CH(N*H*Boc)-(CH$_2$)$_4$-NHBoc)$_2$, 2H), 4.808 (br, NH(-(CH$_2$)$_3$-NH-CO-CH(NHBoc)-(CH$_2$)$_4$-N*H*Boc)$_2$, 2H), 4.053 (br, NH(-(CH$_2$)$_3$-NH-CO-C*H*(NHBoc)-(CH$_2$)$_4$-NHBoc)$_2$, 2H), 3.475-3.323 and 3.102-3.088 (br, NH(-(CH$_2$)$_2$-C*H*$_2$-NH-CO-CH(NHBoc)-(CH$_2$)$_3$-C*H*$_2$-NHBoc)$_2$, 8H), 2.745 (br, NH(-C*H*$_2$-(CH$_2$)$_2$-NH-CO-CH(NHBoc)-(CH$_2$)$_4$-NHBoc)$_2$, 4H), 2.349 and 1.802-1.788 (br, NH(-CH$_2$-C*H*$_2$-CH$_2$-NH-CO-CH(NHBoc)-C*H*$_2$-(CH$_2$)$_3$-NHBoc)$_2$, 8H), 1.666-1.580 (m, NH(-CH$_2$-CH$_2$-CH$_2$-NH-CO-CH(NHBoc)-(CH$_2$)$_2$-C*H*$_2$-CH$_2$-NHBoc)$_2$, 4H), 1.422 (br, NH(-(CH$_2$)$_3$-NH-CO-CH(NH-COO-C(C*H*$_3$)$_3$)-CH$_2$-C*H*$_2$-CH$_2$-CH$_2$-NH-COO-C(C*H*$_3$)$_3$)$_2$, 40H). HRMS (m/z): 788.5462 [(M+H)$^+$] (Observed), 788.5497 [(M+H)$^+$] (Calculated).

Synthesis of *N,N*-bis-[{3-(Boc-^{L}Lys)amido} propyl] naphthyl ethanamide (1c): 113.4 mg (1.2 equivalents, 0.609 mmol) of Naphthyl acetic acid was dissolved in dry DCM (4 mL) at 0

°C. In the reaction mixture, 265 μL (3 equivalents, 1.52 mmol) of *N,N*-diisopropylethylamine (DIPEA) was added followed by 231 mg (1.2 equivalents, 0.609 mmol) of *N,N,N',N'*-tetramethyl-O-(1H-benzotriazol-1-yl)uronium hexafluorophosphate (HBTU). Then DMF (2 mL) was added to the reaction mixture to dissolve the HBTU. After 15 min, 400 mg (1 equivalent, 0.51 mmol) of N^1-(Boc-^{L}Lys)-N^3-[{3-(Boc-^{L}Lys) amido} propyl] propane-1,3-diamine, **1b** dissolved in dry DCM (4 mL) was added dropwise to the reaction mixture. The reaction mixture was brought to room temperature and allowed to stir for 24 h. Solvent was evaporated, and residue was diluted in ethyl acetate (50 mL). Then workup was carried out first with 1 N HCl (50 mL, 3 times) followed by saturated Na_2CO_3 solution (50 mL, 3 times). The crude product was extracted in ethyl acetate layer. Finally, purification was accomplished through column chromatography on silica gel (60−120 mesh) using di□erent ratios of methanol and chloroform as eluent to a□ord compound **1c** with 51% yield. ^{1}H-NMR (400 MHz, $CDCl_3$) δ/ppm: 7.930-7.909 (m, Ar$\underline{H}$, 1H), 7.866-7.847 (m, Ar$\underline{H}$, 1H), 7.844-7.775 (m, Ar$\underline{H}$, 1H), 7.536-7.482 (m, Ar$\underline{H}$, 2H), 7.465-7.407 (m, Ar$\underline{H}$, 1H), 7.387-7.321 (m, Ar$\underline{H}$, 1H), 7.172 (br, R-CO-N(-(CH$_2$)$_3$-N$\underline{H}$-CO-CH(NHBoc)-(CH$_2$)$_4$-NHBoc)$_2$, 2H), 5.352 (br, R-CO-N(-(CH$_2$)$_3$-NH-CO-CH(N$\underline{H}$Boc)-(CH$_2$)$_4$-NHBoc)$_2$, 2H), 5.257-5.240 (br, R-CO-N(-(CH$_2$)$_3$-NH-CO-CH(NHBoc)-(CH$_2$)$_4$-N$\underline{H}$Boc)$_2$, 2H), 4.667 (br, R-CO-N((-(CH$_2$)$_3$-NH-CO-C$\underline{H}$(NHBoc)-(CH$_2$)$_4$-NHBoc)$_2$, 2H), 4.120 (s, ArCH$_2$-CO-N(-(CH$_2$)$_3$-NH-CO-CH(NHBoc)-(CH$_2$)$_3$-C$\underline{H_2}$-NHBoc)$_2$, 4H), 3.473-3.362 (br, R-CO-N(-(CH$_2$)$_2$-C$\underline{H_2}$-NH-CO-CH(NHBoc)-(CH$_2$)$_4$-NHBoc)$_2$, 4H), 3.243 (br, ArC$\underline{H_2}$-CO-N(-(CH$_2$)$_3$-NH-CO-CH(NHBoc)-(CH$_2$)$_3$-CH$_2$-NHBoc)$_2$, 2H), 3.063 (t, J = 6.4 Hz, R-CO-N(-C$\underline{H_2}$-(CH$_2$)$_2$-NH-CO-CH(NHBoc)-(CH$_2$)$_3$-CH$_2$-NHBoc)$_2$, 4H), 1.772 (br, R-CO-N(-CH$_2$-C$\underline{H_2}$-CH$_2$-NH-CO-CH(NHBoc)-(CH$_2$)$_3$-CH$_2$-NHBoc)$_2$, 4H), 1.689 (br, R-CO-N(-(CH$_2$)$_3$-NH-CO-CH(NHBoc)-C$\underline{H_2}$-(CH$_2$)$_3$-NHBoc)$_2$, 4H), 1.595 (br, R-CO-N(-(CH$_2$)$_3$-NH-CO-CH(NHBoc)-(CH$_2$)$_2$-C$\underline{H_2}$-CH$_2$-NHBoc)$_2$, 4H), 1.430-1.396 (bs, R-CO-N(-(CH$_2$)$_3$-NH-CO-CH(NH-COO-C(C$\underline{H_3}$)$_3$)-(CH$_2$)$_4$-NH-COO-C(C$\underline{H_3}$)$_3$)$_2$, 36H), 1.254 (br, R-CO-N(-(CH$_2$)$_3$-NH-CO-CH(NHBoc)-CH$_2$-CH$_2$-C$\underline{H_2}$-CH$_2$-CH$_2$-NHBoc)$_2$, 4H). HRMS (m/z): 956.5919 [(M+H)$^+$] (Observed), 956.6072 [(M+H)$^+$] (Calculated).

Synthesis of *N,N*-bis-[{3-(Boc-^{L}Lys)amido} propyl] 3,3-diphenyl propanamide (1d): 275.6 mg (1.2 equivalents, 1.22 mmol) of 3,3-diphenyl propionic acid was dissolved in dry DCM (4 mL) at 0 °C. In the reaction mixture, 530.5 μL (3 equivalents, 3.04 mmol) of *N,N*-diisopropylethylamine (DIPEA) was added followed by 462 mg (1.2 equivalents, 1.22 mmol) of *N,N,N',N'*-tetramethyl-O-(1H-benzotriazol-1-yl)uronium hexafluorophosphate (HBTU). Then DMF (2 mL) was added to the reaction mixture to dissolve the HBTU. After 15 min, 800

mg (1 equivalent, 1.01 mmol) of N^1-(Boc-^{L}Lys)-N^3-[{3-(Boc-^{L}Lys) amido} propyl] propane-1,3-diamine, **1b** dissolved in dry DCM (4 mL) was added dropwise to the reaction mixture. The reaction mixture was brought to room temperature and allowed to stir for 24 h. Solvent was evaporated, and residue was diluted in ethyl acetate (50 mL). Then workup was carried out first with 1 N HCl (50 mL, 3 times) followed by saturated Na_2CO_3 solution (50 mL, 3 times). The crude product was extracted in ethyl acetate layer. Finally, purification was accomplished through column chromatography on silica gel (60−120 mesh) using di□erent ratios of methanol and chloroform as eluent to a□ord compound **1d** with 64% yield. ^{1}H-NMR (400 MHz, $CDCl_3$) δ/ppm: 7.241-7.147 (m, Ar*H*, 10H), 5.345-5.235 (br, -N*H*$_{amide}$, 3H), 4.719-4.682 (m, ArC*H*CH_2CO-N(-$(CH_2)_3$-NH-CO-C*H*(NHBoc)-$(CH_2)_3$-CH_2-NHBoc)$_2$, 3H), 4.146-4.092 (m, -N*H*$_{amide}$, 3H), 3.388-2.788 (m, ArCHC*H*$_2$CO-N(-C*H*$_2$-C*H*$_2$-C*H*$_2$-NH-CO-CH(NHBoc)-$(CH_2)_3$-C*H*$_2$-NHBoc)$_2$, 18H), 1.691-1.590 (br, R-CO-N(-$(CH_2)_3$-NH-CO-CH(NHBoc)-C*H*$_2$-CH_2-C*H*$_2$-CH_2-NHBoc)$_2$, 8H), 1.432-1.422 (bs, R-CO-N(-$(CH_2)_3$-NH-CO-CH(NH-COO-C(C*H*$_3$)$_3$-CH_2-CH_2-CH_2-CH_2-NH-COO-C(C*H*$_3$)$_3$)$_2$, 36H), 1.419-1.338 (br, R-CO-N(-$(CH_2)_3$-NH-CO-CH(NHBoc)-CH_2-C*H*$_2$-CH_2-CH_2-NHBoc)$_2$, 4H). HRMS (m/z): 996.6348 [$(M+H)^+$] (Observed), 996.6385 [$(M+H)^+$] (Calculated).

Synthesis of *N*-(Adamantan-2-yl)-2-bromoethanamide: About 500 mg of 2-Adamantyl amine hydrochloride (1 equivalent, 2.66 mmol) was dissolved in 12 mL DCM and 552 mg of K_2CO_3 (1.5 equivalents, 3.99 mmol) dissolved in 10 mL of water was added to it at 5□. 806.5 mg of Bromoacetyl bromide (1.5 equivalents, 3.99 mmol) was dissolved in 8 mL DCM and slowly added to the reaction mixture using a dropper funnel. The reaction was kept at 5□ for 30 minutes, till the addition of Bromoacetyl bromide and then at room temperature for 12h. After 12h, the reaction was stopped, DCM layer collected, worked up with water 3 times and finally evaporated to get the desired pure product with 85% yield. ^{1}H-NMR (400 MHz, $CDCl_3$) δ/ppm: 6.914 (br, Ad-N*H*-CO-CH_2Br, 1H), 4.046-4.025 (m, Ad*H*, 1H), 3.912 (s, Ad-NH-CO-C*H*$_2$Br, 2H), 1.944-1.612 (m, Ad*H*, 14H).

Synthesis of *N*-(adamantan-2-yl)-2-[*N′*,*N′*-{bis-(3-(Boc-^{L}Lys)amido)propyl} amino] ethanamide (1e): 390 mg of N^1-(Boc-^{L}Lys)-N^3-[{3-(boc-^{L}Lys)amido}propyl] propane-1,6-diamine, **1b** (1 equivalent, 0.77 mmol) in DMF. 154.6 mg of K_2CO_3 (1.5 equivalents, 1.12 mmol) was added to the reaction mixture. Then, 203 mg of *N*-(adamantan-2-yl)-2-bromoethanamide (1 equivalent, 0.77 mmol), dissolved in DMF was added to the reaction mixture. The reaction was stopped after 24 hours. The reaction mixture was then dissolved in ethyl acetate and work-up was performed with ice-cold water (3 times) to remove the DMF.

The ethyl acetate layer was collected, and solvent evaporated to get the pure product with 40% yield. ^{1}H-NMR (400 MHz, $CDCl_3$) δ/ppm: 6.902 (br, AdN*H*-CO-CH_2-N(-CH_2-CH_2-CH_2-NH-CO-CH(NHBoc)-CH_2-CH_2-CH_2-CH_2-NHBoc$)_2$, 1H), 5.590 (br, AdNH-CO-CH_2-N(-CH_2-CH_2-CH_2-N*H*-CO-CH(NHBoc)-CH_2-CH_2-CH_2-CH_2-NHBoc$)_2$, 2H), 4.786 (br, AdNH-CO-CH_2-N(-CH_2-CH_2-CH_2-NH-CO-CH(N*H*Boc)-CH_2-CH_2-CH_2-CH_2-NHBoc$)_2$, 2H), 4.123-4.027 (m, Ad*H* and AdNH-CO-CH_2-N(-CH_2-CH_2-CH_2-NH-CO-C*H*(NHBoc)-CH_2-CH_2-CH_2-CH_2-NHBoc$)_2$, 3H), 3.461 (br, AdNH-CO-CH_2-N(-CH_2-CH_2-CH_2-NH-CO-CH(NHBoc)-CH_2-CH_2-CH_2-CH_2-N*H*Boc$)_2$, 2H), 3.208-2.519 (br, AdNH-CO-C*H_2*-N(-CH_2-CH_2-C*H_2*-NH-CO-CH(NHBoc)-CH_2-CH_2-CH_2-C*H_2*-NHBoc$)_2$, 10H), 2.039-1.479 (br, Ad*H* and AdNH-CO-CH_2-N(-C*H_2*-C*H_2*-CH_2-NH-CO-CH(NHBoc)-C*H_2*-C*H_2*-C*H_2*-CH_2-NHBoc$)_2$, 34H), 1.429-1.422 (bs, AdNH-CO-CH_2-N(-CH_2-CH_2-CH_2-NH-CO-CH(NH-COO-C(C*H_3)$_3$*)-CH_2-CH_2-CH_2-CH_2-NH-COO-C(C*H_3)$_3$*$)_2$, 36H). HRMS (m/z): 979.6745 [$(M+H)^+$] (Observed), 979.6807 [$(M+H)^+$] (Calculated).

General procedure for synthesizing final compounds (LNNaph, LNDiphe and LNAda): At first 1 equivalent of **1c**, **1d** and **1f** was dissolved in dry DCM. To the intensely stirred solution 4 equivalents (excess amount) of trifluoroacetic acid (TFA) was added and allowed to stir at RT for 4h. After that reaction, solvent and unused TFA was removed to get the pure product with 100% yield with CF_3COO^- counterions.

***N,N*-bis-[(6-(^{L}Lys)amido) propyl] naphthyl ethanamide tetrakis(trifluoroacetate) (LNNaph, 4):** ^{1}H-NMR (400 MHz, DMSO-d_6) δ/ppm: 8.581-8.426 (m, Ar*H*, 2H), 8.164-8.130 (m, Ar*H*, -NH_{amide} and -N*H_3*$^+$, 6H), 7.947-7.753 (m, Ar*H*, -N*H*$_{amide}$ and -N*H_3*$^+$, 9H), 7.531-7.316 (m, Ar*H* and -N*H_3*$^+$, 4H), 4.128 (s, ArC*H_2*CO-N(-$(CH_2)_3$-NH-CO- CH(NH_3^+)-$(CH_2)_3$-CH_2-$NH_3^+$$)_2$, 2H), 3.699-3.686 (br, R-CO-N(-$(CH_2)_3$-NH-CO-C*H*(NH_3^+)-$(CH_2)_3$-CH_2-$NH_3^+$$)_2$, 2H), 3.461-3.309 (br, R-CO-N(-$(CH_2)_3$-NH-CO-CH(NH_3^+)-$(CH_2)_3$-C*H_2*-$NH_3^+$$)_2$, 4H), 3.203-3.109 and 2.744-2.694 (br, R-CO-N(-C*H_2*-CH_2-C*H_2*-NH-CO-CH(NH_3^+)-$(CH_2)_3$-CH_2-$NH_3^+$$)_2$, 8H), 1.821-1.656 (m, R-CO-N(-CH_2-CH_2-CH_2-NH-CO-CH(NH_3^+)-C*H_2*-CH_2-C*H_2*-CH_2-$NH_3^+$$)_2$, 8H), 1.533-1.457 (m, R-CO-N(-CH_2-C*H_2*-CH_2-NH-CO-CH(NH_3^+)-CH_2-CH_2-CH_2-CH_2-$NH_3^+$$)_2$, 8H), 1.332-1.235 (m, R-CO-N(-CH_2-CH_2-CH_2-NH-CO-CH(NH_3^+)-CH_2-C*H_2*-CH_2-CH_2-$NH_3^+$$)_2$, 4H). ^{13}C-NMR (150 MHz, DMSO-d_6) δ/ppm: 170.1, 168.5, 168.3, 158.8, 158.6, 158.4,158.2, 133.3,132.8, 132.1, 128.4, 127.2, 127.1, 126.0, 125.6, 125.5, 124.1, 120.2, 118.2, 116.2, 114.2, 52.1, 45.5, 43.2, 39.5, 37.1, 36.7, 36.5, 30.5, 28.4, 27.4, 26.5, 21.2 HRMS (m/z): 556.3870 [$(M+H)^+$] (Observed), 556.3975 [$(M+H)^+$] (Calculated).

***N,N*-bis-[(6-(^{L}Lys)amido) propyl] 3,3-diphenyl propanamide tetrakis(trifluoroacetate) (LNDiphe, 5):** ^{1}H-NMR (400 MHz, DMSO-d_6) δ/ppm: 8.558 (t, $-N\underline{H}_{amide}$, 1H), 8.318 (t, $-N\underline{H}_{amide}$, 1H), 8.172-7.801 (br, $R-CO-N(-CH_2-CH_2-CH_2-NH-CO-CH(N\underline{H_3}^+)-CH_2-CH_2-CH_2-CH_2-N\underline{H_3}^+)_2$, 12H), 7.308-7.130 (m, Ar$\underline{H}$, 10H), 4.539-4.502 (t, *J* = 7.6 Hz, $ArC\underline{H}CH_2-CO-N(-CH_2-CH_2-CH_2-NH-CO-CH(NH_3^+)-CH_2-CH_2-CH_2-CH_2-NH_3^+)_2$, 1H), 3.683-3.670 (br, $ArCHC\underline{H_2}-CO-N(-CH_2-CH_2-CH_2-NH-CO-C\underline{H}(NH_3^+)-CH_2-CH_2-CH_2-CH_2-NH_3^+)_2$, 3H), 3.330-2.730 (m, $R-CO-N(-C\underline{H_2}-CH_2-C\underline{H_2}-NH-CO-CH(NH_3^+)-C\underline{H_2}-CH_2-CH_2-C\underline{H_2}-NH_3^+)_2$, 12H), 1.700-1.235 (m, $R-CO-N(-CH_2-C\underline{H_2}-CH_2-NH-CO-CH(NH_3^+)-CH_2-C\underline{H_2}-C\underline{H_2}-CH_2-NH_3^+)_2$, 16H). ^{13}C-NMR (100 MHz, DMSO-d6) δ/ppm: 170.0, 168.4, 168.1, 158.9, 158.6, 158.3, 158.0, 144.6, 128.4, 128.3, 127.7, 127.5, 126.0, 118.5, 115.5, 52.1, 47.0, 44.9, 43.0, 39.5, 38.4, 37.6, 36.4, 30.5, 28.5, 27.2, 26.6, 26.5, 21.3, 21.2. HRMS (m/z): 596.4248 [$(M+H)^+$] (Observed), 596.4288 [$(M+H)^+$] (Calculated).

***N*-(adamantan-2-yl)-2-[*N´,N´*-{bis-(3-^{L}Lys) amido)propyl} amino] ethanamide pentakis(trifluoroacetate) (LNAda, 7):** ^{1}H-NMR (400 MHz, DMSO-d_6) δ/ppm: 9.688 (br, $AdNH-CO-CH_2-N\underline{H}^+(-CH_2-CH_2-CH_2-NH-CO-CH(NH_3^+)-CH_2-CH_2-CH_2-CH_2-NH_3^+)_2$, 1H), 8.673 (br, $AdNH-CO-CH_2-NH^+(-CH_2-CH_2-CH_2-N\underline{H}-CO-CH(NH_3^+)-CH_2-CH_2-CH_2-CH_2-NH_3^+)_2$, 2H), 8.482 (br, $AdN\underline{H}-CO-CH_2-NH^+(-CH_2-CH_2-CH_2-NH-CO-CH(NH_3^+)-CH_2-CH_2-CH_2-CH_2-NH_3^+)_2$, 1H), 8.207 (bs, $AdNH-CO-CH_2-NH^+(-CH_2-CH_2-CH_2-NH-CO-CH(N\underline{H_3}^+)-CH_2-CH_2-CH_2-CH_2-N\underline{H_3}^+)_2$, 6H), 7.836 (bs, $AdNH-CO-CH_2-NH^+(-CH_2-CH_2-CH_2-NH-CO-CH(N\underline{H_3}^+)-CH_2-CH_2-CH_2-CH_2-N\underline{H_3}^+)_2$, 6H), 3.915-3.864 (m, Ad*H* and $AdNH-CO-CH_2-NH^+(-CH_2-CH_2-CH_2-NH-CO-C\underline{H}(NH_3^+)-CH_2-CH_2-CH_2-CH_2-NH_3^+)_2$, 3H), 3.820 (s, $AdNH-CO-C\underline{H_2}-NH^+(-CH_2-CH_2-CH_2-NH-CO-CH(NH_3^+)-CH_2-CH_2-CH_2-CH_2-NH_3^+)_2$, 2H), 3.167-3.142 (br, $AdNH-CO-CH_2-NH^+(-C\underline{H_2}-CH_2-CH_2-NH-CO-CH(NH_3^+)-CH_2-CH_2-CH_2-C\underline{H_2}-NH_3^+)_2$, 8H), 2.758 (br, $AdNH-CO-CH_2-NH^+(-CH_2-CH_2-C\underline{H_2}-NH-CO-CH(NH_3^+)-CH_2-CH_2-CH_2-CH_2-NH_3^+)_2$, 4H), 1.823-1.518 (br, Ad*H* and $AdNH-CO-CH_2-NH^+(-CH_2-C\underline{H_2}-CH_2-NH-CO-CH(NH_3^+)-C\underline{H_2}-C\underline{H_2}-C\underline{H_2}-CH_2-NH_3^+)_2$, 30H). ^{13}C-NMR (150 MHz, DMSO-d6) δ/ppm:170.8, 168.6, 158.8, 158.6, 158.4, 158.2, 120.1, 118.2, 116.2, 114.2, 61.4, 52.3, 52.1, 39.5, 38.4, 37.0, 36.8, 36.7, 36.7, 36.2, 31.5, 31.4, 31.2, 30.9, 30.5, 26.7, 26.6, 26.5, 21.3. HRMS (m/z): 579.4681 [$(M+H)^+$] (Observed), 579.4710 [$(M+H)^+$] (Calculated).

Synthesis of adjuvants in Scheme S2

Synthesis of *N^1*-Boc-*N^6*-(6-(Boc-amino)-hexyl) hexane-1,6-diamine (2a): About 2 g (1 equivalent, 9.29 mmol) of Bis(hexamethylene) triamine was dissolved in about 20 mL of

methanol and the solution was kept at -80 °C. Then, 3.04 g (1.5 equivalents, 3.93 mmol) of di-tert-butyl dicarbonate (Boc_2O) was dissolved in MeOH (30 mL) and added to the reaction mixture dropwise. The reaction continued for 1h at −80 °C. Then the reaction mixture was allowed to come to room temperature. MeOH was removed under reduced pressure and purification was done through column chromatography on silica gel (60−120 mesh) using methanol and chloroform as eluent to a□ord the product with 68% yield. ^{1}H-NMR (400 MHz, $CDCl_3$) δ/ppm: 4.541 (s, $NH(-(CH_2)_6\text{-}\underline{\mathit{NH}}Boc)_2$, 2H), 3.112-3.065 (m, $NH(-(CH_2)_5\text{-}\underline{\mathit{CH_2}}\text{-}NHBoc)_2$, 4H), 2.602-2.565 (t, J = 7.2 Hz, $NH(\text{-}\underline{\mathit{CH_2}}\text{-}(CH_2)_5\text{-}NHBoc)_2$, 4H), 1.930 (s, $\underline{\mathit{NH}}(-(CH_2)_6\text{-}NHBoc)_2$, 1H), 1.507-1.296 (m, $NH(-CH_2\text{-}\mathit{(C\underline{H_2})_4}\text{-}CH_2\text{-}NH\text{-}COO\text{-}C\mathit{(C\underline{H_3})_3})_2$, 34H). HRMS (m/z): 416.3419 [(M+H) $^+$] (Observed), 416.3488 [(M+H) $^+$] (Calculated).

Synthesis of *N,N*-bis-[(6-Boc-amino) hexyl] naphthyl ethanamide (2b): 268.8 mg (1.2 equivalent, 1.44 mmol) of Naphthyl acetic acid was dissolved in dry DCM (4 mL) at 0 °C. In the reaction mixture, 628.6 µL (3 equivalents, 3.609 mmol) of *N,N*-diisopropylethylamine (DIPEA) was added followed by 547.5 mg (1.2 equivalents, 1.44 mmol) of *N,N,N',N'*-tetramethyl-O-(1H-benzotriazol-1-yl)uronium hexafluorophosphate (HBTU). Then DMF (2 mL) was added to the reaction mixture to dissolve the HBTU. After 15 min, 500 mg (1 equivalent, 1.2 mmol) of N^1-Boc-N^6-(6-(boc-amino)-hexyl) hexane-1,6-diamine (**2a),** dissolved in dry DCM (4 mL) was added dropwise to the reaction mixture. The reaction mixture was brought to room temperature and allowed to stir for 24 h. Solvent was evaporated, and residue was diluted in ethyl acetate (50 mL). Then workup was carried out first with 1 N HCl (50 mL, 3 times) followed by saturated Na_2CO_3 solution (50 mL, 3 times). The crude product was extracted in ethyl acetate layer. Finally, purification was accomplished through column chromatography on silica gel (60−120 mesh) using di□erent ratios of methanol and chloroform as eluent to a□ord compound **2b** with 68% yield. ^{1}H-NMR (400 MHz, $CDCl_3$) δ/ppm: 7.541-7.527 (m, Ar$\underline{\mathit{H}}$, 1H), 7.524-7.520 (m, Ar$\underline{\mathit{H}}$, 1H), 7.507-7.503 (m, Ar$\underline{\mathit{H}}$, 1H), 7.500-7.329 (m, Ar$\underline{\mathit{H}}$, 4H), 4.417 (s, $R\text{-}CO\text{-}N(-(CH_2)_6\text{-}\underline{\mathit{NH}}Boc)_2$, 2H), 4.115 (s, $Ar\underline{\mathit{CH_2}}CO\text{-}\ N(-(CH_2)_6\text{-}NHBoc)_2$, 2H), 3.363-3.037 (m, $R\text{-}CO\text{-}N(\text{-}\underline{\mathit{CH_2}}\text{-}(CH_2)_4\text{-}\underline{\mathit{CH_2}}\text{-}NHBoc)_2$, 8H), 1.743 (br, $R\text{-}CO\text{-}N(-CH_2\text{-}\underline{\mathit{CH_2}}\text{-}(CH_2)_4\text{-}NHBoc)_2$, 4H), 1.593-1.253 (m, $R\text{-}CO\text{-}N(-CH_2\text{-}(CH_2)_3\text{-}\underline{\mathit{CH_2}}\text{-}\underline{\mathit{CH_2}}\text{-}NH\text{-}COO\text{-}C\mathit{(C\underline{H_3})_3})_2$, 22H), 1.187-1.171 (m, $R\text{-}CO\text{-}N(-CH_2\text{-}CH_2\text{-}\mathit{(C\underline{H_2})_2}\text{-}CH_2\text{-}CH_2\text{-}NHBoc)_2$, 8H). HRMS (m/z): 584.4106 [(M+H) $^+$] (Observed), 584.40635 [(M+H) $^+$] (Calculated).

Synthesis of *N,N*-bis-[(6-Boc-amino) hexyl] 3,3-diphenyl propanamide (2c): 326.6 mg (1.2 equivalents, 1.44 mmol) of 3,3-Diphenyl propionic acid was dissolved in dry DCM (4 mL) at 0 °C. In the reaction mixture, 628.65 µL (3 equivalents, 3.609 mmol) of *N,N*-

diisopropylethylamine (DIPEA) was added followed by 547.48 mg (1.2 equivalents, 1.44 mmol) of *N,N,N',N'*-tetramethyl-O-(1H-benzotriazol-1-yl)uronium hexafluorophosphate (HBTU). Then DMF (2 mL) was added to the reaction mixture to dissolve the HBTU. After 15 min, 500 mg (1 equivalent, 1.203 mmol) of N^1-Boc-N^6-(6-(boc-amino)-hexyl) hexane-1,6-diamine (**2a),** dissolved in dry DCM (4 mL) was added dropwise to the reaction mixture. The reaction mixture was brought to room temperature and allowed to stir for 24 h. Solvent was evaporated, and residue was diluted in ethyl acetate (50 mL). Then workup was carried out first with 1 N HCl (50 mL, 3 times) followed by saturated Na_2CO_3 solution (50 mL, 3 times). The crude product was extracted in ethyl acetate layer. Finally, purification was accomplished through column chromatography on silica gel (60−120 mesh) using di□erent ratios of methanol and chloroform as eluent to a□ord compounds **2c** with 68% yield. ^{1}H-NMR (400 MHz, $CDCl_3$) δ/ppm: 7.282 (s, Ar$\underline{H}$, 1H), 7.245-7.147 (m, Ar$\underline{H}$, 9H), 4.722 (t, J = 7.6 Hz, Ar$C\underline{H}$$CH_2$CO-N(-$(CH_2)_6$-NHBoc$)_2$, 1H), 4.522 (br, R-CO-N(-$(CH_2)_6$-N$\underline{H}$Boc$)_2$, 2H), 3.227-3.059 (m, R-CO-N($C\underline{H}_2$-$(CH_2)_4$-$C\underline{H}_2$-NHBoc$)_2$, 8H), 3.003-2.984 (d, J = 7.6 Hz, ArCH$C\underline{H}_2$CO-N(-$(CH_2)_6$-NHBoc$)_2$, 2H), 1.442 (s, R-CO-N(-$(CH_2)_6$-NH-COO-C$(C\underline{H}_3)_3$$)_2$, 18H), 1.406-1.101 (m, R-CO-N(-CH_2-$(C\underline{H}_2)_4$-CH_2-NHBoc$)_2$,16H). HRMS (m/z): 624.4347 [$(M+H)^+$] (Observed), 624.4376 [$(M+H)^+$] (Calculated).

Synthesis of *N*-(adamantan-2-yl)-2-[*N′,N′*-{bis-(6-Boc-amino) hexyl} amino] ethanamide (2d): 300 mg of N^1-Boc-N^6-(6-(Boc-amino)-hexyl) hexane-1,6-diamine, **2a** (1 equivalent, 0.72 mmol) was dissolved in DMF. 149.6 mg of K_2CO_3 (1.5 equivalents, 1.1 mmol) was added to the reaction mixture. Then, 196.5 mg of *N*-(adamantan-2-yl)-2-bromoethanamide, *N*-(adamantan-2-yl)-2-bromoethanamide (1 equivalent, 0.72 mmol), dissolved in DMF was added to the reaction mixture. The reaction was stirred at room temperature for 24 hours. The reaction mixture was then dissolved in ethyl acetate and work-up was performed with ice-cold water (3 times) to remove the DMF. The ethyl acetate layer was collected, and solvent evaporated to get the pure product with 61% yield. ^{1}H-NMR (400 MHz, $CDCl_3$) δ/ppm: 8.575 (br, Ad-N$\underline{H}$-CO-CH_2-N(-$(CH_2)_6$-NHBoc$)_2$, 1H), 4.660-4.489 (br, Ad-NH-CO-CH_2-N(-$(CH_2)_6$-N$\underline{H}$Boc$)_2$, 2H), 4.145-4.027 (m, Ad$\underline{H}$, 1H), 3.103 (br, Ad-NH-CO-$C\underline{H}_2$-N(-$C\underline{H}_2$-$(CH_2)_4$-$C\underline{H}_2$-NHBoc$)_2$, 6H), 2.041-1.594 (m, Ad$\underline{H}$ and -$C\underline{H}_2$-(Boc-aminohexyl) ,26H), 1.440 (s, Ad-NH-CO-CH_2-N(-$(CH_2)_6$-NH-COO-C$(C\underline{H}_3)_3$$)_2$, 18H), 1.375-1.238 (m, Ad$\underline{H}$ and -$C\underline{H}_2$-(Boc-aminohexyl) ,8H). HRMS (m/z): 607.4764 [$(M+H)^+$] (Observed), 607.4798 [$(M+H)^+$] (Calculated).

General procedure for synthesizing final compounds (BNaph, BDiphe and BAda): At first 1 equivalent of **2b-2d** was dissolved in dry DCM. To the intensely stirred solution 4 equivalents

(excess amount) of trifluoroacetic acid (TFA) was added and allowed to stir at RT for 4h. After that reaction, solvent and unused TFA was removed to get the pure product with 100% yield with CF_3COO^- counterions.

***N,N*-bis-[(6-amino) hexyl] naphthyl ethanamide bistrifluoroacetate (BNaph, 7):** ^{1}H-NMR (400 MHz, DMSO-d_6) δ/ppm: 7.921-7.707 (m, Ar$\underline{H}$ and R-CO-N-($(CH_2)_6$-N$\underline{H_3}^+)_2$), 9H), 7.538-7.426 (m, Ar$\underline{H}$, 3H), 7.343-7.326 (m, Ar$\underline{H}$, 1H), 4.117 (s, Ar$\underline{CH_2}$CO- N(-$(CH_2)_6$-$NH_3^+)_2$, 2H), 3.258 (t, J = 7.2 Hz, R-CO-N($(CH_2)_5$-$\underline{CH_2}$-$NH_3^+)_2$, 4H), 2.772-2.722 (m, R-CO-N-($\underline{CH_2}$-$(CH_2)_5$-$NH_3^+)_2$, 4H), 1.536-1.440 (m, R-CO-N-(CH_2-$\underline{CH_2}$-$(CH_2)_2$-$\underline{CH_2}$-CH_2-$NH_3^+)_2$, 8H), 1.335-1.220 (m, R-CO-N-(CH_2-CH_2-$(\underline{CH_2})_2$-CH_2-CH_2-$NH_3^+)_2$, 8H). ^{13}C-NMR (100 MHz, DMSO-d6) δ/ppm: 169.7, 158.8, 158.5, 158.2, 157.8, 133.3, 132.9, 132.0, 128.4, 127.0, 125.9, 125.6, 125.4, 124.1, 118.7, 115.7, 112.7, 47.5, 45.0, 39.5, 38.7, 37.4, 28.4, 27.0, 26.9, 26.0, 25.7, 25.6. HRMS (m/z): 384.3040 [$(M+H)^+$] (Observed), 384.3015 [$(M+H)^+$] (Calculated).

***N,N*-bis-[(6-amino) hexyl] 3,3-diphenyl propanamide bistrifluoroacetate (BDiphe, 8):** ^{1}H-NMR (400 MHz, DMSO-d_6) δ/ppm: 7.735 (bs, R-CO-N-($(CH_2)_6$-N$\underline{H_3}^+)_2$, 6H), 7.296-7.097 (m, Ar$\underline{H}$, 10H), 4.522 (t, J = 7.2 Hz, Ar$\underline{CH}$$CH_2$CO-N(-$(CH_2)_6$-$NH_3^+)_2$, 1H), 3.241-3.109 (m, R-CO-N(CH_2-$(CH_2)_4$-$\underline{CH_2}$-$NH_3^+)_2$, 4H), 3.036-3.018 (d, J = 7.2 Hz, ArCH$\underline{CH_2}$CO-N(-$(CH_2)_6$-$NH_3^+)_2$, 2H), 2.788-2.701 (m, R-CO-N($\underline{CH_2}$-$(CH_2)_4$-CH_2-$NH_3^+)_2$, 4H), 1.532-1.077 (m, R-CO-N(CH_2-$(\underline{CH_2})_4$-CH_2-$NH_3^+)_2$, 16H). ^{13}C-NMR (100 MHz, DMSO-d6) δ/ppm: 169.6, 158.7, 158.4, 158.1, 157.8, 144.7, 128.2, 127.7, 127.6, 126.0, 118.7, 115.7, 47.0, 46.9, 44.8, 39.5, 38.7, 37.8, 28.6, 27.0, 25.7, 25.6, 25.6. HRMS (m/z): 424.3299 [$(M+H)^+$] (Observed), 424.3328 [$(M+H)^+$] (Calculated).

***N*-(adamantan-2-yl)-2-[*N´,N´*-{bis-(6-amino) hexyl} amino] ethanamide tristrifluoroacetate (BAda, 9):** ^{1}H-NMR (400 MHz, DMSO-d_6) δ/ppm: 8.540-8.457 (br, Ad-N$\underline{H}$-CO-CH_2-NH^+(-$(CH_2)_6$-$NH_3^+)_2$, 1H), 7.800 (br, Ad-NH-CO-CH_2-NH^+(-$(CH_2)_6$-N$\underline{H_3}^+)_2$, 6H), 3.902-3.820 (m, Ad$\underline{H}$ and Ad-NH-CO-$\underline{CH_2}$-NH^+(-$(CH_2)_6$-$NH_3^+)_2$, 3H), 3.082-2.669 (br, Ad-NH-CO-CH_2-NH^+(-$\underline{CH_2}$-$(CH_2)_4$-$\underline{CH_2}$-$NH_3^+)_2$, 8H), 1.967-1.235 (m, Ad$\underline{H}$ and -$\underline{CH_2}$-$_{(aminohexyl)}$, 30H). ^{13}C-NMR (100 MHz, DMSO-d6) δ/ppm: 170.8, 162.6, 158.9, 158.6, 158.3, 158.0, 118.6, 115.7, 61.4, 53.9, 53.5, 52.1, 46.6, 39.5, 38.6, 38.6, 38.5, 37.0, 36.7, 36.6, 31.5, 31.4, 31.3, 31.1, 30.9, 26.8, 26.7, 26.6, 25.5, 25.4, 25.3. HRMS (m/z): 407.3724 [$(M+H)^+$] (Observed), 407.3749 [$(M+H)^+$] (Calculated).

Synthesis of adjuvants in Scheme S3 and S4

Synthesis of N^1-(Boc-^{L}Lys)-N^6-[{6-(Boc-^{L}Lys) amido} hexyl] hexane-1,6-diamine (3a): About 6.4 g (2 equivalents, 18.56 mmol) of *N, N′*-DiBoc-^{L}Lysine was dissolved in about 25 mL of dry DCM at 0 °C. In the reaction mixture about 6.5 mL (4 equivalents, 37.1 mmol) of DIPEA was added followed by about 7.0 g (2 equivalents, 18.6 mmol) of HBTU. Now about 8 mL of DMF was added to the reaction mixture. After 10 minutes, about 2 g (1 equivalent, 9.3 mmol) of Bis(hexamethylene) triamine, dissolved in 10 mL of DCM was added drop wise to the reaction mixture. The reaction mixture was allowed to stir for 48 h at 0°C. Then reaction solvent was evaporated, and residue was diluted in ethyl acetate. Thereafter work-up was done with 1N HCl (50 mL, 3 times) followed by saturated Na_2CO_3 solution (50 mL, 3 times). The crude product was collected in ethyl acetate layer. Finally, column was done using different ratios of chloroform and methanol solution, to isolate the product with 34-42% yield. ^{1}H-NMR (400 MHz, $CDCl_3$) δ/ppm: 6.489-6.323 (m, NH(-$(CH_2)_6$-N$\underline{H}$-CO-CH(NHBoc)-$(CH_2)_4$-NHBoc)$_2$, 2H), 5.296 (br, NH(-$(CH_2)_6$-NH-CO-CH(N$\underline{H}$Boc)$(CH_2)_4$-NHBoc)$_2$, 2H), 4.705 (br, NH(-$(CH_2)_6$-NH-CO-CH(NHBoc)($(CH_2)_4$-N$\underline{H}$Boc)$_2$, 2H), 4.020 (br, NH(-$(CH_2)_6$-NH-CO-C$\underline{H}$(NHBoc)$(CH_2)_4$-NHBoc)$_2$, 2H), 3.246-3.075 (m, NH(-$(CH_2)_5$-$C\underline{H}_2$-NH-CO-CH(NHBoc)$(CH_2)_3$-$C\underline{H}_2$-NHBoc)$_2$, 8H), 2.729 (br, NH(-$C\underline{H}_2$-$(CH_2)_5$-NH-CO-CH(NHBoc)$(CH_2)_4$-NHBoc)$_2$, 4H), 1.836-1.480 (m, NH(-CH_2-$C\underline{H}_2$-$(CH_2)_2$-$C\underline{H}_2$-CH_2-NH-CO-CH(NHBoc)(-$C\underline{H}_2$-CH_2-$C\underline{H}_2$-CH_2-NHBoc)$_2$, 16H), 1.431 (s, NH(-$(CH_2)_6$-NH-CO-CH(NH-COO-C($(C\underline{H}_3)_3$)$(CH_2)_4$-NH-COO-C$(C\underline{H}_3)_3$)$_2$, 36H), 1.382-1.347 (m, NH(-CH_2-CH_2-$(C\underline{H}_2)_2$-CH_2-CH_2-NH-CO-CH(NHBoc)(-CH_2-$C\underline{H}_2$-CH_2-CH_2-NHBoc)$_2$, 12H). HRMS (m/z): 872.6286 [$(M+H)^+$] (Observed), 872.6436 [$(M+H)^+$] (Calculated).

Synthesis of N^1-(Boc-^{D}Lys)-N^6-[{6-(Boc-^{D}Lys) amido} hexyl] hexane-1,6-diamine (3a′): Similar protocol as **3a** was followed. Only *N, N′*-DiBoc-^{D}Lysine was used for the conjugation. This product was taken forward for synthesizing D-LBDiphe. ^{1}H-NMR (400 MHz, $CDCl_3$) δ/ppm: 6.416 (br, NH(-$(CH_2)_6$-N$\underline{H}$-CO-CH(NHBoc)-$(CH_2)_4$-NHBoc)$_2$, 2H), 5.607-5.281 (br, NH(-$(CH_2)_6$-NH-CO-CH(N$\underline{H}$Boc)$(CH_2)_4$-NHBoc)$_2$, 2H), 4.754-4.694 (br, NH(-$(CH_2)_6$-NH-CO-CH(NHBoc)($(CH_2)_4$-N$\underline{H}$Boc)$_2$, 2H), 4.020 (t, NH(-$(CH_2)_6$-NH-CO-C$\underline{H}$(NHBoc)$(CH_2)_4$-NHBoc)$_2$, 2H), 3.230-3.090 (m, NH(-$(CH_2)_5$-$C\underline{H}_2$-NH-CO-CH(NHBoc)$(CH_2)_3$-$C\underline{H}_2$-NHBoc)$_2$, 8H), 2.638-2.361 (m, NH(-$C\underline{H}_2$-$(CH_2)_5$-NH-CO-CH(NHBoc)$(CH_2)_4$-NHBoc)$_2$, 4H), 1.834-1.476 (m, NH(-CH_2-$C\underline{H}_2$-$(CH_2)_2$-$C\underline{H}_2$-CH_2-NH-CO-CH(NHBoc)(-$C\underline{H}_2$-CH_2-$C\underline{H}_2$-CH_2-NHBoc)$_2$, 16H), 1.431 (s, NH(-$(CH_2)_6$-NH-CO-CH(NH-COO-C($(C\underline{H}_3)_3$)$(CH_2)_4$-NH-COO-C$(C\underline{H}_3)_3$)$_2$, 36H), 1.342-1.251 (m, NH(-CH_2-CH_2-$(C\underline{H}_2)_2$-CH_2-CH_2-NH-CO-CH(NHBoc)(-

CH_2-$C\underline{H}_2$-CH_2-CH_2-NHBoc$)_2$, 12H). HRMS (m/z): 872.6443 [(M+H) $^+$] (Observed), 872.6436 [(M+H) $^+$] (Calculated).

Synthesis of *N,N*-bis-[{6-(Boc-^{L}Lys)amido} hexyl] naphthyl ethanamide (3b): 41 mg (1.2 equivalents, 0.22 mmol) of Naphthyl acetic acid was dissolved in dry DCM (4 mL) at 0 °C. In the reaction mixture, 96 µL (3 equivalents, 0.55 mmol) of *N,N*-diisopropylethylamine (DIPEA) was added followed by 83.5 mg (1.2 equivalents, 0.22 mmol) of *N,N,N',N'*-tetramethyl-O-(1H-benzotriazol-1-yl)uronium hexafluorophosphate (HBTU). Then DMF (2 mL) was added to the reaction mixture to dissolve the HBTU. After 15 min, 160 mg (1 equivalent, 0.18 mmol) of N^1-(Boc-^{L}Lys)-N^6-[{6-(Boc-^{L}Lys) amido} hexyl] hexane-1,6-diamine, **3a** dissolved in dry DCM (4 mL) was added dropwise to the reaction mixture. The reaction mixture was brought to room temperature and allowed to stir for 24 h. Solvent was evaporated, and residue was diluted in ethyl acetate (50 mL). Then workup was carried out first with 1 N HCl (50 mL, 3 times) followed by saturated Na_2CO_3 solution (50 mL, 3 times). The crude product was extracted in ethyl acetate layer. Finally, purification was accomplished through column chromatography on silica gel (60−120 mesh) using di□erent ratios of methanol and chloroform as eluent to a□ord compound **3b** with 47% yield. ^{1}H-NMR (400 MHz, $CDCl_3$) δ/ppm: 7.949-7.929 (m, Ar$\underline{H}$, 1H), 7.873-7.854 (m, Ar$\underline{H}$, 1H), 7.850-7.777 (m, Ar$\underline{H}$, 1H), 7.757-7.469 (m, Ar$\underline{H}$, 2H), 7.432-7.393 (m, Ar$\underline{H}$, 1H), 7.339-7.322 (m, Ar$\underline{H}$, 1H), 6.472-6.495 (br, R-CO-N(-$(CH_2)_6$-N$\underline{H}$-CO-^{L}Lys(Boc$)_2)_2$, 2H), 5.289-5.180 (br, R-CO-N(-$(CH_2)_6$-NH-CO-CH(N$\underline{H}$Boc)$(CH_2)_4$-NHBoc$)_2$, 2H), 4.637 (br, R-CO-N(-$(CH_2)_6$-NH-CO-CH(NHBoc)$(CH_2)_4$-N$\underline{H}$Boc$)_2$, 2H), 4.123 (s, Ar-$C\underline{H}_2$-CO- N(-$(CH_2)_6$-NH-CO-CH(NHBoc)$(CH_2)_4$-NHBoc$)_2$, 2H), 4.005 (br, R-CO-N(-$(CH_2)_6$-NH-CO-$C\underline{H}$(NHBoc)$(CH_2)_4$-NHBoc$)_2$, 2H), 3.375-3.079 (m, R-CO-N(-$C\underline{H}_2$-$(CH_2)_4$-$C\underline{H}_2$-NH-CO-CH(NHBoc)$(CH_2)_3$-$C\underline{H}_2$-NHBoc$)_2$, 12H), 1.717-1.538 (m, -CH_2-, 20H), 1.433 (s, R-CO-N($(CH_2)_6$-NH-CO-CH(NH-COO-C$(C\underline{H}_3)_3$)$(CH_2)_4$-NH-COO-C$(C\underline{H}_3)_3)_2$, 36H), 1.346-1.219 (m, -CH_2-, 8H). HRMS (m/z): 1040.6816 [(M+H)$^+$] (Observed), 1040.7011 [(M+H)$^+$] (Calculated).

Synthesis of *N,N*-bis-[{6-(Boc-^{L}Lys)amido} hexyl] 3,3 diphenyl propanamide (3c): 233 mg (1.2 equivalents, 1.0 mmol) of 3,3-Diphenyl propionic acid was dissolved in dry DCM (4 mL) at 0 °C. In the reaction mixture, 448.9 µL (3 equivalents, 2.6 mmol) of *N,N*-diisopropylethylamine (DIPEA) was added followed by 390.63 mg (1.2 equivalents, 1.0 mmol) of *N,N,N',N'*-tetramethyl-O-(1H-benzotriazol-1-yl)uronium hexafluorophosphate (HBTU). Then DMF (2 mL) was added to the reaction mixture to dissolve the HBTU. After 15 min, 750 mg (1 equivalent, 0.859) of N^1-(Boc-^{L}Lys)-N^6-[{6-(Boc-^{L}Lys) amino} hexyl] hexane-1,6-

diamine, **3a** dissolved in dry DCM (4 mL) was added dropwise to the reaction mixture. The reaction mixture was brought to room temperature and allowed to stir for 24 h. Solvent was evaporated, and residue was diluted in ethyl acetate (50 mL). Then workup was carried out first with 1 N HCl (50 mL, 3 times) followed by saturated Na_2CO_3 solution (50 mL, 3 times). The crude product was extracted in ethyl acetate layer. Finally, purification was accomplished through column chromatography on silica gel (60−120 mesh) using di□erent ratios of methanol and chloroform as eluent to a□ord compound **3c** with 59% yield. ^{1}H-NMR (400 MHz, $CDCl_3$) δ/ppm: 7.275 (s, Ar*H*, 2H), 7.240-7.147 (m, Ar*H*, 8H), 6.463-6.311 (br, R-CO-N(-$(CH_2)_6$-N*H*-CO-CH(NHBoc)$(CH_2)_4$-NHBoc)$_2$, 2H), 5.310-5.159 (br, R-CO-N(-$(CH_2)_6$-NH-CO-CH(N*H*Boc)$(CH_2)_4$-NHBoc)$_2$, 2H), 4.717 (t, *J* = 7.2 Hz, Ar-*CH*-CH_2-N(-$(CH_2)_6$-NH-CO-CH(NHBoc)$(CH_2)_4$-NHBoc)$_2$, 1H), 4.640 (br, R-CO-N(-$(CH_2)_6$-NH-CO-C*H*(NHBoc)$(CH_2)_4$-NHBoc)$_2$, 2H), 4.108-3.995 (br, R-CO-N(-$(CH_2)_6$-NH-CO-CH(NHBoc)$(CH_2)_4$-N*H*Boc)$_2$, 2H), 3.232-3.088 (m, R-CO-N(-*CH_2*-$(CH_2)_4$-*CH_2*-NH-CO-CH(NHBoc)$(CH_2)_3$-*CH_2*-N*H*Boc)$_2$, 12H), 3.013-2.994 (d, *J* = 7.6 Hz, Ar-CH-*CH_2*-N(-$(CH_2)_6$-NH-CO-CH(NHBoc)$(CH_2)_4$-NHBoc)$_2$, 2H), 1.823-1.561 (m, -*CH_2*-, 12H), 1.433 (s, R-CO-N($(CH_2)_6$-NH-CO-CH(NH-COO-C*$(CH_3)_3$*)$(CH_2)_4$-NH-COO-C*$(CH_3)_3$*)$_2$, 36H), 1.392-1.097 (m, -*CH_2*-, 16H). HRMS (m/z): 1080.7631 [$(M+H)^+$] (Observed), 1080.7324 [$(M+H)^+$] (Calculated).

Synthesis of *N,N*-bis-[{6-(Boc-^{D}Lys)amido} hexyl] 3,3 diphenyl propanamide (3c′): Similar protocol as **3c** was followed to synthesize but only bis(hexamethylene)-triamine conjugated with *N*, *N′*-DiBoc ^{D}Lysine was taken as the starting material. ^{1}H-NMR (400 MHz, $CDCl_3$) δ/ppm: 7.277-7.147 (m, Ar*H*, 10H), 6.447-6.303 (br, R-CO-N(-$(CH_2)_6$-N*H*-CO-CH(NHBoc)$(CH_2)_4$-NHBoc)$_2$, 2H), 5.324-5.179 (br, R-CO-N(-$(CH_2)_6$-NH-CO-CH(N*H*Boc)$(CH_2)_4$-NHBoc)$_2$, 2H), 4.736-4.636 (m, Ar-*CH*-CH_2-N(-$(CH_2)_6$-NH-CO-CH(NHBoc)$(CH_2)_4$-N*H*Boc)$_2$, 3H), 4.059-3.996 (m, R-CO-N(-$(CH_2)_6$-NH-CO-C*H*(NHBoc)$(CH_2)_4$-NHBoc)$_2$, 2H), 3.221-3.089 (m, R-CO-N(-*CH_2*-$(CH_2)_4$-*CH_2*-NH-CO-CH(NHBoc)$(CH_2)_3$-*CH_2*-N*H*Boc)$_2$, 12H), 3.011-2.993 (d, *J* = 7.2 Hz, Ar-CH-*CH_2*-N(-$(CH_2)_6$-NH-CO-CH(NHBoc)$(CH_2)_4$-NHBoc)$_2$, 2H), 1.858-1.563 (m, -*CH_2*-, 12H), 1.433 (s, R-CO-N($(CH_2)_6$-NH-CO-CH(NH-COO-C*$(CH_3)_3$*)$(CH_2)_4$-NH-COO-C*$(CH_3)_3$*)$_2$, 36H), 1.398-1.097 (m, -*CH_2*-, 16H). HRMS (m/z): 1080.7292 [$(M+H)^+$] (Observed), 1080.7324 [$(M+H)^+$] (Calculated).

Synthesis of *N*-(adamantan-2-yl)-2-[*N′,N′*-{bis-(6-(Boc-^{L}Lys)amido)hexyl} amino] ethanamide (3d): 300 mg of *N^1*-(Boc-^{L}Lys)-*N^6*-[{6-(boc-^{L}Lys)amido} hexyl] hexane-1,6-diamine, **3a** (1 equivalent, 0.344 mmol) in DMF. 71.29 mg of K_2CO_3 (1.5 equivalents, 0.52

mmol) was added to the reaction mixture. Then, 93.60 mg of *N*-(adamantan-2-yl)-2-bromoethanamide (1 equivalent, 0.34 mmol), dissolved in DMF was added to the reaction mixture. The reaction was stopped after 24 hours. The reaction mixture was then dissolved in ethyl acetate and work-up was performed with ice-cold water (3 times) to remove the DMF. The ethyl acetate layer was collected, and solvent evaporated to get the pure product with 52% yield. ^{1}H-NMR (400 MHz, CDCl$_3$) δ/ppm: 6.517 (br, Ad-NH-CO-CH$_2$-N(-(CH$_2$)$_6$-N*H*-CO-CH(NHBoc)(CH$_2$)$_4$-NHBoc)$_2$, 2H), 5.2913 (br, Ad-NH-CO-CH$_2$-N(-(CH$_2$)$_6$-NH-CO-CH(N*H*Boc)(CH$_2$)$_4$-NHBoc)$_2$, 2H), 4.699 (br, Ad-NH-CO-CH$_2$-N(-(CH$_2$)$_6$-NH-CO-CH(NHBoc)(CH$_2$)$_4$-N*H*Boc)$_2$, 2H), 4.144-4.033 (m, Ad*H* and Ad-NH-CO-CH$_2$-N(-(CH$_2$)$_6$-NH-CO-*CH*(NHBoc)(CH$_2$)$_4$-N*H*Boc)$_2$, 3H), 3.227-3.090 (br, Ad-NH-CO-*CH$_2$*-N(-(CH$_2$)$_5$-*CH$_2$*-NH-CO-CH(NHBoc)(CH$_2$)$_3$-*CH$_2$*-N*H*Boc)$_2$, 10H), 2.039-1513 (m, Ad*H*, -C*H$_2$*-$_{\text{(Boc-Lys) amidohexyl}}$ and -C*H$_2$*-$_{\text{(Lys)}}$, 34H), 1.430 (s, Ad-NH-CO-CH$_2$-N(-(CH$_2$)$_5$-CH$_2$-NH-CO-CH(NH-COO-C*(CH$_3$)$_3$*)(CH$_2$)$_3$-CH$_2$-NH-COO-C*(CH$_3$)$_3$*)$_2$, 36H), 1.357-1.236 (m, Ad*H*, -C*H$_2$*-$_{\text{(Boc-Lys) amidohexyl}}$ and -C*H$_2$*-$_{\text{(Lys)}}$, 12H). HRMS (m/z): 1063.7691 [(M+H)$^+$] (Observed), 1063.7746 [(M+H)$^+$] (Calculated).

General procedure for synthesizing final compounds (LBNaph, LBDiphe, LBAda and D-LBDiphe): At first 1 equivalent of **3b-3d** was dissolved in dry DCM. To the intensely stirred solution 4 equivalents (excess amount) of trifluoroacetic acid (TFA) was added and allowed to stir at RT for 4h. After the reaction, solvent and unused TFA was removed to get the pure product with 100% yield with CF$_3$COO$^-$ counterions.

For D-LBDiphe, the Boc-protected D-LBDiphe was dissolved in methanol. To the intensely stirred solution, 4N HCl was added until the turbidity persisted in the solution. The final ratio of 4N HCl and methanol was 2:1. The reaction was stirred at room temperature for 4 h. After the reaction, MeOH and HCl were evaporated and the solution lyophilized to get the pure product, D-LBDiphe with quantitative yield and Cl$^-$ counterions.

***N,N*-bis-[(6-(^{L}Lys)amido) hexyl] naphthyl ethanamide tetrakistrifluoroacetate (LBNaph, 10):** ^{1}H-NMR (400 MHz, DMSO-d$_6$) δ/ppm: 8.408-7.722 (m, Ar*H* and R-CO-N(-(CH$_2$)$_6$-N*H*-CO-CH(N*H$_3$*$^+$)(CH$_2$)$_4$-N*H$_3$*$^+$)$_2$, 18H), 7.527-7.324 (m, Ar*H*, 3H), 4.117 (s, Ar*CH$_2$*CO-N-(-(CH$_2$)$_6$-NH-CO-CH(NH$_3^+$)(CH$_2$)$_4$-NH$_3^+$)$_2$, 2H), 3.665 (br, R-CO-N(-(CH$_2$)$_6$-NH-CO-*CH*(NH$_3^+$)(CH$_2$)$_4$-NH$_3^+$)$_2$, 2H), 3.273-3.077 (m, R-CO-N(-(CH$_2$)$_5$-*CH$_2$*-NH-CO-CH(NH$_3^+$)(CH$_2$)$_3$-*CH$_2$*-NH$_3^+$)$_2$, 8H), 2.737 (br, R-CO-N(-*CH$_2$*-(CH$_2$)$_5$-NH-CO-CH(NH$_3^+$)(CH$_2$)$_3$-CH$_2$-NH$_3^+$)$_2$, 4H), 1.689-1.652 (m, R-CO-N(-(CH$_2$)$_6$-NH-CO-

CH(NH$_3^+$)(*C*$\underline{H_2}$-(CH$_2$)$_3$- NH$_3^+$)$_2$, 4H), 1.519-1.235 (m, R-CO-N(-CH$_2$-*(C*$\underline{H_2}$*)$_4$*-CH$_2$-NH-CO-CH(NH$_3^+$)(CH$_2$-*(C*$\underline{H_2}$*)$_2$*-CH$_2$-NH$_3^+$)$_2$, 24H). ^{13}C-NMR (100 MHz, DMSO-d6) δ/ppm: 169.7, 168.2, 158.4, 158.1, 155.9, 133.3, 132.0, 127.0, 125.9, 125.6, 125.4, 118.7, 115.7, 52.0, 39.5, 38.7, 38.4, 30.5, 28.8, 27.1, 26.5, 26.1, 21.2. HRMS (m/z): 640.4956 [(M+H)$^+$] (Observed), 640.4914 [(M+H)$^+$] (Calculated).

***N,N*-bis-[(6-(^{L}Lys)amido) hexyl] 3,3-diphenyl propanamide tetrakis(trifluoroacetate) (LBDiphe, 11):** ^{1}H-NMR (400 MHz, DMSO-d$_6$) δ/ppm: 8.459- 7.839 (m, R-CO-N(-(CH$_2$)$_6$-NH-CO-CH(N$\underline{H_3}$$^+$)(CH$_2$)$_4$-N$\underline{H_3}$$^+$)$_2$, 12H), 7.292-7.128 (m, Ar$\underline{H}$, 10H), 5.155-5.133 (br, R-CO-N(-(CH$_2$)$_6$-N$\underline{H}$-CO-CH(NH$_3^+$)(CH$_2$)$_4$-NH$_3^+$)$_2$, 2H), 4.516 (t, J = 7.2 Hz, Ar*C*$\underline{H}$CH$_2$-CO- N(-(CH$_2$)$_6$-NH-CO-CH(NH$_3^+$)(CH$_2$)$_4$-NH$_3^+$)$_2$, 1H), 3.698-3.685 (br, R-CO-N(-(CH$_2$)$_6$-NH-CO-*C*$\underline{H}$(NH$_3^+$)(CH$_2$)$_4$-NH$_3^+$)$_2$, 2H), 3.220-2.687 (m, ArCH*C*$\underline{H_2}$-CO-N(-*C*$\underline{H_2}$-(CH$_2$)$_4$-*C*$\underline{H_2}$-NH-CO-CH(NH$_3^+$)(CH$_2$)$_3$-*C*$\underline{H_2}$-NH$_3^+$)$_2$, 14H), 1.696-1.042 (m, R-CO-N(-CH$_2$-*(C*$\underline{H_2}$*)$_4$*-CH$_2$-NH-CO-CH(NH$_3^+$)(*(C*$\underline{H_2}$*)$_3$*-CH$_2$-NH$_3^+$)$_2$, 28H). HRMS (m/z): 680.5191 [(M+H)$^+$] (Observed), 680.5227 [(M+H)$^+$] (Calculated).

***N*-(adamantan-2-yl)-2-[*N′,N′*-{bis-(6-^{L}Lys) amido)hexyl} amino] ethanamide pentakis(trifluoroacetate) (LBAda, 12):** ^{1}H-NMR (400 MHz, DMSO-d$_6$) δ/ppm: 9.509 (br, Ad-N$\underline{H}$-CO-CH$_2$-N(-(CH$_2$)$_6$-NH-CO-CH(NH$_3^+$)(CH$_2$)$_4$-NH$_3^+$)$_2$, 1H), 8.468 (t, J = 4.8 Hz, Ad-NH-CO-CH$_2$-N(-(CH$_2$)$_6$-N$\underline{H}$-CO-CH(NH$_3^+$)(CH$_2$)$_4$-NH$_3^+$)$_2$, 2H), 8.149 (br, -NH$_3^+$, 6H), 7.816 (br, -NH$_3^+$, 6H), 3.975-3.899 (br, Ad-NH-CO-CH$_2$-N(-(CH$_2$)$_6$-NH-CO-*C*$\underline{H}$(NH$_3^+$)(CH$_2$)$_4$-NH$_3^+$)$_2$, 2H), 3.820 (s, Ad-NH-CO-*C*$\underline{H_2}$-N(-(CH$_2$)$_6$-NH-CO-CH(NH$_3^+$)(CH$_2$)$_4$-NH$_3^+$)$_2$, 2H), 3.696-3.685 (br, Ad$\underline{H}$, 1H), 3.096-3.086 (br, Ad-NH-CO-CH$_2$-N(-(CH$_2$)$_5$-*C*$\underline{H_2}$-NH-CO-CH(NH$_3^+$)(CH$_2$)$_3$-*C*$\underline{H_2}$-NH$_3^+$)$_2$, 8H), 2.751 (br, Ad-NH-CO-CH$_2$-N(-*C*$\underline{H_2}$-(CH$_2$)$_5$-NH-CO-CH(NH$_3^+$)(CH$_2$)$_3$-CH$_2$-NH$_3^+$)$_2$, 4H), 1.968-1.235 (m, Ad$\underline{H}$, -C$\underline{H_2}$-$_{\text{(Lys amidohexyl)}}$ and -C$\underline{H_2}$-$_{\text{(Lys)}}$, 42H). ^{13}C-NMR (100 MHz, DMSO-d6) δ/ppm: 168.2, 158.4, 158.1, 118.7, 115.7, 52.0, 39.5, 38.6, 38.4, 37.0, 36.6, 31.5, 31.4, 30.9, 30.5, 28.6, 26.6, 26.4, 26.0, 21.2. HRMS (m/z): 663.5619 [(M+H)$^+$] (Observed), 663.5649 [(M+H)$^+$] (Calculated).

***N,N*-bis-[(6-(^{D}Lys)amido) hexyl] 3,3-diphenyl propanamide tetrakis(chloride) (D-LBDiphe, 13):** HPLC Purity ~ 96%, ^{1}H-NMR (400 MHz, DMSO-d$_6$) δ/ppm: 8.699 (t, R-CO-N(-(CH$_2$)$_6$-N$\underline{H}$-CO-CH(NH$_3^+$)(CH$_2$)$_4$-NH$_3^+$)$_2$, 1H) 8.643 (t, R-CO-N(-(CH$_2$)$_6$-N$\underline{H}$-CO-CH(NH$_3^+$)(CH$_2$)$_4$-NH$_3^+$)$_2$, 1H), 8.310 (br, R-CO-N(-(CH$_2$)$_6$-NH-CO-CH(N$\underline{H_3}$$^+$)(CH$_2$)$_4$-NH$_3^+$)$_2$, 6H), 8.100 (br, R-CO-N(-(CH$_2$)$_6$-NH-CO-CH(NH$_3^+$)(CH$_2$)$_4$-N$\underline{H_3}$$^+$)$_2$, 6H), 7.305-7.129 (m, Ar$\underline{H}$, 10H), 4.518 (t, J = 7.4 Hz, Ar*C*$\underline{H}$CH$_2$-CO- N(-(CH$_2$)$_6$-NH-CO-CH(NH$_3^+$)(CH$_2$)$_4$-

$NH_3^+)_2$, 1H), 3.744 (br, R-CO-N(-$(CH_2)_6$-NH-CO-*C<u>H</u>*(NH_3^+)$(CH_2)_4$-$NH_3^+)_2$, 2H), 3.249-2.713 (m, ArCH*C<u>H</u>$_2$*-CO-N(-*C<u>H</u>$_2$*-$(CH_2)_4$-*C<u>H</u>$_2$*-NH-CO-CH(NH_3^+)$(CH_2)_3$-*C<u>H</u>$_2$*-$NH_3^+)_2$, 14H), 1.760-1.051 (m, R-CO-N(-CH_2-*(C<u>H</u>$_2$)$_4$*-CH_2-NH-CO-CH(NH_3^+)(*(C<u>H</u>$_2$)$_3$*-CH_2-$NH_3^+)_2$, 28H). ^{13}C-NMR (100 MHz, DMSO-d_6) δ/ppm: 144.7, 128.2, 127.7, 126.0, 51.8, 47.0, 44.9, 39.5, 38.6, 38.1, 37.8, 30.3, 28.7, 27.1, 26.1, 25.9, 21.1. HRMS (m/z): 680.5211 [$(M+H)^+$] (Observed), 680.5227 [$(M+H)^+$] (Calculated).

Biological, biophysical and theoretical studies

1. Antibacterial activity: Minimum Inhibitory Concentration (MIC)[2]

Water-soluble small-molecular adjuvants or antibiotics were assayed in a modified micro-dilution broth format. Stock solutions of all the adjuvants and antibiotics (10 mg/mL) were made in autoclaved Millipore water except for rifampicin whose stock solutions were made in DMSO. The dilutions of the adjuvants or antibiotics were done in a 96-well plate using autoclaved millipore water. Bacteria, to be tested, were cultured for 6 h in nutrient broth media. The cultured bacteria contained ~10^8 CFU/mL which was determined by spot plating method. 10^8 CFU/mL bacteria were diluted to 10^5 CFU/mL using Mueller Hinton Broth media; which was also used to perform the experiments. 150 μL of bacterial solutions were added to 50 μL of serially diluted adjuvants/antibiotics present in a 96 well plate (Polystyrene). Two controls were used for all the experiments; one containing 150 μL of media and 50 μL of adjuvant/antibiotic, and, the other containing 150 μL of bacterial solution and 50 μL of water. The plate was then incubated at 37 □ for a period of 18-24 h and the O.D. value was measured at 600 nm using a Tecan InfinitePro series M200 Microplate Reader. MIC value was determined by taking the average of triplicate O.D. values for each concentration and plotting it against concentration using Origin Pro 9.0 software. The data was then subjected to sigmoidal fitting. From the curve the MIC was determined, at the point where the O.D. was similar to that of control having no bacteria. The MIC values are reported as averages of two independent experiments with three technical replicates each time.

2. *In-vitro* hemolytic activity and cytotoxicity

2.1 Hemolytic assay[2]

Human red blood cells (hRBCs) were isolated from freshly drawn, human blood and resuspended to 5% v/v in 1X PBS (pH 7.4). In a 96-well plate, 150 μL of erythrocyte suspension was added to 50 μL of serially diluted compound to give a final solution of 3.75% v/v

erythrocytes. 50 μL of PBS buffer added to 150 μL of erythrocyte suspension was taken as negative hemolysis control and 50 μL of Triton X-100 (1% v/v) was used as positive hemolysis control. The plate was incubated for 1 h at 37 □ and was then centrifuged at 3500 rpm for 5 min. 100 μL of the supernatant was then transferred to a new 96-well plate and absorbance was measured at 540 nm using a Tecan InfinitePro series M200 Microplate Reader. Percentage of hemolysis was determined as $(A – A_0)/ (A_{total} – A_0) \times 100$, where A is the absorbance of the test well, A_0 is the absorbance of the negative controls, and A_{total} the absorbance of wells containing Triton X-100. HC_{50} (concentration which causes 50% hemolysis relative to the positive control) was determined by plotting hemolysis as a function of compound concentration. The HC_{50} values are reported as averages of triplicates taken during one experiment.

2.2 Alamar blue assay[3]

In brief, HEK 293 cells that were maintained in complete DMEM media (Gibco) supplemented with 10% FBS (Gibco) and Penicillin-Streptomycin solution (Gibco) were seeded in 96-well plates at a concentration of 2×10^4 cells/well. They were allowed to adhere to the plate overnight. Media and 1% v/v Triton-X were used as untreated and positive controls respectively. The cells were treated with respective test compound/combination solutions. After 24 h of treatment, alamar blue reagent was added to the wells. The cells were incubated with alamar blue for 4 h. The absorbance was then measured at 570 nm, with 600 nm as the reference wavelength with a Tecan InfinitePro series M200 Micro plate Reader. Percentage of viable cells was determined as $A/A_0 \times 100$, A is the absorbance of the test well, A_0 is the absorbance of the wells without any treatment. Percentage cell viability was plotted as a function of concentration and EC_{50} determined where the cell viability was equal to 50% of the untreated control. The experiment was performed twice independently with three technical replicates each time.

2.3 Isolation of peripheral blood mononuclear cells and cytotoxicity[4]

Human peripheral blood mononuclear cells (hPBMCs) were isolated from freshly drawn human blood using a standard Ficoll-Hypaque density centrifugation technique, and the number of PBMCs, and their viability was determined by trypan blue exclusion. More than 95% of cells were viable. After isolation, human PBMCs were resuspended in RPMI 1640 growth medium (with L-glutamine and sodium bicarbonate from Gibco, Life Technologies) supplemented with 10% of fetal bovine serum (FBS, Gibco, Life Technologies) and 1%

Antibiotic-Antimycotic solution (consisting of penicillin, streptomycin and amphotericin B). 1 × 10^5 cells/well were seeded into 96-well plates and incubated overnight at 37 °C in a humidified-air atmosphere (5% CO_2 and 95% humidity). After 24 hours, alamar blue reagent was added to the wells, incubated for 18h. The absorbance was then measured at 570 nm, with 600 nm as the reference wavelength with a Tecan InfinitePro series M200 Micro plate Reader. Percentage of viable cells was determined as $A/A_0 \times 100$, A is the absorbance of the test well, A_0 is the absorbance of the wells without any treatment. Percentage cell viability was plotted as a function of concentration and EC_{50} determined where the cell viability was equal to 50% of the untreated control. The experiment was performed twice independently with three technical replicates each time.

3. Fluorescence microscopy of Madin-Darby Canine Kidney (MDCK) cells[3]

~2 × 10^4 MDCK cells were seeded into each well of a 96-well plate overnight under 5% CO_2 atmosphere at 37 °C. 120 µL of 1024 µg/mL solution of LBDiphe in DMEM medium (supplemented with 10% FBS and 1% antibacterial-antimycotic agent) was added to the seeded cells and incubated for 24 h. One row of cells was treated with 1% v/v Triton-X and one row was left untreated as positive and negative controls, respectively. Post-incubation, cells were washed with 1× PBS and then stained with 50 µL of 1:1 calcein-AM (2 µM) and propidium iodide (PI) (4.5 µM) for 15 min under 5% CO_2 atmosphere at 37 °C. The excess dye was then removed by washing the cells with 1×PBS, and images were captured with the 40× objective of a Leica DM2500 fluorescence microscope. During imaging, a band-pass filter for Calcein AM (at 500−550 nm) and a long-pass filter for PI (at 590−800 nm) was used. The experiment was performed twice independently.

4. Potentiation efficacy of adjuvants-Chequer board assays[5]

A solution of 25 µL each of antibiotic and adjuvants of concentrations varying 2-fold was added into each well of a 96-well plate followed by 150 µL of bacterial suspension (~5.0 × 10^5 CFU/mL) and incubated at 37 □ for 18-24 h. Bacterial suspension alone and Mueller Hinton broth alone served as controls. Each fractional inhibitory concentration (FIC) obtained was a result of two independent experiments. Different bacterial strains obtained from ATCC and MTCC as well as clinical isolates obtained from hospitals were used for this assay. The experiment was performed twice independently.

5. Time-kill kinetics of combinations[6]

The bactericidal activity of the combinations was assessed by the kinetics or the rate at which it affects the killing action of the combinations. Briefly, bacteria were grown in nutrient broth at 37 ℃ for 6 h. Test combinations at different concentrations of the antibiotic and adjuvant or individual treatments of the antibiotic and adjuvant were inoculated with the aliquots of bacteria resuspended in fresh media at $\sim 10^5$ CFU/mL. After specified time intervals (0, 1, 2, 4, 6, and 12 h), 20 µL aliquots were serially diluted 10-fold in 0.9% saline, plated on sterile MacConkey agar plates and incubated at 37 ℃ overnight. The viable colonies were counted the next day and represented as $\log_{10}$ (CFU/mL). The experiment was performed in duplicate.

6. Stability in physiological media[7]

To examine the susceptibility of LBDiphe to serum proteases, the antibacterial activities of L-LBDiphe and D-LBDiphe was tested in the presence of 50% of human plasma and mouse liver homogenate. Briefly, 250 µL of 16X solutions (1024 µg/mL) of the compounds were incubated with 250 µL of fresh human plasma and mouse liver homogenate at 37 °C for 12 h. Post-incubation, 25 µL of these samples was diluted serially row-wise by 2-fold in 1X PBS and added to 25 µL of serially diluted solutions of antibiotic (fusidic acid/minocycline). Chequerboard assays were conducted to determine the FICs of the two components as described previously. To understand whether the reduction in potentiation ability was due to nonspecific interaction with the proteins present in physiological fluids, high resolution mass spectrometry studies were conducted. Briefly, 100 µg/mL of D-LBDiphe and LBDiphe was incubated with 50% liver homogenate for 24 h. Cold methanol was added to the suspension 4 times the volume of the initial suspension in order to precipitate down the proteins. The solution was kept upright at -20 °C for 1 h. The solution was then centrifuged at 3500 rpm for 10 minutes. The supernatant was carefully collected, passed through a 0.35 µm syringe filter and assessed in a high-resolution mass spectrometer. The total ion chromatogram (TIC) was analysed for the presence of any m/z peaks corresponding to the compound or any cleaved components from the compound. The experiment was performed twice independently.

7. Mechanism of potentiation against planktonic bacteria [6]

7.1 Outer membrane permeabilization assay

Mid-log phase *P. aeruginosa* R590 cells (~10^8 CFU/mL) were harvested, washed with 5 mM HEPES and 5 mM glucose and resuspended in a 1:1 solution of the same. To this suspension, *N*-phenylnaphthylamine dye was added to give a final concentration of 10 μM. 190 μL of the suspension containing the dye was then added to the wells of a 96-well plate (black plate, clear bottom with lid) and stabilized for 6 min. Then, 10 μL of the adjuvant was added to the solution at 10-times the desired concentrations (8-64 μg/mL). After addition, fluorescence intensity ($\lambda_{excitation}$ = 350 nm; $\lambda_{emission}$ = 420 nm) was measured every two minutes for 30 more minutes. The experiment was performed twice independently with two technical replicates each time.

7.2 Cytoplasmic membrane depolarization assay

Mid-log phase bacterial cells (~10^8 CFU/mL) were harvested, washed with 5 mM HEPES and 5 mM glucose were resuspended in 1:1:1 solution of 5 mM glucose, 5 mM HEPES and 100 mM KCl solution, supplemented with 200 μM of Na_2EDTA. $DiSC_3(5)$ was added to this suspension to achieve a final concentration of 2 μM. After preincubation for 45 minutes in the wells of a black 96-well plate with a transparent bottom for dye uptake, and resultant self-quenching, the fluorescence of the bacterial suspension was measured ($\lambda_{excitation}$ = 622 nm; $\lambda_{emission}$ = 670 nm) and allowed to stabilize for 8 minutes at room temperature. Then, 10 μL of adjuvant was added to the wells at the same concentrations as mentioned in the previous assay. After addition, fluorescence intensity was measured every two minutes for 30 more minutes. The experiment was performed twice independently with two technical replicates each time.

7.3 Assessment of membrane potential using flow cytometry[8]

Mid-log phase bacterial cell (~10^8 CFU/mL) were co-incubated with 10 μM of $DiOC_2(3)$ dye and varying concentrations of D-LBDiphe (16, 32, 64 μg/mL)/ CCCP (5 μM) for 30-45 minutes at 37 °C in dark. After incubation, the samples were taken for analysis in a flow cytometer (BD Biosciences) by using an excitation wavelength of 488 nm and appropriate emission filters for both red (propidium iodide) and green (FITC) channels. Gating and analysis were done using FCS Express 6 software. The experiment was performed twice independently.

7.4 Inhibition of efflux machinery using flow cytometry[9]

Freshly grown mid-log-phase culture of *P. aeruginosa* R590 bacterial cells was centrifuged (9000 rpm for 5 min) and resuspended in MHB. Bacterial cells (~10^8 CFU/mL) were incubated with 16 μg/mL EtBr for 1 h at 37 °C and 150 rpm. The cells were centrifuged (9000 rpm for 5 min), washed to remove excess EtBr, resuspended in fresh MHB, divided it into different

aliquots and treated with different concentrations of D-LBDiphe (8, 16, 32 μg/mL) and CCCP (20 μg/mL). After 2 hours of incubation at 37 °C and constant shaking at 150 rpm, the solutions were taken for analysis in a flow cytometer (BD Biosciences) by using an excitation wavelength of 530 nm and appropriate emission filter for R-phycoerythrin Gating and analysis were done using FCS Express 6 software. The experiment was performed twice independently.

7.5 Microscopy using FM 4-64 dye[10]

Mid-log phase grown GFP-tagged *P. aeruginosa* PAO1 were treated with 16 μg/mL of D-LBDiphe and 2 μg/mL of CTAB in 1X-PBS for 2 h at 37 °C. After 2 h, 200 μL of 1X-PBS containing 5 μg/mL of FM 4-64 was added to the bacterial pellets, obtained after centrifugation at 5000 rcf. The bacterial cells were allowed to incubate with the dye for 10 minutes and then the cells were immediately imaged in a confocal microscope. Immediate imaging is important to prevent signal due to endocytosis of the dye. The experiment was performed twice independently.

7.6 Preparation of Liposome and Calcein Dye Leakage Assay[11]

1-palmitoyl-2-oleoyl-sn-3-glycero-phosphoethanolamine (POPE) and 1-palmitoyl-2-oleoyl-sn-3-glycero-phosphatidylglycerol (POPG) were weighed and dissolved in chloroform to obtain stock solution of 25 mg/ml each. Model bacterial membrane containing 7:3 POPE/POPG LUVs are considered as a model system for Gram-negative bacterial outer membrane inner leaflet or inner membrane.[12-13] This model liposome was dried in a nitrogen stream and lyophilized overnight to enable the formation of lipid film. The lipid film was re-suspended in 10 mM Tris buffer (pH 7.4), containing 70 mM calcein, followed by vigorous vortexing for 20 mins. The film was then subjected to five freeze-thaw cycles in liquid nitrogen to obtain vesicles entrapped with calcein. Unilamellar vesicles were obtained by passing the suspension further through a mini extruder (Avanti Polar Lipids, Alabaster, AL) using stacked 100 nm pore size polycarbonate membrane filters. The samples were passed through the filter for 23 times. Untrapped calcein was removed by passing the formed vesicles through a gel filtration based hydrated Centrisep- Spin Column, followed by centrifugation at 3000 rpm for 2 mins.

10μl of dye-encapsulated liposome representing model bacterial membrane mimic was taken in extra vesicular buffer containing 10 mM Tris, 100 mM NaCl (pH 7.4). Calcein leakage was monitored by the fluorescence emission at 519 nm by liposome disruption using Hitachi

F-7000 FL spectrophotometer (slit width: 2.5 nm). D-LBDiphe molecule was added at various concentrations and enhancement in fluorescence was measured. 0.1 % Triton X- 100 served as a control, completely disrupting the calcein- encapsulated unilamellar vesicles.

Percentage leakage was calculated using the equation:

% dye leakage= $[(F-F_o)/ [(F_T-F_o)] \times 100$ %,

Where, F_o, F, F_T denotes the basal fluorescence intensity, florescence intensity after addition of peptides and maximum florescence intensity obtained after addition of 0.1% Triton X 100, respectively.

7.7 Molecular dynamics simulation studies

Model membrane systems were generated using the CHARMM-GUI web server and CHARMM36 force field was used to describe the potential. The composition of membranes was kept same in top and bottom layer.[14] Force field parameters for D-LBDiphe and CTAB were generated using CGenFF program.[15] The composition of lipids used for gram-negative bacterial membrane was 252 POPE + 84 POPG + 13287 TIP3P water molecules and for mammalian membrane was 238 POPC + 102 CHL + 13633 TIP3P water molecules. Further, Na^+ and Cl^- ions were added to neutralize the charge and to keep the ionic concentration to 0.15M of NaCl. The temperature of the system was maintained at 300 K by employing Nose-Hoover thermostat[16] with a friction coefficient of 1.0 ps. The pressure of the system was maintained at 1 bar employing Parrinello-Rahman barostat.[17] Hydrogen containing bonds were constrained using LINCS constraints[18] which enabled us to use 2 fs time step for integrating equations of motion. Initially, pure model membrane-water system was equilibrated until their mechanical properties (like area per lipid, thickness and mean square displacement) were converged (data not provided). The simulations were performed in NPT ensemble. Once equilibrated, ten molecules of D-LBDiphe/CTAB molecules were added to the Gram-negative and Mammalian membrane Molecular dynamics (MD) simulations were performed using GROMACS-2019 simulation package.[19] A 12 A° cut-off was employed for van der Waals and electrostatic interactions, and neighbour list was updated every 20 MD steps. Particle-Mesh Ewald method was used to compute electrostatics.[20] VMD software was for visualizing interactions of D-LBDiphe and CTAB with the model membranes.[21]

To compute the free energy of insertion of CTAB into the mammalian membrane, two independent well-tempered metadynamics (WTMTD) simulations[22] were performed with

different initial velocities. The *z*-component of distance vector of center of mass (COM) of all the C_{18} atoms (terminal atoms) of phospholipids to the COM of CTAB molecules (only heavy atoms) was taken as collective variable for WTMTD. Only five CTAB molecules were taken for the WTMTD for the convenience of restraining the CTAB molecules to one side of the leaflet. The restraining potential used has the form:

$$V_{\mathrm{wall}}(s) = \sum_{i}^{5} k_i \left(\frac{s_i - s_0}{\epsilon_i}\right)^2$$

Here $k_i \sim 400$ kJ nm^{-2}, $s_0 = -4.5$ nm and $\varepsilon_i = 1.0$ was used. The wall potential act on the CTAB molecules only when $s < s_0$. Gaussian bias functions used in WTMTD had an initial height of 2.4 kJ mol^{-1}, and width of 0.05 nm. Bias update frequency of 500 MD steps, and the bias factor 5 was used in WTMTD. The free energy profile was constructed by summing all the bias added during the simulation. GROMACS-2019 patched with PLUMED-2.6.1[23] was used to perform these simulations.

8. Culture of stationary phase bacteria[24]

Bacterial stocks of MRSA and *P. aeruginosa* taken from -80℃ were streaked onto agar plates. A single colony of each bacterium was inoculated in nutrient broth medium and grown to mid-log phase for 6h at 37 °C and 150 rpm shaking. This mid-log phase culture was diluted 1000-fold in nutrient broth media, allowed to culture for 16 h at 37 °C and 150 rpm shaking to reach stationary phase.

9. Culture of *S. aureus* persisters[24]

S. aureus MTCC 737 persisters were generated by first generating *S. aureus* stationary phase bacteria. The stationary phase culture was diluted by 10-fold in fresh nutrient broth media or mueller hinton broth media and treated with 100 µg/mL of ampicillin for 3 h at 37 °C with vigorous shaking. Ampicillin was then removed by centrifugation, washing and resuspending the cells in 1X PBS.

10. Bactericidal kinetics against growth-restricted phases of bacteria

The stationary phase cells of MRSA and *P. aeruginosa* and persister cells of *S. aureus* were grown and resuspended in 1X-PBS. 150 µL of *P. aeruginosa* stationary cells were treated with different concentrations of D-LBDiphe (64 µg/mL), fusidic acid (32 µg/mL), combination of D-LBDiphe (32 µg/mL) and fusidic acid (8 µg/mL) and combination of fusidic acid (16 µg/mL) and D-LBDiphe (64 µg/mL). Two independent experiments were performed against MRSA

stationary phase cells. In one case, they were treated with different concentrations of D-LBDiphe (16 μg/mL), fusidic acid (16 μg/mL), combination of D-LBDiphe (16 μg/mL) and fusidic acid (8 μg/mL). *S. aureus* persisters were treated with fusidic acid (16 μg/mL), D-LBDiphe (16 μg/mL), ampicillin (50 μg/mL), combination of fusidic acid (16 μg/mL) and D-LBDiphe (16 μg/mL). At various time points of 2 h, 4 h, 6 h, 12 h and 24 h post incubation with compounds, 20 μL aliquots of the suspension was serially diluted by10-fold and 20 μL of each dilution was drop-plated on nutrient agar (for MRSA and *S. aureus*) and MacConkey agar (for *P. aeruginosa*). The viable bacteria were then counted after 18-24 h incubation at 37 °C at static conditions. In each case, two biological replicates were performed with two technical replicates each time.

11. Membrane-perturbing properties against stationary phase bacteria

11.1 Membrane permeabilization against stationary phase bacteria

Membrane permeabilization against stationary phase MRSA and *P. aeruginosa* R590 was performed with modifications. Stationary phase cultures of MRSA and *P. aeruginosa* were harvested (3500 rpm, 5 min), washed and resuspended in 5 mM HEPES buffer (pH = 7.2). D-LBDiphe and CTAB at 8 μg/mL and 16 μg/mL were added to the bacteria in black-well, clear bottom 96-well plates. Fluorescence was monitored for 30 minutes. Two independent experiments were performed with two technical replicates each time.

11.2 Membrane depolarization against stationary phase bacteria

Briefly, stationary phase cultures of MRSA and *P. aeruginosa* R590 were washed and harvested in 5 mM HEPES buffer (pH 7.2) and resuspended in 1:1 solution of 5 mM HEPES buffer and 100 mM KCl solution, supplemented with 200 μM (for only Gram-negative bacteria). 2 μM of 3,3′-Dipropylthiadicarbocyanine iodide (DiSC3(5)) was then added, the suspension put into black-well 96-well plate (190 μL/well) and incubated in dark at room temperature for 45 minutes. 10 μL of 10 times the test compound concentration (D-LBDiphe and CTAB) was added to black well plates containing bacterial suspension and $DiSC_3(5)$ after 8 min of fluorescence measurement. The fluorescence was monitored for about 30 min, at 2 min intervals to measure membrane depolarization. Two independent experiments were performed with two technical replicates each time.

11.3 Outer membrane permeabilization against stationary phase bacteria

Stationary phase cultures of *P. aeruginosa* R590 cells were resuspended in 5 mM HEPES buffer and then further diluted in HEPES buffer by 4-times. *N*-phenylnaphthylamine dye (NPN) was added to a final concentration of 5 μM to the bacterial suspension. 190 μL of this suspension was put into flat clear bottomed black 96-well plates. After measuring the fluorescence intensity for 8 minutes, 10 μL solution of 10- times required concentration of test compounds (D-LBDiphe, CTAB) was added to the wells. After addition of compounds, the fluorescence intensity of NPN was monitored for 30 about minutes. Two independent experiments were performed with two technical replicates each time.

12. Growth of mature biofilms[24-26]

Previously reported protocols were followed for the growth of mature biofilms of *A. baumannii*, MRSA, *P. aeruginosa*. Mid-log phase grown MRSA was diluted to 10^5 CFU/mL in nutrient broth supplemented with 1% w/v glucose and 1% w/v NaCl (biofilm media). Sterile glass cover slips of 18 mm diameter were placed in 6-well plates. 2 mL of MRSA was incubated with the glass cover slips at 37 °C for 24 h in order to allow formation of mature biofilms. After 24 h, the cover slips were removed and carefully washed with 0.9% saline to remove planktonic bacteria. Biofilms were then treated with compound solutions prepared in biofilm media for 24 h or twice with 24 h interval between dosing. For *A. baumannii*, mid-log phase culture of *A. baumannii* was diluted to a concentration of approximately 10^5 CFU/mL in chemically defined BM2 medium containing 0.5% glucose as carbon source, 0.5% casamino acids and 200 μM $FeCl_3$, to make the bacterial solution. Sterile glass cover slips were incubated with this bacterial solution in 6-well plates at 30 °C for two days (48 h). After 48 h, the cover slips were washed carefully and treated with compound solutions in BM2 media for 24 h. The second dose was given by washing the biofilms after 24 h of treatment and giving the second dose in BM2 media for another 24 h. 2 mL of mid-log phase culture of *P. aeruginosa*, diluted to approximately 10^5 CFU/mL in nutrient broth supplemented with 1% glucose (w/v) and 1% NaCl (w/v) was added to wells of a 6-well plate containing glass cover slips. The biofilm was allowed to form under stationary conditions at 33-37 °C for 72 h. After 72 h, the cover slips were washed in 0.9% saline and two doses of treatment were given over 48 h at an interval of 24 h. To grow mixed species biofilms of MRSA and *P. aeruginosa* R590, 10^5 CFU/mL of MRSA and *P. aeruginosa*, diluted into nutrient broth supplemented with glucose and NaCl, was added to 6-welll plates

containing glass cover slips (18 mm diameter). The biofilm was allowed to mature under stationary conditions at 37 °C in humidified atmosphere for 48 h. After 48 h, the cover slips were washed and carefully placed into nutrient broth (biofilm media) containing different treatments in 6-well plates. One dose of treatment was given for 24 h, after which the cover slips were washed and placed into nutrient broth containing the second dose of treatment. 24 h post second dose, the cover slips were either taken for bacterial count enumeration or confocal imaging. For mixed biofilms of GFP-tagged SH1000 and dsred-tagged PAO1, 10^5 CFU/mL of PAO1 and 10^6 CFU/mL of SH1000 diluted into nutrient broth with 1% NaCl and 1% glucose was incubated with glass cover slips in 6-well plates for 48 h at 37 °C. After 48 h, the cover slips were washed, and two doses of treatments were given at intervals of 24 h.

13. Biofilm disruption assays[24]

13.1 Viability of biofilm-embedded cells and dispersed cell count

Mature biofilms of *A. baumannii* were generated and treated with D-LBDiphe (64 µg/mL), minocycline (4 µg/mL), fusidic acid (16 µg/mL), combination of fusidic acid (16 µg/mL) and D-LBDiphe (64 µg/mL), combination of minocycline (4 µg/mL) and D-LBDiphe (32 µg/mL) and colistin (16 µg/mL) for 24 h or a second dose at the same concentrations was given after 24 h, which was again incubated with the biofilm for 24 more hours. The biofilms on glass cover slips were carefully removed from the well and washed twice in 0.9 % saline post-treatment with the test compounds as described in the previous section. They were then treated with 1:10 ratio of 0.1 % Trypsin- EDTA solution in 0.9% saline for 15 minutes at 37 °C with 100 rpm shaking to digest the biofilms. The cover slips were scratched to ensure that biofilms are completely digested. Aliquots of the digested biofilms were then serially diluted 10-fold and different dilutions were drop-plated on MacConkey agar plates. The number of viable colonies were counted after 18-24 h. Further, the dispersed suspension from the biofilm after different treatments were also serially diluted by 10-fold and different dilutions were drop-plated on MacConkey agar plates. This was used to enumerate the dispersed cell count after various treatments. In each of the following cases, two independent experiments were performed with two technical replicates each time.

For *A. baumannii* biofilms, as two doses of treatments was better in reduction of biofilm-embedded cell viability, two doses of treatments were given in *P. aeruginosa*, MRSA and mixed biofilms of *P. aeruginosa* and MRSA. All the biofilms were generated as described in section 5.4.5. The treatments given for *P. aeruginosa* biofilms were fusidic acid (16 µg/mL),

D-LBDiphe (16 µg/mL), combination of fusidic acid (16 µg/mL) and D-LBDiphe (16 µg/mL) and colistin (16 µg/mL). After two doses of treatments for 48 h, the biofilms were digested as described previously, serially diluted, drop-plated on MacConkey agar plates and counted after 18-24 h. The dispersed cells after two doses of treatment were also counted for the same.

For MRSA biofilms, the same protocol was followed, and the treatments given were fusidic acid (16 µg/mL), D-LBDiphe (16 µg/mL), combination of fusidic acid (16 µg/mL) and D-LBDiphe (16 µg/mL).

For mixed biofilms of PAR590 and MRSA ATCC 33591, fusidic acid (16 µg/mL), D-LBDiphe (32 µg/mL), combination of fusidic acid (16 µg/mL) and D-LBDiphe (32 µg/mL) and colistin (16 µg/mL). For mixed biofilms of GFP and dsred tagged bacteria, the treatments given were D-LBDiphe (64 µg/mL), fusidic acid (16 µg/mL), minocycline (8 µg/mL), combination of fusidic acid (16 µg/mL) and D-LBDiphe (64 µg/mL), combination of minocycline (8 µg/mL) and D-LBDiphe (64 µg/mL) and colistin (16 µg/mL).

13.2 Confocal laser scanning microscopy

Mature biofilms were generated and treated as mentioned in the previous sections. Glass cover slips post-treatment with the test compounds at the mentioned concentrations, and the untreated control were carefully removed from the well, washed with 0.9 % saline, and placed on glass slides. The biofilms were then stained with 7 µL of SYTO9 (60 µM) and Propidium iodide (15 µM) and imaged using a Zeiss 510 Meta confocal laser-scanning microscope. The 3D-stacked images were processed ImageJ. No staining solution was used for GFP and dsred-tagged bacterial biofilms and they were directly imaged in a confocal microscope. The experiment was performed in duplicate.

14. Anti-virulence properties

14.1 Pyocyanin assay[27]

Previously reported protocol with slight modifications was followed. The amount of pyocyanin, which is an important virulence factor in *P. aeruginosa* was investigated in PAO1. Briefly, overnight cultures of PAO1 were diluted to an O.D.$_{600}$ ~ 0.05 and inoculated in nutrient broth at 37 °C with shaking at 150 rpm. The bacterial culture was allowed to grow for 6 h up to mid-exponential phase (O.D.$_{600}$ = 0.4). The bacteria suspension was diluted if the O.D. was higher than 0.4. D-LBDiphe at a concentration of 8 µg/mL was incubated with the bacteria in a tube at 37 °C with 120 rpm shaking. The untreated control was also incubated. Three technical

replicates were performed. The supernatants from each tube were recovered in the late stationary phase (24 h) by centrifuging the tubes at 4500g for 20 minutes at 4 °C. The supernatants were filtered using a 0.35 µm syringe filter and the cell-free supernatants were kept on ice until further required. Pyocyanin was extracted in the organic layer by mixing the supernatant with an equal volume of $CHCl_3$. The tubes were mixed vigorously and incubated at 37 °C for 25 minutes. The extraction was demarcated by a colour change in the organic layer from colourless to slightly greenish blue. The aqueous layer was gently removed without disturbing the organic layer. The pigment was reextracted by mixing the $CHCl_3$ layer with a slightly higher volume of 0.2 M HCl. The aqueous layer turned pinkish red after extraction. 200 µL of this solution from every replicate was put in the wells of a 96-well plate and absorbance was measured at 520 nm, with 0.2 M HCl as blank. The percentage of pyocyanin production relative to the untreated PAO1 control samples was calculated. Experiment was performed in duplicate.

14.2 Staphyloxanthin assay[28]

Previously reported protocol with slight modifications was followed. The amount of staphyloxanthin was determined in MRSA. Briefly, mid-log phase MRSA ($O.D._{600}$ ~ 0.1) was incubated with D-LBDiphe at 8 µg/mL and 16 µg/mL in well-aerated tubes at 37 °C with shaking at 200 rpm for 24 h. Three technical replicates of each treated concentration and the control were used. Cells were harvested and washed with 1X-PBS. The cells were then treated with 1 mL methanol (100% v/v) for 20 minutes at 40 °C in a water bath. The amount of staphyloxanthin in the cooled methanolic extract was measured by taking absorbance of 200 µL of the sample in 96-well plate at 463 nm. The O.D. was normalized to the cell O.D. at 600 nm. The experiment was performed in triplicate.

15. Intracellular antibacterial activity[29-30]

1×10^5 RAW 264.7 cells were seeded in 24-well tissue culture plates containing complete growth medium at 37 °C in humidified air containing 5% CO_2 for 14 h to allow cell adherence. 500 µL of PAO1 at a multiplicity of infection (MOI) of 4 in DMEM supplemented with 10% FBS medium were added to the wells and incubated at 37 °C in humidified air containing 5% CO_2 for 1 h to allow phagocytosis of the cells, Next, the macrophages were washed with 1X-PBS (pH = 7.2) and treated with 50 µg/mL of gentamicin in DMEM supplemented with 10% FBS for 1 h to remove any extracellular non-phagocytosed bacteria. The cells were washed twice with 1X-PBS to remove gentamicin. Cells in two wells were lysed using 500 µL of ice-

cold water for 30 minutes, lysates were collected, centrifuged and resuspended in 1X-PBS. Lysates were serially diluted 10-fold and drop-plated on MacConkey agar plates. This gave the pre-treatment intracellular bacterial load. The rest of the wells were treated with D-LBDiphe (128 μg/mL), minocycline (128 μg/mL), minocycline (64 μg/mL) + D-LBDiphe (64 μg/mL), minocycline (64 μg/mL) +D-LBDiphe (128 μg/mL), meropenem (50 μg/mL); and incubated at 37 °C in humidified air containing 5% CO_2 for 24 h. The treatments were given in DMEM media supplemented with 10% FBS. After 24 h, the cells were then lysed by treating with 500 μL of ice-cold water for 30 minutes and the lysates were then collected, centrifuged and resuspended in 1X PBS. Lysates were serially diluted 10-fold and plated on MacConkey agar plates as described previously. The plates were then incubated at 37 °C for 24 h and the viable bacterial colonies were counted. The results represented are the average of two independent experiments carried out in duplicates each time.

16. Study of interaction with endotoxin and immunomodulatory properties

16.1 Antagonization assay with LPS and $MgCl_2$[31]

The potentiation activity of D-LBDiphe was tested with minocycline against *E. coli* R250 and *E. coli* R4806. The potentiation activity of D-LBDiphe was also tested with fusidic acid against *P. aeruginosa* R590. Briefly, various two-fold diluted concentrations of minocycline were incubated with 50 μg/mL of LPS and 2-8 μg/mL of D-LBDiphe together depending on the strain of *E. coli*. After incubation for 1 hour, ~125 μL of ~10^5 CFU/mL bacteria were added to the 96-well plates, incubated at 37 °C for 18-24 h and $O.D._{600}$ measured to determine the FIC values. Minocycline, at different concentrations was also incubated with D-LBDiphe and 20 mM $MgCl_2$, bacterial suspension added immediately and the $O.D._{600}$ measured after 18 h. Against, *P. aeruginosa* R590, varying concentrations of fusidic acid were either incubated with 0.5 mg/mL LPS and 32 μg/mL of D-LBDiphe together or 20 mM $MgCl_2$ and 32 μg/mL of D-LBDiphe together, bacterial suspension added and $O.D._{600}$ measured. The graphs were plotted in OriginPro (2022) software.

16.2 DLS studies with LPS[4]

E. coli 0111:B4 LPS and D-LBDiphe was dissolved in Hank's balanced salt solution (HBSS without Ca^{2+} and Mg^{2+}). Experiments were performed using Zetasizer Nano Z (Malvern Instruments) at room temperature. LPS and D-LBDiphe were taken in disposable cuvettes in 2 mL of solution. 25 μg/mL of LPS was treated with 25 μg/mL or 50 μg/mL of D-LBDiphe and

its scattering measured in the instrument. D-LBDiphe solution in HBSS and only buffer was taken as controls. Data for a single treatment was acquired for three experiments. Two independent experiments were performed.

16.3 Fluorescence studies with LPS[4]

BODIPY-conjugated LPS (from *E. coli*, Molecular Probes, Life Technologies) was used to study the interaction of LPS with D-LBDiphe. Stock solution of BODIPY-LPS (100 µg/mL) was prepared in 1X-PBS (pH 7.4). BODIPY-LPS stock solutions were sonicated for 2 min before use. From the stock solution, 2 mL solution containing BODIPY-LPS (500 ng/mL) in 1X-PBS was prepared in a quartz cuvette. D-LBDiphe (16, 32, 64 µg/mL) and SDS solution (2% w/v) treatments were given to the solution and fluorescence intensity checked using a λS55 fluorescence spectrophotometer (PerkinElmer) at an excitation wavelength of 485 nm, and emission was collected from 500 to 700 nm (excitation slit width = 10 nm, emission slit width = 15 nm, speed = 400 nm/min) at room temperature.

16.4 Isothermal calorimetry studies with LPS[32]

The binding interaction of D-LBDiphe with LPS was assessed using TA-affinity ITC (TA Instruments, New Castle, USA). Stocks of D-LBDiphe and LPS were prepared in 10 mM phosphate buffer (pH 7.4) followed by degassing of the buffer, peptide stocks as well as D8PG stock. A 182 µl sample cell containing D-LBDiphe at concentration of 1mg/ml was titrated with LPS from a stock solution of 10 mg/ml at 310K and stirring speed of 100 rpm. A total of 40 injections, at an interval of 180 s with 2 µl of LPS aliquots per injection were performed. Nano Analyze 3.7.5 software was used to plot the raw data. Each plot was fitted using a nonlinear equation, and an independent binding site model was utilized to analyze number of binding sites (n) and the thermodynamic parameters, which include dissociation constant (K_d), change in enthalpy (ΔH), free energy of binding (ΔG) and entropy (ΔS). Dissociation constant corresponds to the concentration of ligands (in Molar range) bound to half the binding sites of LPS at equilibrium.

$$K_D = \frac{[L][P]}{[LP]}$$

$\Delta G = -RT \ln K_a$ and $\Delta G = \Delta H - T\Delta S$.

16.5 Saturation Transfer Difference NMR studies with LPS[33-34]

D-LBDiphe was dissolved in 550 μl of D_2O and pH was adjusted to 4.5 using a small amount of 0.1 M HCl or 0.1 M NaOH. After adjusting the pH of the solution, the volume was adjusted to 600 μl using D_2O with a final concentration of 1 mg/ml. The stock solution of *E. coli* 0111: B4 LPS (concentration 10 mg/ml) was also prepared in D_2O solution, and the pH was adjusted to 4.5. Both the D-LBDiphe and LPS stock solution was subjected to double lyophilization to remove any trace amount of water. The lyophilized samples were dissolved in 100% D_2O before the NMR data acquisition. All NMR spectra were recorded on a Bruker Avance III 500 MHz spectrometer, equipped with 5 mm RT probe at 298 K. Data acquisition and processing were performed with Topspin 3.1 software (Bruker).

STD Experiments: STD NMR spectra were acquired at an D-LBDiphe/LPS mixture ratio of 100:1. Selective irradiation of LPS was achieved by a series of Gaussian-shaped pulses with a 1% truncation and each of 49 ms in duration and separated by a 1 ms delay. A total of 40 selected pulses were applied, leading to a total time of saturation of 2 s. The so-called on resonance for LPS was fixed at -1.0 ppm, and off-resonance was at 40 ppm, where neither LPS nor the D-LBDiphe resonances were present. Subtraction of the two spectra (on-resonance−off-resonance) by phase cycling leads to the difference spectrum that contains signals arising from the saturation transfer. The reference spectrum was recorded with 2048 scans, while the difference spectrum was obtained with 4096 scans. In order to identify the group epitope mapping, seven saturation times (T_{sat}): 0.5, 1.0, 1.5, 2.0, 2.5, 3.0, and 5.0 s were chosen to generate the STD build-up curves using the monoexponential equation (Figure S12):

$$STD_{amplification\,factor} = STD_{max}(1 - e^{-k_{st}.t})$$

where STD_{max} is the maximal STD intensity and k_{st} is the saturation rate constant for D-LBDiphe (0.5 mM) in the presence of *E. coli* 0111: B4 LPS at zero saturation time.

16.6 Immunomodulatory studies[4]

Briefly, human PBMCs were isolated from blood. Freshly isolated human PBMCs were seeded into 24-well plates (1×10^6 cells/well) in 1 mL of RPMI 1640 complete medium. After 3 h of resting, the cells were stimulated with 20 ng/mL of *E. coli* 0111:B4 LPS (Sigma-Aldrich) or 100 ng/mL of LTA from *S. aureus* (Sigma Aldrich) in either the absence or presence of D-LBDiphe (16 μg/mL and 32 μg/mL). A control experiment was performed using 1X-PBS as vehicle control. The cells were incubated for 18–24 h and then cell culture supernatants were analysed for cytokines such as tumour necrosis factor (TNF-α), interleukin- 6 (IL-6) and

interleukin-1β (IL-1β) using the human ELISA kits (ABclonal Technologies) following the manufacturer's instructions. Three replicates were performed for the experiment.

17. *In-vivo* systemic toxicity[6]

Systemic toxicity was examined in mice model by following OECD guidelines. BALB/c female mice of 8-10 weeks age with body weight of 22-24 g were used for the study and five mice were assigned in each group. Individual groups of mice were injected with D-LBDiphe solution (0.2 mL) in normal saline with recommended dosages of 5.6 mg/kg, 17.5 mg/kg, 56 mg/kg and 175 mg/kg through subcutaneous and intraperitoneal routes of administration. 0.2 mL of normal saline was injected as a control for each concentration of compound and each route of administration. Next, the survival of the mice on a per group basis were monitored for 14 days. Finally, based on the survival, 50% lethal dosage (LD_{50}) was determined for each administration route using the Spearman-Karber method.

18. *In-vivo* sub-chronic toxicity[6]

Sub-chronic toxicity was examined in mice model by following OECD guidelines. A combination dose of 30 mg/kg of minocycline and 30 mg/kg of D-LBDiphe were administered to 10 mice (5 mice per group) through intraperitoneal injection. 10 more mice were kept as the untreated control groups (5 mice per group). A group of mice treated with combination dose and one of the control groups were sacrificed through cervical dislocation at 48 h post-treatment. Retro-orbital blood collection was done from the sacrificed mice. The two remaining groups of treated and control mice were sacrificed 21 days post treatment and blood collection was done retro-orbitally. Finally, all the blood samples of individual mice were analyzed at RV Metropolis Clinical Laboratory (Bangalore, Karnataka, India) to measure the levels of desired blood markers such as alkaline phosphatase (ALP) for liver function, blood urea nitrogen (BUN) and creatinine for kidney function as well as electrolyte balance (Na^+, Cl^-, K^+).

19. Dermal toxicity[35]

Dermal toxicity was evaluated by following OECD guidelines (OECD 425). Briefly, two groups of mice (N = 5) were used for this experiment. Firstly, the mice were anesthetized by using 150 μL of xylazine-ketamine cocktail through intraperitoneal injection. Fur was shaved off from the dorsal area of mice using a trimmer and blade without causing any skin injury. 40 μL of D-LBDiphe (200 mg/kg in saline) was applied to the shaved portion of one group of mice using a pipette. The other group of mice was treated with 40 μL saline without any compound.

The mice were monitored for 14 days. At the end of the experiment all the mice were sacrificed through cervical dislocation and the dorsal area of skin excised for histopathological analysis through hematoxylin and eosin staining at Rohana Veterinary Diagnostic laboratory (Bangalore, Karnataka, India).

20. *In-vivo* acute skin infection model[35]

Reported protocol was followed with slight modifications to create an acute skin infection model.[252] The mice were housed in individually ventilated cages (IVC) maintained with controlled environment. A total of 20 mice were used in the experiment with 4 mice in each group. A post-hoc power analysis using GPower 3.1 revealed a power of ~99%. The animals were anesthetized with intraperitoneal injection of 150 µL of xylazine-ketamine cocktail. The fur of the dorsal region of the mice was shaved aseptically. Shaving was performed in a manner to induce a reddening wound without any substantial bleeding. A bacterial infection was initiated on the wound by placing a 20 µL droplet with ~10^7 cells of *P. aeruginosa* MTCC 424. The droplet was allowed to dry for some time. Four hours post infection, one group of mice was treated with 40 µL of 40 mg/kg of fusidic acid solution in saline and another group treated with 40 µL solution of 40 mg/kg of D-LBDiphe solution in saline. The third group was treated with 40 µL of combination treatment of 40 mg/kg D-LBDiphe and 40 mg/kg fusidic acid. Another group of mice was treated with 40 µL of 40 mg/kg of colistin solution in saline as a control antibiotic. Caution was observed to avoid any drop from rolling down the sides. The control group was left untreated. The dosage was continued for three more days after the first dosage (one dose every 24 h). 24 h after the last dose, mice were sacrificed using isofluorane and the infected skin area was excised aseptically. The excised skin tissues were placed into 10 mL of sterile saline in falcon tubes which were pre-weighed. The tissues were homogenized for 5 minutes using a tissue homogenizer. The homogenates were serially diluted by 10 folds in a 96-well and different dilutions were drop-plated onto agar plates. The plates were incubated for 18-24 h at 37 °C. The bacterial titre was expressed in terms of log (CFU/g) of weight of the tissue collected. The bacterial titre was plotted in GraphPad Prism software and one-way ANOVA was performed to calculate the significance of results.

21. Organ-level toxicity through haematoxylin and eosin staining

A combination dose of 30 mg/kg of minocycline and 30 mg/kg of D-LBDiphe were administered to BALB/c female mice through intraperitoneal injection. Mice were also kept for the untreated control group. The mice were euthanized through cervical dislocation, 2-days

post-treatment. Kidney and liver were isolated from both treated and untreated mice, stored in 10% neutral buffered formalin (NBF) and histopathological changes were analysed through haematoxylin and eosin staining. H & E staining and histopathological analysis were performed at Rohana Veterinary Diagnostic laboratory (Bangalore, Karnataka, India).

22. *In-vivo* immunomodulatory potential[36]

Male BALB/c mice (6–8 weeks old) were housed in individually ventilated cages (IVC) maintained with controlled environment. Total 16 mice were used for the experiment with four mice in each group. Group 1 of mice was administered with a single intraperitoneal (i.p.) dose of PBS; group 2 of mice was administered i.p. with 0.1 mg/kg of LPS; group 3 was administered i.p with a combination of LPS (0.1 mg/kg) and D-LBDiphe (30 mg/kg) (pre-incubated for 20 minutes before administration) and group 4 of mice was administered i.p with 30 mg/kg of D-LBDiphe. All groups of mice were sacrificed after 2 h. The blood was extracted through retroorbital sinus. Serum was isolated from whole blood and levels of IL-6 and TNF-α in sera were evaluated using mice ELISA kits (ABclonal Technologies) following the manufacturer’s instructions.

23. *In-vivo* toxicity via intratracheal route of administration

This experiment was outsourced to TheraIndx Lifesciences Pvt. Ltd. (Bangalore, India). Briefly, male BALB/c mice (6-7 weeks old) were divided into four groups (n = 5/ group). The groups received the following intratracheal treatments: saline, minocycline + D-LBDiphe (5.5 + 5.5 mg/kg), minocycline + D-LBDiphe (17.5 + 17.5 mg/kg), minocycline + D-LBDiphe (56 + 56 mg/kg). Mice were injected with above mentioned treatments (dose volume = 2 mL/kg), intratracheally in anesthetized animal using i.t. intubation using BIOLITE Intubation system (Brain scientific). Clinical signs (gait, posture, morbidity) were recorded before and immediately after dosing for up to 7 days. The clinical signs were graded based on degree of severity and reversibility of any signs.

24. *In-vivo* efficacy through intratracheal delivery route, against *P. aeruginosa* (PAO1) pulmonary infection

This experiment was outsourced to TheraIndx Lifesciences Pvt. Ltd. (Bangalore, India). Female BALB/c mice (6-8 weeks old) were divided into 7 groups (n = 5/group). Group1, pretreatment count or early infection control; Group 2, late infection/vehicle control; Group 3, minocycline (25 mg/kg intratracheal); Group 4, D-LBDiphe (56 mg/kg intratracheal); Group

5, minocycline + D-LBDiphe (10 + 56 mg/kg, intratracheal); Group 6, minocycline + D-LBDiphe (25 + 56 mg/kg, intratracheal); Group 7, colistin (7.5 mg/kg, intravenous). To render mice neutropenic, 4 days prior to the desired date of infection, each mouse was dosed with a single intraperitoneal injection of cyclophosphamide equivalent to 150 mg/kg and returned to its cage. A day prior to infection, each mouse was intraperitoneally dosed with 100 mg/kg of cyclophosphamide. 50 µL of 10^5 CFU/mL (5×10^3 CFU) of PAO1 was introduced deep into the trachea by i.t. intubation using BIOLITE Intubation system (Brain scientific). Mice received intratracheal treatments (Groups 2-6) and intravenous treatment (Group 7), 4 hours post infection. For Group 1, animals were euthanized 4 hours post infection by an overdose of CO_2, lungs were aseptically excised, weighed and placed into 1 mL of Casein soybean digest broth and homogenized. Serial 10-fold dilutions of the lung homogenates were plated onto agar plates, bacterial colonies enumerated after 24 h and bacterial count estimated as log CFU/g lung. This process was followed for Groups 2-7, 28 hours post infection. Significant differences between group means and control were analysed by One way ANOVA, followed by a Dunnett's multiple comparison test, using Graphpad Prism at 95% confidence levels. A P value of < 0 .05 was considered as significant.

References

1. Dhanda, G.; Mukherjee, R.; Basak, D.; Haldar, J., Small-Molecular Adjuvants with Weak Membrane Perturbation Potentiate Antibiotics against Gram-Negative Superbugs. *ACS Infect. Dis.* **2022,** *8* (5), 1086-1097.

2. Konai, M. M.; Ghosh, C.; Yarlagadda, V.; Samaddar, S.; Haldar, J., Membrane active phenylalanine conjugated lipophilic norspermidine derivatives with selective antibacterial activity. *J. Med. Chem.* **2014,** *57* (22), 9409-9423.

3. Sarkar, P.; Basak, D.; Mukherjee, R.; Bandow, J. E.; Haldar, J., Alkyl-Aryl-Vancomycins: Multimodal Glycopeptides with Weak Dependence on the Bacterial Metabolic State. *J. Med. Chem.* **2021,** *64* (14), 10185-10202.

4. Uppu, D. S.; Haldar, J., Lipopolysaccharide Neutralization by Cationic-Amphiphilic Polymers through Pseudoaggregate Formation. *Biomacromolecules* **2016,** *17* (3), 862-873.

5. Carfrae, L. A.; Rachwalski, K.; French, S.; Gordzevich, R.; Seidel, L.; Tsai, C. N.; Tu, M. M.; MacNair, C. R.; Ovchinnikova, O. G.; Clarke, B. R.; Whitfield, C.; Brown, E. D., Inhibiting fatty acid synthesis overcomes colistin resistance. *Nat. Microbiol.* **2023,** *8* (6), 1026-1038.

6. Uppu, D. S.; Manjunath, G. B.; Yarlagadda, V.; Kaviyil, J. E.; Ravikumar, R.; Paramanandham, K.; Shome, B. R.; Haldar, J., Membrane-active macromolecules resensitize

NDM-1 gram-negative clinical isolates to tetracycline antibiotics. *PLoS One* **2015,** *10* (3), e0119422.
7. Dey, R.; De, K.; Mukherjee, R.; Ghosh, S.; Haldar, J., Small antibacterial molecules highly active against drug-resistant Staphylococcus aureus. *Medchemcomm* **2019,** *10* (11), 1907-1915.
8. Lv, B.; Bian, M.; Huang, X.; Sun, F.; Gao, Y.; Wang, Y.; Fu, Y.; Yang, B.; Fu, X., n-Butanol Potentiates Subinhibitory Aminoglycosides against Bacterial Persisters and Multidrug-Resistant MRSA by Rapidly Enhancing Antibiotic Uptake. *ACS Infect. Dis.* **2022,** *8* (2), 373-386.
9. Liu, Y.; Jia, Y.; Yang, K.; Li, R.; Xiao, X.; Zhu, K.; Wang, Z., Metformin Restores Tetracyclines Susceptibility against Multidrug Resistant Bacteria. *Adv. Sci. (Weinh)* **2020,** *7* (12), 1902227.
10. Pogliano, J.; Osborne, N.; Sharp, M. D.; Abanes-De Mello, A.; Perez, A.; Sun, Y. L.; Pogliano, K., A vital stain for studying membrane dynamics in bacteria: a novel mechanism controlling septation during Bacillus subtilis sporulation. *Mol. Microbiol.* **1999,** *31* (4), 1149-1159.
11. Datta, A.; Yadav, V.; Ghosh, A.; Choi, J.; Bhattacharyya, D.; Kar, R. K.; Ilyas, H.; Dutta, A.; An, E.; Mukhopadhyay, J.; Lee, D.; Sanyal, K.; Ramamoorthy, A.; Bhunia, A., Mode of Action of a Designed Antimicrobial Peptide: High Potency against Cryptococcus neoformans. *Biophys. J.* **2016,** *111* (8), 1724-1737.
12. Boags, A.; Hsu, P. C.; Samsudin, F.; Bond, P. J.; Khalid, S., Progress in Molecular Dynamics Simulations of Gram-Negative Bacterial Cell Envelopes. *J. Phys. Chem. Lett.* **2017,** *8* (11), 2513-2518.
13. Uppu, D.; Konai, M. M.; Baul, U.; Singh, P.; Siersma, T. K.; Samaddar, S.; Vemparala, S.; Hamoen, L. W.; Narayana, C.; Haldar, J., Isosteric substitution in cationic-amphiphilic polymers reveals an important role for hydrogen bonding in bacterial membrane interactions. *Chem. Sci.* **2016,** *7* (7), 4613-4623.
14. Wu, E.L.; Cheng, X.; Jo, S.; Rui, H.; Song, K.C.; Dávila-Contreras, E.M.; Qi, Y.; Lee, J.; Monje-Galvan, V.; Venable, R.M.; Klauda, J.B.; Im, W., CHARMM-GUI Membrane Builder toward realistic biological membrane simulations. *J. Comput. Chem.* **2014**, *35* (27), 1997-2004.
15. Vanommeslaeghe, K.; Hatcher, E.; Acharya, C.; Kundu, S.; Zhong, S.; Shim, J.; Darian, E.; Guvench, O.; Lopes, P.; Vorobyov, I.; Mackerell, A. D., Jr., CHARMM general force field: A force field for drug-like molecules compatible with the CHARMM all-atom additive biological force fields. *J. Comput. Chem.* **2010,** *31* (4), 671-690.

16. Evans, D. J.; Holian, B. L., The Nose–Hoover thermostat. *J. Chem. Phys.* **1985,** *83* (8), 4069-4074.

17. Parrinello, M.; Rahman, A., Polymorphic transitions in single crystals: A new molecular dynamics method. *J. Appl. Phys.* **1981,** *52* (12), 7182-7190.

18. Hess, B.; Bekker, H.; Berendsen, H. J. C.; Fraaije, J. G. E. M., LINCS: A linear constraint solver for molecular simulations. *J. Comput. Chem.* **1997,** *18* (12), 1463-1472.

19. Abraham, M. J.; Murtola, T.; Schulz, R.; Páll, S.; Smith, J. C.; Hess, B.; Lindahl, E., GROMACS: High performance molecular simulations through multi-level parallelism from laptops to supercomputers. *SoftwareX* **2015,** *1-2*, 19-25.

20. Essmann, U.; Perera, L.; Berkowitz, M. L.; Darden, T.; Lee, H.; Pedersen, L. G., A smooth particle mesh Ewald method. *J. Chem. Phys.* **1995,** *103* (19), 8577-8593.

21. Humphrey, W.; Dalke, A.; Schulten, K., VMD: visual molecular dynamics. *J. Mol. Graph.* **1996**, *14* (1), 33-38.

22. Dama, J. F.; Parrinello, M.; Voth, G. A., Well-Tempered Metadynamics Converges Asymptotically. *PRL* **2014,** *112* (24), 240602.

23. Tribello, G. A.; Bonomi, M.; Branduardi, D.; Camilloni, C.; Bussi, G., PLUMED 2: New feathers for an old bird. *Comput. Phys. Commun.* **2014,** *185* (2), 604-613.

24. Konai, M. M.; Adhikary, U.; Samaddar, S.; Ghosh, C.; Haldar, J., Structure-Activity Relationship of Amino Acid Tunable Lipidated Norspermidine Conjugates: Disrupting Biofilms with Potent Activity against Bacterial Persisters. *Bioconjug. Chem.* **2015,** *26* (12), 2442-2453.

25. Konai, M. M.; Haldar, J., Lysine-Based Small Molecule Sensitizes Rifampicin and Tetracycline against Multidrug-Resistant Acinetobacter baumannii and Pseudomonas aeruginosa. *ACS Infect. Dis.* **2020,** *6* (1), 91-99.

26. Ghosh, C.; Harmouche, N.; Bechinger, B.; Haldar, J., Aryl-Alkyl-Lysines Interact with Anionic Lipid Components of Bacterial Cell Envelope Eliciting Anti-Inflammatory and Antibiofilm Properties. *ACS Omega* **2018,** *3* (8), 9182-9190.

27. Issa, R.; Meikle, S. T.; James, S.; Cooper, I. R., Poly(epsilon-lysine) dendrons as modulators of quorum sensing in Pseudomonas aeruginosa. *J. Mater. Sci. Mater. Med.* **2015,** *26* (5), 176.

28. Saini, M.; Gaurav, A.; Kothari, A.; Omar, B. J.; Gupta, V.; Bhattacharjee, A.; Pathania, R., Small Molecule IITR00693 (2-Aminoperimidine) Synergizes Polymyxin B Activity against Staphylococcus aureus and Pseudomonas aeruginosa. *ACS Infect. Dis.* **2023,** *9* (3), 692-705.

29. Buyck, J. M.; Tulkens, P. M.; Van Bambeke, F., Pharmacodynamic evaluation of the intracellular activity of antibiotics towards Pseudomonas aeruginosa PAO1 in a model of THP-1 human monocytes. *Antimicrob. Agents Chemother.* **2013,** *57* (5), 2310-2318.
30. Yarlagadda, V.; Samaddar, S.; Haldar, J., Intracellular activity of a membrane-active glycopeptide antibiotic against meticillin-resistant Staphylococcus aureus infection. *J Glob Antimicrob. Resist.* **2016,** *5,* 71-74.
31. Klobucar, K.; Cote, J. P.; French, S.; Borrillo, L.; Guo, A. B. Y.; Serrano-Wu, M. H.; Lee, K. K.; Hubbard, B.; Johnson, J. W.; Gaulin, J. L.; Magolan, J.; Hung, D. T.; Brown, E. D., Chemical Screen for Vancomycin Antagonism Uncovers Probes of the Gram-Negative Outer Membrane. *ACS Chem. Biol.* **2021,** *16* (5), 929-942.
32. Mohid, S. A.; Sharma, P.; Alghalayini, A.; Saini, T.; Datta, D.; Willcox, M. D. P.; Ali, H.; Raha, S.; Singha, A.; Lee, D.; Sahoo, N.; Cranfield, C. G.; Roy, S.; Bhunia, A., A rationally designed synthetic antimicrobial peptide against Pseudomonas-associated corneal keratitis: Structure-function correlation. *Biophys. Chem.* **2022,** *286,* 106802.
33. Mayer, M.; James, T. L., NMR-based characterization of phenothiazines as a RNA binding scaffold. *J. Am. Chem. Soc.* **2004,** *126* (13), 4453-4460.
34. Bhunia, A.; Bhattacharjya, S., Mapping residue-specific contacts of polymyxin B with lipopolysaccharide by saturation transfer difference NMR: insights into outer-membrane disruption and endotoxin neutralization. *Biopolymers* **2011,** *96* (3), 273-287.
35. Konai, M. M.; Haldar, J., Fatty Acid Comprising Lysine Conjugates: Anti-MRSA Agents That Display In Vivo Efficacy by Disrupting Biofilms with No Resistance Development. *Bioconjug. Chem.* **2017,** *28* (4), 1194-1204.
36. Ji, X.; Yang, X.; Shi, C.; Guo, D.; Wang, X.; Messina, J.M.; Meng, Q.; Urao, N.; Cooney, R.; Luo, J. Functionalized core-shell nanogel scavenger for immune modulation therapy in sepsis. *Adv. Ther. (Weinh)*. **2022**, *5* (10), 2200127.
